# Synthesis of MeBmt and related derivatives *via* syn-selective ATH-DKR†
†Electronic supplementary information (ESI) available: Experimental details and characterization data of all new molecules. CCDC 1897285. For ESI and crystallographic data in CIF or other electronic format see DOI: 10.1039/c9ra08256e


**DOI:** 10.1039/c9ra08256e

**Published:** 2019-12-05

**Authors:** Adam Rolt, Paul M. O'Neill, T. Jake Liang, Andrew V. Stachulski

**Affiliations:** a Department of Biochemistry, University of Oxford, OX1 3QU, UK; b The Robert Robinson Laboratories, Department of Chemistry, University of Liverpool, Liverpool, L69 7ZD, UK. Email: stachuls@liv.ac.uk; c Liver Diseases Branch, National Institute of Diabetes and Digestive and Kidney Diseases, National Institutes of Health, 10 Center Drive, Bethesda, Maryland 20892, USA

## Abstract

The unusual α-amino, β-hydroxy acid MeBmt is a key structural feature of cyclosporin A, an important naturally occurring immunosuppressant and antiviral agent. We present a convergent synthesis of MeBmt which relies on new aspects of dynamic kinetic resolution (DKR) to establish simultaneously the chirality at C(2) and C(3). We also show that this route is applicable to the synthesis of other derivatives.

## Abstract 2

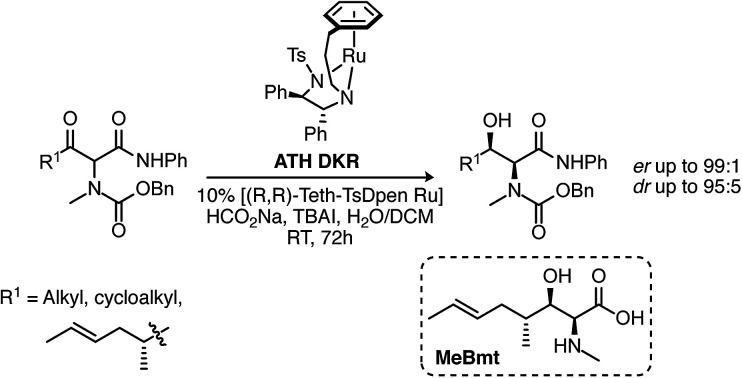
The unusual α-amino, β-hydroxy acid MeBmt is a key structural feature of cyclosporin A, an important naturally occurring immunosuppressant and antiviral agent. We describe a concise synthesis of MeBmt using the principle of dynamic kinetic resolution.

## 

The α-amino-β-hydroxy unit is a familiar structural motif in many important natural products, notably the proteinogenic amino-acids serine and threonine, glycosphingolipids^1^ and more complex structures such as the polyoxins.^2^ We were particularly interested in the cyclic undecapeptide cyclosporin A (CsA) **1**, which contains the unusual amino acid [(2*S*,3*R*,4*R*,6*E*)-3-hydroxy-4-methyl-2-(methylamino)-6-octenoic acid] (MeBmt **2**) at position 1, shown in Fig. 1.

**Fig. 1:**
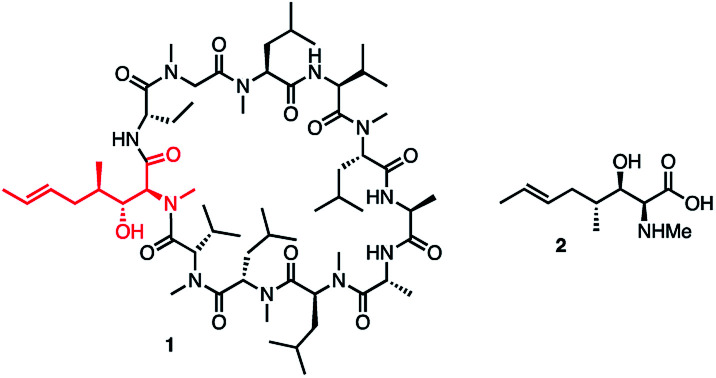
Structures of CsA **1** and MeBmt **2**.

While CsA **1**, first attracted attention as a valuable immuno-suppressant^3^ used especially following transplant surgery, it was later found to have potent antiviral activity, notably against hepatitis C virus (HCV) *via* its inhibition of the proline *cis*–*trans* isomerase, cyclophilin A.^4^–^6^ Synthetic modification can uncouple the antiviral effects from the immunosuppressive effects,^7^,^8^ thus Debio-025 (Alisporivir), a CsA analogue modified at positions 3 and 4, retains excellent activity *vs.* HCV (IC_50_ = 30 nM) and is essentially non-immunosuppressive.^9^ Additionally, reported analogues of **1** modified at the MeBmt residue also show antiviral activity while being essentially inactive as immunosuppressants.^7^ Previous syntheses of **2** were too lengthy and linear to facilitate a medicinal chemistry campaign of CsA around **2**.^10^–^13^ We therefore sought to develop a short effective synthesis of **2**, which would readily permit the synthesis of position 1 analogues of CsA without relying on partial synthesis. Thus, our retrosynthesis of MeBmt **2** is shown in Scheme 1, it relies on the syn-selective dynamic kinetic resolution (DKR) of a β-ketoester precursor **3**, which is in turn accessible *via* a crossed Claisen condensation^14^ of an activated form of carboxylic acid **4** with protected sarcosine ester **5**.

**Scheme 1:**
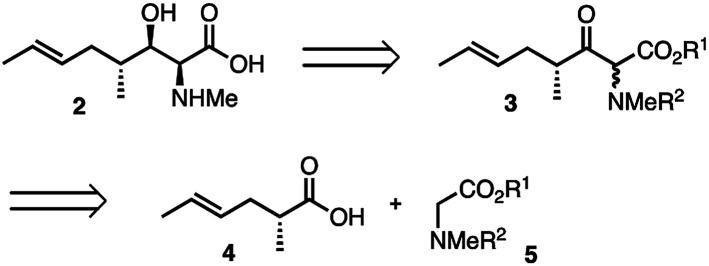
Proposed disconnections in the synthesis of MeBmt. R^1/2^ = protecting groups.

Several groups have effected DKR under catalytic asymmetric transfer hydrogenation (ATH)^15^ conditions, the stereochemical outcome is dependent on substrate structure. The synthesis of β-hydroxy amino acid derivatives *via* ATH DKR is usually undertaken *via* reduction from the prerequisite β-keto methyl ester and the stereochemical outcome tends to the 2,3-anti-product.^16^,^17^ This anti diastereoselectivity is proposed to be the product of intramolecular hydrogen bonding.^18^ In contrast, γ-aryl-*N*-Me substrates underwent ATH DKR to give the desired syn-products,^19^ though this had not been demonstrated with γ-alkyl substrates. We therefore set out to study these ATH-DKR conditions, initially on a model isobutyryl substrate **6a**, and we now report our findings, leading eventually to a concise synthesis of MeBmt.

Compound **6a** (Scheme 2) was prepared by a crossed Claisen condensation between isobutyryl chloride and sarcosine derivative **7a** (see ESI†); alternatively, using the corresponding glycine ester **7b**, **6b** was readily obtained.^14^

**Scheme 2:**
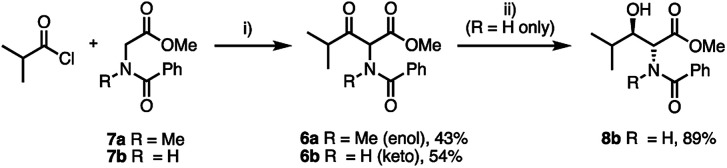
Synthesis of α-acylamino-β-keto-esters and ATH DKR discrepancy. (i) TiCl_4_, Bu_3_N, *N*-Me imidazole, CH_2_Cl_2_, –40 °C; (ii) HCO_2_Na, TBAI, CH_2_Cl_2_/H_2_O; 3% Ru(*p*-cymene)[(*R*,*R*)-TsDPEN], 20 °C, 20 h. Isolated yields are shown.

Branching of the terminal alkyl substituent in **6a**/**6b** has been shown to improve stereoselectivity in ATH DKR of similar substrates^18^ thus **6a**/**6b** are good models for MeBmt. Substrate **6b** was reduced efficiently *via* ATH DKR to the (2*R*,3*R*)-anti product **8b** (Table 1), however under these conditions the *N*-Me substrate **6a**, which was isolated almost entirely as the enol tautomer (>95%, NMR), was unreactive towards ATH DKR. Attempted optimisation of the ATH DKR step with increased catalyst loading, elevated temperatures, and replacement of *N*-benzoyl with other N-protecting groups (*viz.* CBz (**6c**)) and alternative ATH DKR conditions^16^,^20^,^21^ led to the same result. In contrast, related γ-aryl-β-keto esters bearing an *N*-Me group are reported to be efficiently reduced.^19^

**Table 1:** Summary of ATH DKR reactivity across key substrates. **A** X-ray crystal structure of (2*S*, 3*R*)-**17**. **B** ATH catalyst [(*R*,*R*)-Teth-TsDpen] used in entry 7

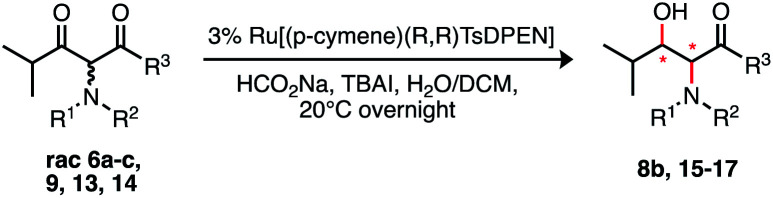									
Entry	Cmpd	R^1^	R^2^	R^3^	Keto : enol	Product	Isolated yield (%)	dr^*a*^	er^*b*^
1	**6b**	H	Bz	OMe	>95 : 5	**8b**	82	15 : 85	ND
2	**6a**	Me	Bz	OMe	>5 : 95	—	NR	ND	ND
3	**6c**	Me	CBz	OMe	>5 : 95	—	NR	ND	ND
4	**13**	Me	Bz	NHPh	28 : 72	**15**	4	>95 : 5	ND
5	**14**	Me	FMOC	NHPh	25 : 75	**16**	18	>95 : 5	>99 : 1
6	**9**	Me	CBz	NHPh	30 : 70	**17**	13	>95 : 5	>99 : 1
7^*c*^	**9**	Me	CBz	NHPh	30 : 70	**17**	64	>95 : 5	>99 : 1
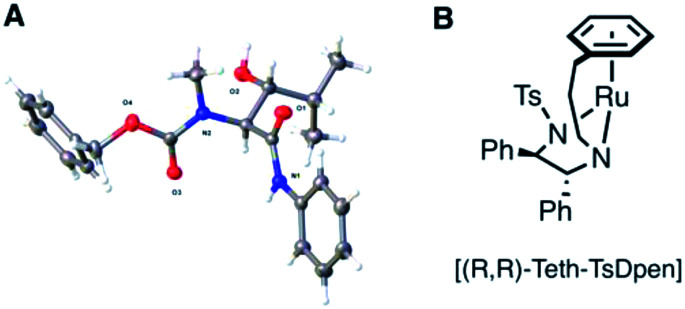									

^*a*^Syn : anti ratio (^1^H NMR).

^*b*^Ratio of (2*S*,3*R*) : (2*R*,3*S*) enantiomers.

^*c*^10% [(*R*,*R*)-Teth-TsDpen], 40 h, 20 °C.

We considered that the previously proposed intramolecular hydrogen bonding^18^ (Fig. 2A and B) of **6b** could also be responsible for the reactivity of the compounds in general through stabilisation or activation of the keto tautomer. The successful syn-reduction of a γ-aryl-*N*-Me, examples is, we believe, largely due to the great preference for the keto tautomer in such examples.

**Fig. 2:**
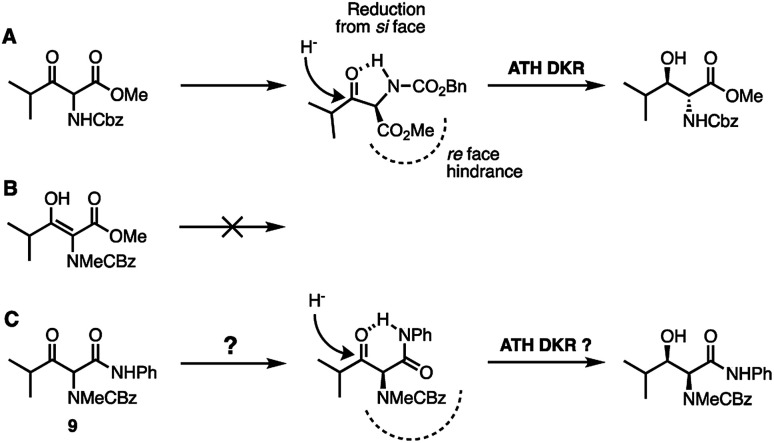
(A) Proposed intramolecular H-bonding accounting for the anti-reduction of **6b**^18^ (B) removing the H-bond leads to a completely unreactive enol tautomer; (C) restoring an intra-H bond *via* an anilide.

In contrast, our earlier γ-alkyl-*N*-Me, substrate **6a** existed almost entirely as the enol tautomer (>95 : 5 enol) by ^1^H NMR. We therefore proposed that a favourable intramolecular H-bond could be reintroduced by employing an anilide rather than an ester, in a substrate such as **9**, Fig. 2C, ensuring a significant percentage of the necessary keto tautomer shown, indeed β-keto anilides have been utilized in ATH DKR previously though not in this context.^22^ Thus, the anilides were prepared according to Scheme 3.

**Scheme 3:**
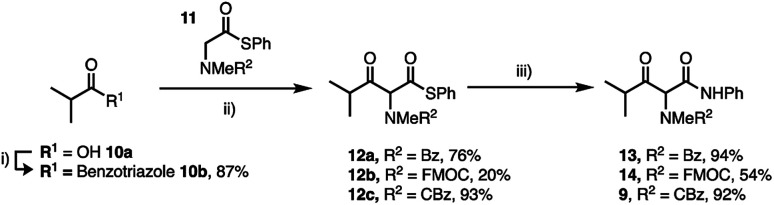
Synthesis of γ-alkyl-β-keto-anilides (i) 1*H*-benzotriazole, SOCl_2_, DCM, 20 °C (ii) MgBr_2_·OEt_2_, ^i^PrNEt_2_, DCM, 0–20 °C, 16 h (iii) PhNH_2_, AgCO_2_CF_3_, THF, 20 °C, 16 h. Isolated yields are shown.

Thioesters such as **11** are excellent nucleophiles in the crossed Claisen condensation with *N*-acyl benzotriazoles (**10b**) and deliver thioesters in very good yield other than FMOC derivative **12b** which underwent base catalysed degradation.^23^ Finally, Ag^+^-catalysed aminolysis of the thioesters **12a–c** delivered the desired anilides **9**, **13** and **14** in high yield. The anilides were isolated with a more favourable keto : enol ratio of ∼30 : 70 depending on R^2^ (determined by NMR in CDCl_3_).

Accordingly, incorporation of the anilide in the isobutyryl series both re-establishes reactivity and reverses the original anti diastereoselectivity, delivering the syn product **15**, although in low isolated yield (entries 4–6, Table 1). Other *N*-substituents, *viz. Z*, entry 5, and Fmoc, entry 6, were also compatible with these conditions, though Fmoc proceeds with much lower yields in the preceding crossed-Claisen step, presumably due to decomposition under basic conditions. To permit a practical transformation, we screened reaction conditions and a number of commercially available catalysts and ligands to increase yield and stereoselectivity^17^,^24^–^28^ (see ESI†). The most efficient proved to be the tethered catalyst described by Wills *et al.*; entry 7 and illustrated in Table 1 **B**, this delivered a 64% isolated yield of syn-**17** after 40 h, in >95 : 5 dr and >99 : 1 er with full consumption of starting material. The full restoration of DKR reaction with just 25–30% keto tautomer in the precursor is emphasized.

γ-Alkyl-β-hydroxy anilide **17** was crystallised in a form suitable for single crystal X-ray structure determination, confirming the absolute configuration; shown in Table 1. Replacement of the anilide with benzylamide did not permit ATH DKR, presumably because of the lower acidity of the resultant NH. With the DKR step optimized for the model substrate, we proceeded to complete the synthesis of MeBmt **2**, Scheme 4. Based on a method originally reported by Rich *et al.*^29^ starting from (±)-3-buten-2-ol **18**, a Johnson–Claisen rearrangement followed by transformation of the crude ethyl ester gave (*S*,*R*)-phenylglycinol amide **19**, which could be separated from its (*S*,*S*) diastereomer by gradient column chromatography in multigram amounts. The amide was quantitatively hydrolysed under acid catalysis aided by neighbouring OH-group participation to afford carboxylic acid (2*R*)-**20**. Conversion to the activated *N*-acyl-benzotriazolyl (Bt) electrophile^30^ (2*R*)-**21** (stable at room temperature in air), followed by a crossed Claisen condensation with sarcosine derivative **22** (see ESI†), afforded enol thioester **23**, which was converted to β-keto-anilide **24** in excellent yield under mild conditions. Syn selective ATH DKR afforded β-hydroxy-amide **25**, without observable epimerization at C4 (judged by ^1^H NMR). Base hydrolysis of **25** led to universal deprotection,^31^ yielding MeBmt **2** without any detectable racemization (^1^H NMR).

**Scheme 4:**
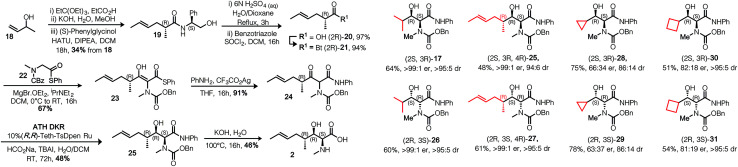
Synthesis of MeBmt *via* ATH DKR and demonstration of substrate scope. Analogues were synthesized according to the optimised procedures. Isolated yield following aqueous workup and column chromatography are shown, dr is expressed as syn : anti in crude product, er is expressed as major : minor in purified major diastereomer. Absolute stereochemistry in products is dependent on catalyst configuration.

Finally, in Scheme 4 we demonstrate that the route is applicable to the synthesis of several γ-alkyl-α-*N*-Me derivatives including model isopropyl examples **17** and **26**, MeBmt precursor **25** and isomer **27.** Structurally novel cycloalkyl examples **28–31** were also synthesized starting from their constituent carboxylic acids (see ESI†). Absolute stereochemistry is controlled by using the opposite enantiomer of catalyst, in all cases the major and minor diastereomers could be separated by column chromatography. From this small set, it can be seen that stereoselectivity increases as the steric bulk of the side chain increases across the series. Interestingly, a match/mismatch effect in the reduction of **24** with (*S*,*S*)-Teth-TsDPEN was observed: reduction from the re-face of the molecule was slower than reduction from the si-face (*cf.* **25** and **27**), judged by TLC, which was also reflected in the isolated yields and the diastereoselectivity. This is expected to be a consequence of the increased steric bulk of the (4*R*)-butenyl group in substrate **24** producing facial discrepancies in catalyst approach.

In conclusion, through analysis of intramolecular hydrogen bonding, we have expanded the scope of the ATH DKR reaction, giving access to biologically relevant γ-alkyl-β-hydroxy-α-Me-amino acids from readily accessible β-keto precursors. This was accomplished by switching from β-keto-esters to β-keto-anilides, which was sufficient to restore reactivity and reverse the original anti diastereoselectivity, delivering the syn products. Accordingly, we demonstrated a synthesis of MeBmt, **2** in five linear steps from precursor carboxylic acid (2*R*)-**20** and have further shown that this route is also applicable for the synthesis of alternative γ-alkyl derivatives. The route is reasonably short, while also being modular and flexible in terms of side chain choice and absolute stereochemical outcome.

## Conflicts of interest

There are no conflicts to declare.

## Supplementary Material

Supplementary information

Crystal structure data
