# Supplementary material for: Synthesis of MeBmt and related derivatives via syn-selective ATH-DKR
Source: RSC Adv. 2019 Dec 5;9(69):40336–9. doi: 10.1039/c9ra08256e (PMC7437948; doi:10.1039/c9ra08256e)
Supplement: RA-009-C9RA08256E-s001 [file RA-009-C9RA08256E-s001.pdf]

Electronic Supplementary Material (ESI) for RSC Advances.
This journal is © The Royal Society of Chemistry 2019

Supplemental Information

Enantioselective synthesis of γ-alkyl β-hydroxy α-amino acids via asymmetric transfer hydrogenation: application to MeBmt

Adam Rolt[1], Paul M. O'Neill[2], T. Jake Liang[3], and Andrew V. Stachulski[2]*

[1]Department of Biochemistry, University of Oxford, OX1 3QU

[2]The Robert Robinson Laboratories, Department of Chemistry, University of Liverpool, Liverpool, L69 7ZD, UK

[3]Liver Diseases Branch, National Institute of Diabetes and Digestive and Kidney Diseases, National Institutes of Health, 10 Center Drive, Bethesda, Maryland 20892, USA

**Contents**	**Page**
General Information	2
Supplementary Figure 1	4
Supplementary Table 1 (S1)	5
General synthetic procedures	6
Synthesis and characterization of new compounds	8
Copies of NMR spectra and HRMS reports	24
Stereochemical analysis of **17** and chiral HPLC chromatograms (**17, 25-31**)	63
Single crystal X-ray diffraction of 2S, 3R **- 17**	81
References	84

All air or moisture sensitive reactions were performed under a positive pressure of nitrogen, using oven-or flame dried glassware where stated. Anhydrous solvents were purchased from Sigma-Aldrich (St. Louis, MO). 'Normal phase chromatography', column chromatography, or gradient column chromatography refers to automatic purification using a 'Tyledyne ISCO CombiFlash® Rf+' system with pre-loaded silica gel 'Redisep RF flash columns' of an appropriate size. Preparative HPLC purification was performed on a Waters semi-preparative HPLC system (Waters Corp., Milford, MA). The column used was a Phenomenex Luna C18 (5 micron, 30 x 75 mm; Phenomenex, Inc., Torrance, CA) at a flow rate of 45.0 mL/min. The mobile phase consisted of acetonitrile and water (each containing 0.1% trifluoroacetic acid). A gradient of 10% to 50% acetonitrile over 8 min was used during the purification. Automated fraction collection was triggered by UV detection at 220 nM. Analytical analysis was performed on an Agilent LC/MS (Agilent Technologies, Santa Clara, CA). A Phenomenex Luna C18 column (3 micron, 3 x 75 mm) was used at a temperature of 50 °C. Purity determination was performed using an Agilent diode array detector for both Method 1 and Method 2.

Method 1: A 7-min gradient of 4% to 100% acetonitrile (containing 0.025% trifluoroacetic acid) in water (containing 0.05% trifluoroacetic acid) was used with an 8-min run time at a flow rate of 1.0 mL/min.

Method 2: A 3-min gradient of 4% to 100% acetonitrile (containing 0.025% trifluoroacetic acid) in water (containing 0.05% trifluoroacetic acid) was used with a 4.5-min run time at a flow rate of 1.0 mL/min. Retention time (RT) and m/z for M+1 is reported, occasionally (M+Na) is observed exclusively.

Mass determination was performed using an Agilent 6130 mass spectrometer with electrospray ionization in the positive mode.

^{1}H and ^{13}C NMR spectra were recorded on Varian 400 MHz spectrometers (Agilent Technologies, Santa Clara, CA). Signals are described as singlets (s), doublets (d), triplets (t), quadruplets (q), septuplets (sept), doublet of doublets (dd) and doublet of triplets (dt). Spectra were analysed in MestReNova.

Single-crystal X-ray diffraction studies were carried out by Dr. Curtis Moore at the UCSD Crystallography Lab using a Bruker D8 Platinum[135] CCD diffractometer equipped with Cu K_{α} radiation (λ = 1.5478). A 0.217 x 0.053 x 0.051 mm piece of a colorless rod was mounted on a Cryoloop with Paratone oil. Data were collected in a nitrogen gas stream at 125(2) K using ϕ and ϖ scans. Crystal-to-detector distance was 45 mm using variable exposure time (2s-5s) depending on θ with a scan width of 2.0°. Data collection was 98.4% complete to 68.00° in θ. A total of 33864 reflections were collected covering the indices, -11<=h<=10, -12<=k<=14, -19<=l<=19. 3547 reflections were found to be symmetry independent, with a R_{int} of 0.0549. Indexing and unit cell refinement indicated a primitive, orthorhombic lattice. The space group was found to be P212121. The data were integrated using the Bruker SAINT software program and scaled using the SADABS software program. Solution by direct methods (SHELXT) produced a complete phasing model consistent with the proposed structure.

All non-hydrogen atoms were refined anisotropically by full-matrix least-squares (SHELXL-2014). All carbon bonded hydrogen atoms were placed using a riding model. Their positions were constrained relative to their parent atom using the appropriate HFIX command in SHELXL-2014. All other hydrogen atoms (H-bonding) were located in the difference map. Their relative positions were restrained using DFIX commands and their thermals freely refined. The absolute stereochemistry of the molecule was established by anomalous dispersion using the Parson's method with a Flack parameter of 0.050(34).

Chiral chromatographic analysis was performed by Agilent 1200 series HPLC using an analytical Chiralpak AD column (4.6 mm x 250 mm; 5 μm). The mobile phase consisted of ethanol and hexanes containing 0.1% diethylamine.

Optical rotations were measured on a PerkinElmer model 341 polarimeter with a 10cm cell

Supplementary Figure 1: Preparation of β-keto esters and amides

Synthesis of β-keto esters, structures 6a-c

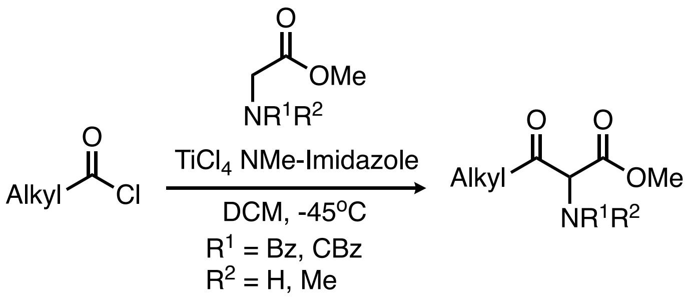

Synthesis of β-keto anilides, structures 15-17

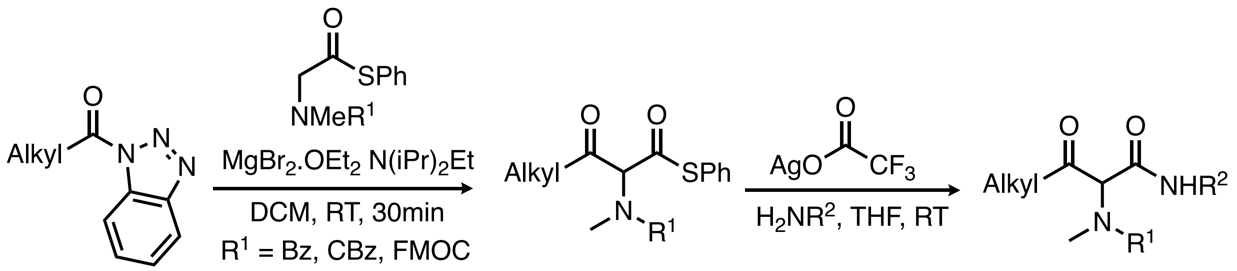

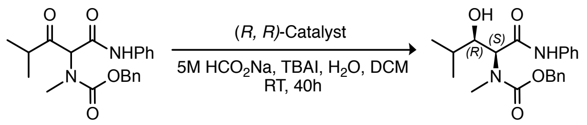

Entry	Cat.	mol % cat.	Time (h)	Yield %[a]	dr[b]	er[c]
**1**	A	5	40	36	>95:5	>99:1
**2**	B	5	40	34	>95:5	96:4
**3**	C	5	40	36	>95:5	97:3
**4**	D	5	40	40	>95:5	98:2
**5**	E	5	40	42	>95:5	96:4
**6**	**F**	**5**	**40**	**64**	**>95:5**	**>99:1**
**7**	F	5	120	ND	ND	ND
**8**	F	10	40	64	>95:5	>99:1

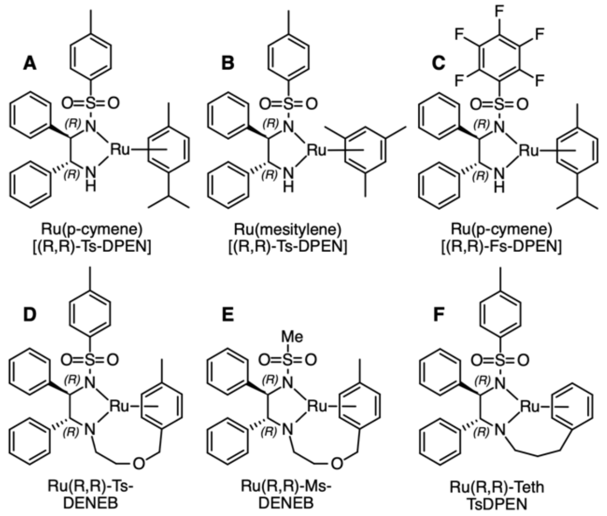

Supplementary Table S1. Survey of catalysts for optimization of ATH DKR. ND - Not determined, [a]Isolated yield following aqueous workup and column chromatography dr - diastereomer ratio (2S,3R):(2R,3R), er - enantiomer ratio (2S,3R):(2R,3S). Structures of catalysts **A-F** given below table. **Entry 7**: Long reaction times resulted in decomposition of product. Full consumption of starting material was noted for entries **7** and **8**.

General procedures

General procedure 'A' for Ti-Crossed Claisen[1]

In general, an acyl chloride (25.4 mmol) was added to a stirring solution of a glycine derivative (25.4 mmol) and 1-methyl-1H-imidazole (2.426 mL, 30.4 mmol) in DCM (50 mL) at -45 °C under N_2. Stirring was continued for 10 minutes, then $TiCl_4$ (9.79 mL, 89 mmol) and diisopropylethylamine (17.72 mL, 101 mmol) were successively added to the mixture which was stirred at -45 °C for a further 1 hour. The mixture was quenched with water (100 mL), then extracted with EtOAc (2x). The combined extracts were washed with water, brine, dried over $MgSO_4$ filtered and concentrated *in vacuo*. Purification by gradient normal phase chromatography yielded the title compound.

General procedure 'B' for thioester formation

In general, to the carboxylic acid (22.40 mmol) in DCM (50 mL) at 0°C was added isobutyl chloroformate (3.22 mL, 24.64 mmol), benzenethiol (5.03 mL, 49.3 mmol) and triethylamine (6.24 mL, 44.8 mmol). The reaction was allowed to warm to room temperature and stirred until completion. The reaction was diluted with Et_2O (30 mL) to facilitate precipitation of triethylamine HCl, the salt was filtered off and the filter cake was washed with Et_2O, (50 mL). Volatiles were removed under rotary evaporation. The residue was taken up in EtOAc, and washed with 1N HCl, 1N NaOH, and brine. Organics were dried over $MgSO_4$ and concentrated in vacuo, followed by purification by gradient normal phase chromatography to afford the thoioester.

General procedure 'C' for thioester crossed Claisen[2]

In general, under N_2 at 0°C, magnesium bromide diethyl etherate (1.27 g, 4.91 mmol) was added to a stirred solution of the respective N-acyl benzotriazole (1.402 mmol) in DCM (6 mL), followed by the addition of the respective thioester nucleophile (1.402 mmol) and diisopropylethylamine (0.979 mL, 5.61 mmol). The ice-bath was removed, and the reaction was allowed to warm to ambient temperature, then left to stir overnight (18h). Upon completion, the reaction was again cooled to 0-5°C, then 10% aqueous HCl (8 mL) was added dropwise. Stirring was continued for 5 min and the mixture was partitioned between EtOAc (30 mL) and H_2O (20 mL). The aqueous phase was extracted with EtOAc (2 x 20 mL) and the combined organic extracts were washed with brine, dried over $MgSO_4$, and concentrated *in vacuo*. The crude β-ketoester products were are purified by gradient column chromatography.

General procedure 'D' for ATH DKR in emulsions[3]

Under N_2, to a solution of an appropriate β-keto derivative (7.60 mmol) in DCM (25 mL) was added sodium formate solution (5N, 50 mL) followed by tetrabutylammonium iodide (5.61 g, 15.19 mmol) and pre-activated Ru-ATH DKR catalyst (0.760 mmol, 10 % loading). After stirring for 40 hours the reaction was added to a separatory funnel and diluted with EtOAc and sat. $NaHCO_3$, then the organic phase was separated and the aqueous was extracted with twice more with EtOAc. The combined extracts were dried ($MgSO_4$) and the crude oil was purified by gradient column chromatography, yielding β-hydroxy reduction products.

General procedure 'E' for thioester substitution[2]

To a stirred solution of the appropriate β-keto thioester (0.281 mmol) and aniline (31.4 mg, 0.338 mmol) in THF (2 mL) was added silver trifluoroacetate (63.4 mg, 0.309 mmol). The reaction was stirred until complete, diluted with 20mL of EtOAc and passed through a pad of Celite to remove silver salts. The filtrate was concentrated followed by purification by gradient flash chromatography over silica gel affording the corresponding β-keto anilide.

General procedure 'F' for synthesis of N-acyl benzotriazoles[4]

$SOCl_2$ (157 µl, 2.146 mmol) was added to a stirred solution of 1H-benzo[d][1,2,3]triazole (929 mg, 7.80 mmol) in DCM (9 mL), in one portion at room temperature; stirring was continued for 30 min. The appropriate carboxylic acid (1.951 mmol) in DCM (1 mL) was added to the reaction mixture, followed by almost immediate precipitation of 1H-benzo[d][1,2,3]triazole HCl. Stirring was continued overnight, then the white precipitate was filtered off and washed with DCM. The combined DCM filtrates were evaporated and the crude N-acylbenzotriazole was purified by silica gel flash chromatography.

Methyl-3-hydroxy-4-methyl-2-(N-methylbenzamido)pent-2-enoate, 6a

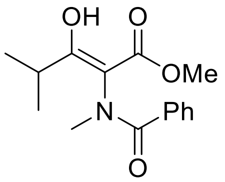

Following general procedure **A**, isobutyryl chloride (0.506 mL, 4.83 mmol) was coupled to N-benzoyl-N-methylglycinate (1.0 g, 4.83 mmol) with 1-methyl-1H imidazole (0.462 mL, 5.79 mmol), $TiCl_4$ (1.862 mL, 16.89 mmol) and diisopropylamine (3.37 mL, 19.30 mmol) in DCM (10 mL) at -45 °C under N_2. The crude oil was purified by column chromatography to yield methyl-3-hydroxy-4-methyl-2-(N-methylbenzamido)pent-2-enoate **6a** as a white solid (0.575 g, 2.075 mmol, 43 % yield)

LCMS RT (Method 2) 3.24-3.4 min (m/z 278.1)

^{1}H NMR (400 MHz, $CDCl_3$) δ 12.16 (d, *J* = 1.5 Hz, 1H), 7.51 – 7.23 (m, 6H), 3.83 – 3.80 (m, 3H), 3.16 (s, 3H), 2.81 – 2.70 (m, 1H), 1.11 (d, *J* = 6.8, 3H), 0.77 (d, *J* = 6.7, 3H)

^{13}C NMR (101 MHz, $CDCl_3$) δ 180.97, 172.64, 170.86, 136.07, 129.72, 127.92, 126.74, 108.16, 52.16, 37.32, 29.15, 20.38, 17.72.

HRMS (ESI) *m/z* calcd for $[M + H]^+$ 278.1392, found 278.1384.

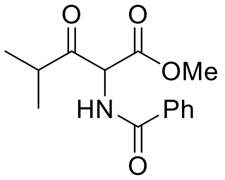

Methyl 2-benzamido-4-methyl-3-oxopentanoate, 6b

Following general procedure **A**, isobutyryl chloride (2.66 mL, 25.4 mmol) was coupled with methyl 2-benzamidoacetate (4.9 g, 25.4 mmol) using 1-methyl-1H-imidazole (2.426 mL, 30.4 mmol), $TiCl_4$ (9.79 mL, 89 mmol) and diisopropylethylamine (17.72 mL, 101 mmol). Purification by gradient normal phase chromatography afforded **6b** (3.61 g, 13.70 mmol, 54 % yield) as a colourless oil.

LCMS RT (Method 2) 3.14 min (m/z 264.1)

^{1}H NMR (400 MHz, $CDCl_3$) δ 7.84 – 7.79 (m, 2H), 7.52 – 7.46 (m, 1H), 7.44 – 7.38 (m, 2H), 7.35 (d, *J* = 6.5 Hz, 1H), 5.59 (d, *J* = 6.7 Hz, 1H), 3.78 (s, 3H), 3.19 – 3.02 (m, 1H), 1.21 (d, *J* = 7.1 Hz, 3H), 1.11 (d, *J* = 6.7 Hz, 3H).

^{13}C NMR (101 MHz, $CDCl_3$) δ 205.02, 166.94, 166.76, 132.98, 132.05, 128.59, 127.23, 61.03, 53.26, 38.76, 18.81, 17.66.

HRMS (ESI) *m/z* calcd for $[M + H]^+$ 264.1230, found 264.1226.

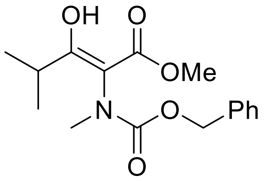

Methyl-2-(((benzyloxy)carbonyl)(methyl)amino)-3-hydroxy-4-methylpent-2-enoate, 6c

Following general procedure **A**, isobutyryl chloride (0.442 mL, 4.21 mmol) was coupled to methyl N- ((benzyloxy) carbonyl)-N-methylglycinate (1.0 g, 4.21 mmol) with 1-methyl-1H-imidazole (0.403 mL, 5.06 mmol), $TiCl_4$ (1.627 mL, 14.75 mmol) and diisopropylethylamine (2.94 mL, 16.86 mmol) at -45 °C under N_2. The crude oil was purified by column chromatography to yield methyl-2-(((benzyloxy)carbonyl)(methyl)amino)-3-hydroxy-4-methylpent-2-enoate, **6c** (0.492 g, 1.602 mmol, 38 % yield) as a colourless oil**.**

LCMS RT (Method 2) 3.64 min (m/z 308.1)

^{1}H NMR (400 MHz, $CDCl_3$) enol (major) δ 12.19 (s, 1H), 7.49 – 7.11 (m, 5H), 5.17 – 5.01 (m, 2H), 3.77 – 3.62 (m, 3H), 3.03 – 2.95 (m, 3H), 2.84 – 2.75 (m, 1H), 1.13 – 0.97 (m, 6H).

^{13}C NMR (101 MHz, $CDCl_3$) δ 206.64, 180.66, 171.11, 156.63, 136.69, 128.33, 127.87, 127.74, 105.57, 67.21, 51.81, 37.72, 29.11, 18.88.

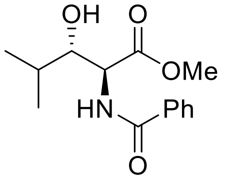

Methyl (2S,3S)-2-benzamido-3-hydroxy-4-methylpentanoate, 8b

Following general procedure **D**, methyl 2-benzamido-4-methyl-3-oxopentanoate **6b** (2 g, 7.60 mmol) underwent ATH DKR with pre-activated Ru(p-cymene)[(S,S)-Ts-DPEN] (0.140 g, 0.228 mmol, 3 % loading). The crude oil was purified by gradient column chromatography yielding (2S,3S)-methyl 2-benzamido-3-hydroxy-4-methylpentanoate **8b** (1.794 g, 6.76 mmol, 89 % yield), a colourless oil, as an inseparable mixture of *syn* and *anti* diasteromers (15:85 by NMR). Major isomer (anti) reported:

LCMS RT (Method 2) 2.89 min (m/z 266.1)

^{1}H NMR (400 MHz, $CDCl_3$) δ 7.82 – 7.75 (m, 2H), 7.52 – 7.43 (m, 1H), 7.43 – 7.34 (m, 2H), 7.29 – 7.20 (m, 1H), 4.93 (dd, *J* = 7.6, 3.4 Hz, 1H), 3.76 (s, 3H), 3.63 – 3.51 (m, 1H), 3.27 (d, *J* = 7.7 Hz, 1H), 1.77 (dsept, *J* = 8.5, 6.7 Hz, 1H), 0.99 (t, *J* = 6.5 Hz, 6H).

^{13}C NMR (101 MHz, $CDCl_3$) δ 171.37, 167.41, 133.41, 131.91, 128.57, 127.16, 78.71, 55.80, 52.51, 31.38, 19.08.

HRMS (ESI) *m/z* calcd for $[M + H]^+$ 266.1392, found 266.1327.

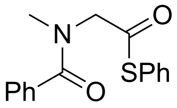

***S*-phenyl 2-(*N*-methylbenzamido)ethanethioate**

Following general procedure **B,** N-benzoyl-N-methylglycine (3 g, 15.53 mmol) was transformed into its phenylthioester derivative using, isobutyl chloroformate (2.333 g, 17.08 mmol), triethylamine (3.142 g, 31.6 mmol) and thiophenol (3.76 g, 34.2 mmol). After workup, the crude was purified by gradient column chromatography to yield the title compound (3.54 g, 12.42 mmol, 80 % yield) as a colourless oil, which solidified on standing.

LCMS RT (Method 2) 3.55 min (m/z 308.1)

^{1}H NMR (400 MHz, $CDCl_3$) δ 7.73 – 7.31 (m, 10H), 4.55 (s, 1.4H), 4.24 (s, 0.6 H), 3.13 (s, 3H).

^{13}C NMR (101 MHz, $CDCl_3$) δ 194.86, 171.97, 135.18, 134.76, 130.02, 129.67, 129.34, 128.48, 127.07, 57.02, 39.14.

HRMS (ESI) *m/z* calculated for $[M + Na]^+$ 308.0716, found 308.0720.

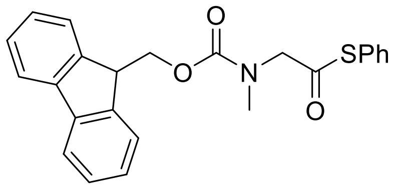

S-phenyl 2-((((9H-fluoren-9yl)methoxy)carbonyl)(methyl)amino) ethanethioate

Following general procedure **B**, 2-((((9H-fluoren-9- yl) methoxy)carbonyl)(methyl)amino)acetic acid (5 g, 16.06 mmol) was transformed into its derived phenyl thioester with isobutyl chloroformate (2.309 mL, 17.67 mmol) benzenethiol (3.60 mL, 35.3 mmol) and triethylamine (4.48 mL, 32.1 mmol). Following aqueous work-up the material was purified by gradient column chromatography (0-30 % EtOAc in hexanes) to afford the title compound (1.07 g, 2.65 mmol, 17 % yield)

LCMS RT (Method 2) 3.83 min (m/z 404.1 (M+H), 426.1 (M+Na))

^{1}H NMR (400 MHz, $CDCl_3$) δ 7.76 (dd, *J* = 7.7, 3.0 Hz, 2H), 7.60 (dd, *J* = 18.7, 7.5 Hz, 2H), 7.44 – 7.24 (m, 9H), 4.53 – 4.44 (m, 2H), 4.29 (s, 1H), 4.34 – 4.21 (m, 1H), 4.16 (s, 1H), 3.08 (d, *J* = 1.9 Hz, 3H).

^{13}C NMR (101 MHz, $CDCl_3$) δ 196.0, 143.81, 141.31, 134.70, 134.60, 129.57, 129.27, 127.71, 127.08, 125.01, 124.93, 119.99, 68.05, 58.61, 47.23, 36.50.

HRMS (ESI) *m/z* calcd for $[M + H]^+$ 404.1315, found 404.1298.

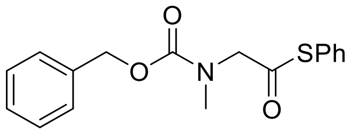

S-phenyl 2- (((benzyloxy)carbonyl)(methyl)amino)ethanethioate, 22

Following general procedure **B**, 1, 2-(((benzyloxy)carbonyl)(methylamino) acetic acid (5 g, 22.40 mmol) was transformed into its phenyl thioester with isobutyl chloroformate (3.22 mL, 24.64 mmol) thiophenol (5.03 mL, 49.3 mmol) and triethylamine (6.24 mL, 44.8 mmol). After workup the crude was purified by gradient column chromatography (10-30 % EtOAc in hexanes) to yield **22** (6.27 g, 19.88 mmol, 89 % yield) as a colourless oil.

LCMS RT (Method 2) 3.57 min (m/z 316.1)

^{1}H NMR (400 MHz, $CDCl_3$) δ 7.46 – 7.26 (m, 10H), 5.19 (d, J = 6.9 Hz, 2H), 4.29 (s, 1H), 4.20 (s, 1H), 3.09 (s, 3H).

^{13}C NMR (101 MHz, $CDCl_3$) δ 196.04, 195.74, 156.59, 155.76, 136.36, 136.25, 134.70, 134.61, 129.58, 129.55, 129.27, 128.50, 128.11, 128.01, 127.86, 126.52, 77.33, 77.01, 76.70, 67.77, 67.73, 58.65, 58.47, 36.58, 35.81. **HRMS (ESI)** m/z calcd for $[M + H]^+$ 316.1002, found 316.1013.

1-(1H-benzo[d][1,2,3]triazol-1-yl)-2-methylpropan-1-one, 10b

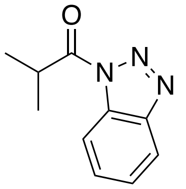

Following general procedure **F**, isobutyric acid (2 g, 88.11 mmol) was transformed into its N-acyl benzotriazole using $SOCl_2$ (1.823 mL, 24.97 mmol) and 1H- benzo[d][1,2,3]triazole (10.82 g, 91 mmol) in DCM (114 mL). The product was purified by gradient column chromatography using 5-10 % EtOAc/hexane to yield **10b** (3.741 g, 19.77 mmol, 87 % yield) as a pale, straw coloured, free flowing oil.

LCMS RT (Method 2) 3.50 min (no m/z in +ve mode)

^{1}H NMR (400 MHz, $CDCl_3$) δ 8.28 (dd, *J* = 8.2, 1.3 Hz, 1H), 8.10 (dd, *J* = 8.2, 1.3 Hz, 1H), 7.63 (td, *J* = 8.3, 7.8, 1.5 Hz, 1H), 7.53 – 7.44 (m, 1H), 4.14 (hept, *J* = 6.9 Hz, 1H), 1.42 (d, *J* = 6.9 Hz, 6H).

^{13}C NMR (101 MHz, $CDCl_3$) δ 176.61, 146.14, 131.28, 130.29, 126.03, 120.06, 114.55, 33.96, 19.07.

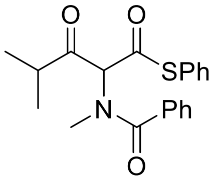

S-phenyl 4-methyl-2-(N-methylbenzamido)-3-oxopentanethioate, 12a

Following general procedure **C**, 1-(1Hbenzo[d][1,2,3]triazol-1-yl)-2-methylpropan-1-one (265 mg, 1.402 mmol) in DCM (6 mL), was coupled to S-phenyl 2-(N-methylbenzamido) ethanethioate (400mg, 1.402 mmol) using $MgBr_2$.diethyl etherate (1.27 g, 4.91 mmol) and diisopropylethylamine (0.979 mL, 5.61 mmol). The crude material was purified by gradient column chromatography to give the title compound (379 mg, 1.065 mmol, 76 % yield) as a colourless oil which solidified upon standing.

LCMS RT (Method 2) 3.64 min (m/z 356.0)

^{1}H NMR (400 MHz, CDCl$_3$) (Mixture of rotamers and tautomers, major enol product reported) δ 12.79 (d, *J* = 1.5 Hz, 1H), 7.69 – 7.57 (m, 2H), 7.46 (s, 5H), 7.43 – 7.30 (m, 3H), 3.37 (s, 3H), 2.54 (heptd, *J* = 6.5, 1.8 Hz, 1H), 1.14 (d, *J* = 6.8 Hz, 3H), 0.69 (d, *J* = 6.7 Hz, 3H).

^{13}C NMR (101 MHz, CDCl$_3$) δ 195.17, 180.01, 172.73, 135.36, 135.19, 135.06, 130.23, 129.89, 129.35, 129.20, 128.53, 127.93, 127.52, 126.87, 126.43, 116.65, 41.35, 38.66, 36.61, 30.26, 29.90, 29.68, 24.66, 20.54, 19.36, 19.23, 17.60.

HRMS (ESI) *m/z* calcd for $[M + H]^+$ 356.1320, found 356.1437.

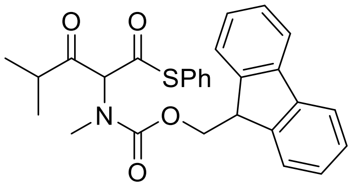

S-phenyl-2-((((9H-fluoren-9-yl)methoxy)carbonyl)(methyl)amino)-4 - methyl-3-oxopentanethioate, 12b

Following general procedure **C** 1-(1H-benzo[d][1,2,3]triazol-1-yl)-2-methylpropan-1-one (1.117 g, 5.90 mmol) was coupled to S-phenyl 2-((((9H-fluoren-9-yl)methoxy)carbonyl)(methyl)amino)ethanethioate (2.5 g, 6.20 mmol) using magnesium bromide diethyl etherate (4.57 g, 17.70 mmol) and diisopropylethylamine in DCM (23 mL). The crude material was purified by gradient column chromatography (0-30 % EtOAc-hexane), giving the title compound (570mg, 1.204 mmol, 20 % yield) as a colourless, mobile oil. *Rotameric, keto enol.*

LCMS RT (Method 2) 4.0 min (m/z 474.7)

^{1}H NMR (400 MHz, CDCl$_3$) δ 12.85 (d, *J* = 1.5 Hz, 1H), 7.81 – 7.71 (m, 2H), 7.65 – 7.49 (m, 2H), 7.46 – 7.30 (m, 7H), 7.28 – 7.21 (m, 2H), 4.51 (dd, *J* = 10.3, 7.4 Hz, 1H), 4.31 (dd, *J* = 10.4, 7.2 Hz, 1H), 4.19 (t, *J* = 7.3 Hz, 1H), 3.16 (s, 3H), 2.67 (sep, *J* = 6.8, 1.5 Hz, 1H), 1.10 (dd, *J* = 6.8 Hz, 6H).

^{13}C NMR (101 MHz, CDCl$_3$) δ 195.09, 179.62, 156.34, 143.69, 143.64, 141.26, 141.23, 135.23, 135.16, 129.67, 129.14, 129.13, 127.73, 127.71, 127.18, 127.09, 126.55, 125.21, 125.19, 125.00, 124.87, 119.91, 114.17, 68.41, 47.50, 47.15, 38.00, 36.61, 29.98, 29.94, 19.38 ,19.04, 18.82.

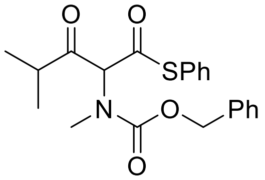

S-phenyl 2-(((benzyloxy)carbonyl)(methyl)amino)-4-methyl-3-oxopentanethioate, 12c

Following general procedure **C**, 1-(1H-benzo[d][1,2,3]triazol-1-yl)-2- methylpropan-1-one (1.143 g, 6.04 mmol was coupled to S-phenyl 2- (((benzyloxy)carbonyl) (methyl)amino)ethanethioate (2 g, 6.34 mmol) using magnesium bromide diethyl etherate (5.46 g, 21.14 mmol)) and diisopropylethylamine (4.22 mL, 24.16 mmol) in DCM (24.16 mL). The crude material was purified by gradient column chromatography (15-30 % EtOAc-hexane) giving the title compound (2.165 g, 5.62 mmol, 93 % yield) as a colourless, mobile oil. *Rotameric, keto enol.*

^{1}H NMR (400 MHz, $CDCl_3$) δ 12.89 – 12.74 (m, 1H), 7.53 – 7.21 (m, 10H), 5.29 – 5.13 (m, 2H), 3.25 – 3.20 (m, 3H), 2.62 (ttd, *J* = 8.3, 6.5, 1.5 Hz, 1H), 1.08 (dd, *J* =6.8, 0.7 Hz, 6H).
^{13}C NMR (101 MHz, $CDCl_3$) δ 195.00, 194.65, 180.38, 179.41, 156.27, 136.34, 135.24, 135.12, 129.63, 129.58, 129.17, 129.13, 128.49, 128.40, 128.12, 128.07, 128.05, 127.90, 126.84, 126.76, 114.04, 67.89, 67.78, 38.36, 38.09, 30.07, 29.85, 19.31, 18.98, 18.69.
HRMS (ESI) *m/z* calcd for $[M + H]^+$ 386.1421, found 386.1427.

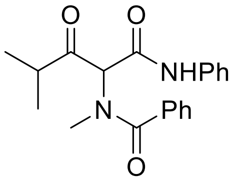

N-methyl-N-(4-methyl-1,3-dioxo-1-(phenylamino)pentan-2-yl)benzamide,13

Following general procedure E, S-phenyl 4-methyl-2-(N-methylbenzamido)-3-oxopentanethioate (100mg, 0.281 mmol) underwent substitution with aniline (31.4 mg, 0.338 mmol) and silver trifluoroacetate (63.4 mg, 0.309 mmol). The crude material was purified by gradient flash chromatography (15-35 % EtOAc/Hexanes) over silica gel affording **13** (89 mg, 0.264 mmol, 94 % yield) as a pale yellow oil. *Rotameric, keto/enol.*

LCMS RT (Method 1) 3.49 min (m/z 339.1)
^{1}H NMR (400 MHz, $CDCl_3$) δ 14.07 (s, 1H), 9.72 (keto, s, 0.3H) 8.41 (s, 1H), 7.63 (d, *J* = 8.6 Hz, 2H), 7.48 – 7.30 (m, 6H), 7.21 (dd, *J* = 12.9, 7.3 14Hz, 2H), 5.55 (keto, s, 0.3H) 3.20 (s, 3H), 3.11 (s, 1H), 2.47 – 2.33 (m, 1H), 1.09 (d, *J* = 6.8 Hz, 3H), 0.50 (d, *J* = 6.7 Hz, 3H).
^{13}C NMR (101 MHz, $CDCl_3$) δ 180.04, 173.32, 168.77, 136.91, 134.49, 130.56, 130.52, 129.07, 129.01, 128.48, 128.04, 127.46, 125.10, 121.31, 119.99, 109.05, 38.07, 29.36, 20.51, 17.64.
HRMS (ESI) *m/z* calcd for $[M + H]^+$ 339.1703, found 339.1712.

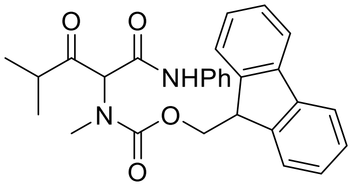

(9H-fluoren-9-yl)methylmethyl(4-methyl-1,3-dioxo-1-(phenylamino) pentan-2-yl)carbamate, 14

Following general procedure **E**, S-phenyl 2-((((9Hfluoren- 9-yl)methoxy)carbonyl) (methyl)amino)-4-methyl-3 oxopentanethioate (370mg, 0.781 mmol) underwent substitution with aniline (109 mg, 1.172 mmol) and silver trifluoroacetate (224 mg, 1.016 mmol) in THF (5.2 mL). The crude material was purified by gradient flash chromatography (15-35 % EtOAc/Hexanes) over silica gel affording **14** (193 mg, 0.422 mmol, 54 % yield). *Rotameric, keto/enol.*

LCMS RT (Method 2) 3.85, 4.01 min (m/z 457.2)

^{1}H NMR (400 MHz, CDCl$_3$) δ 14.23 (s, 1H), 7.80 – 7.70 (m, 1H), 7.69 – 7.60 (m, 2H), 7.56 – 7.45 (m, 2H), 7.45 – 7.40 (m, 1H), 7.39 – 7.32 (m, 3H), 7.31 – 7.22 (m, 2H), 7.22 – 7.10 (m, 2H), 7.06 (t, *J* = 7.5 Hz, 1H), 4.55 – 4.34 (m, 1H), 4.28 (d, *J* = 7.3 Hz, 2H), 3.12 (d, *J* = 29.3 Hz, 3H), 2.68 – 2.53 (m, 1H), 1.17 (d, *J* = 6.7 Hz, 3H), 1.02 (d, *J* = 6.8 Hz, 3H).

^{13}C NMR (101 MHz, CDCl$_3$) δ 180.16, 168.61, 156.94, 143.55, 142.87, 141.11, 136.76, 129.01, 128.96, 127.76, 127.71, 127.12, 127.08, 125.03, 124.88, 124.82, 121.16, 120.18, 119.94, 119.90, 47.14, 46.78, 38.00, 36.63, 29.64, 24.68, 23.37, 19.67, 18.87.

HRMS (ESI) *m/z* calcd for $[M + H]^+$ 457.2122, found 457.2122.

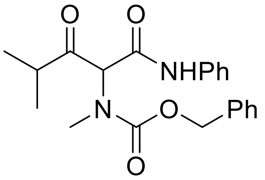

Benzyl methyl(4-methyl-1,3-dioxo-1-(phenylamino)pentan-2-yl)carbamate, 9

Following general procedure **E**, S-phenyl 2- (((benzyloxy)carbonyl) (methyl)amino)-4-methyl-3- oxopentanethioate (370mg, 0.960 mmol) underwent substitution with aniline (134 mg, 1.440 mmol) and silver trifluoroacetate (276 mg, 1.248 mmol) in THF (6.4 mL). The crude material was purified by gradient flash chromatography (15-35 % EtOAc/Hexanes) over silica gel affording **9** (325 mg, 0.883 mmol, 92 % yield). *Rotameric, keto/enol*

LCMS RT (Method 2) 3.61, 3.78 min (m/z 369.2)

^{1}H NMR (400 MHz, CDCl$_3$) δ 14.01 (d, *J* = 1.4 Hz, 1H), 7.70 – 6.96 (m, 11H), 5.31 – 4.98 (m, 2H), 3.13 (s, 3H), 2.55 (sept, *J* = 7.0, 6.4 Hz, 1H), 1.06 (dd, *J* = 66.1, 6.8 Hz, 6H).

^{13}C NMR (101 MHz, CDCl$_3$) δ 13C NMR (101 MHz, cdcl3) δ 179.93, 168.55, 128.97, 128.56, 128.24, 128.11, 124.81, 120.82, 67.87, 38.10, 29.59, 19.58, 18.73.

HRMS (ESI) *m/z* calcd for $[M + H]^+$ 369.1814, found 369.1810.

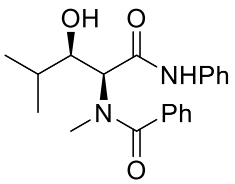

N-((2S,3R)-3-hydroxy-4-methyl-1-oxo-1-(phenylamino)pentan-2-yl)-Nmethyl benzamide, 15

Following general procedure **D**, N-methyl-N-(4-methyl-1,3- dioxo-1-(phenylamino)pentan-2-yl)benzamide (50 mg, 0.147 mmol) underwent ATH DKR to afford **15** (1.9mg, 5.8 µmols, 3.8 % yield) as a white solid.

^{1}H NMR (400 MHz, CDCl$_3$) δ 8.87 (s, 1H), 7.53 (dd, *J* = 15.0, 8.0 Hz, 4H), 7.45 (p, *J* = 6.6 Hz, 3H), 7.34 (t, *J* = 7.8 Hz, 2H), 7.13 (t, *J* = 7.4 Hz, 1H), 5.17 (d, *J* = 4.8 Hz, 1H), 4.06 – 3.99 (m, 1H), 3.16 (s, 3H), 1.96 (dq, *J* = 13.3, 6.6 Hz, 1H), 1.12 (d, *J* = 6.6 Hz, 3H), 0.99 (d, *J* = 6.8 Hz, 3H).

^{13}C NMR (101 MHz, CDCl$_3$) δ 178.65, 174.00, 137.42, 130.87, 129.06, 128.57, 127.88, 124.66, 120.07, 75.61, 74.03, 30.56, 19.34,18.26.

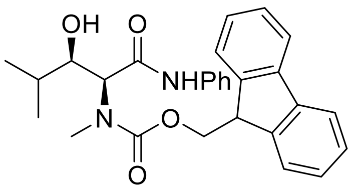

(9H-fluoren-9-yl)methyl((2S,3R)-3-hydroxy-4-methyl-1-oxo-1-(phenyl - amino)pentan-2-yl)(methyl) carbamate, 16

Following general procedure **D**, (9H-fluoren-9-yl)methylmethyl(4-methyl-1,3-dioxo-1-(phenylamino)pentan-2yl)carbamate (50 mg, 0.11 mmols) underwent ATH DKR to yield **16** (9.0mg, 19.8 µmols, 18 % yield) as a white solid.

LCMS RT (Method 2) 3.77 min (m/z 459.2)

^{1}H NMR (400 MHz, CDCl$_3$) δ 8.15 (s, 1H), 7.75 (dd, *J* = 7.6, 4.9 Hz, 2H), 7.60 – 7.52 (m, 2H), 7.52 – 7.44 (m, 2H), 7.41 – 7.25 (m, 5H), 7.21 – 7.06 (m, 2H), 4.67 (d, *J* = 5.5 Hz, 1H), 4.52 (qd, *J* = 10.6, 6.7 Hz, 2H), 4.26 (t, *J* = 6.7 Hz, 1H), 3.93 (d, *J* = 8.5 Hz, 1H), 3.47 (s, 1H), 3.06 (s, 3H), 1.82 – 1.66 (m, 1H), 1.03 (d, *J* = 6.7 Hz, 3H), 0.94 (d, *J* = 6.6 Hz, 3H).

^{13}C NMR (101 MHz, CDCl$_3$) δ 168.94, 158.30, 143.69, 143.44, 141.36, 137.22, 128.99, 127.83, 127.08, 124.82, 120.25, 120.03, 75.17, 68.11, 47.25, 36.61, 30.21, 24.66, 23.33, 19.56, 17.77.

HRMS (ESI) *m/z* calcd for [M + H] 459.2278, found 459.2288.

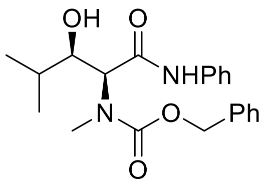

Benzyl((2S,3R)-3-hydroxy-4-methyl-1-oxo-1-(phenylamino)pentan-2-yl)(methyl) carbamate, 17

Following general procedure **D**, benzyl methyl(4-methyl-1,3-dioxo-1 (phenylamino) pentan-2 yl)carbamate **9** (50mg, 0.136 mmol)) underwent ATH DKR with pre-formed [(R,R)- Teth-TsDpen Ru] (7.92 mg, 0.014 mmol) and TBAI (122 mg, 0.330 mmol) in in a DCM/HCO_2Na emulsion. The crude is purified by gradient column chromatography to yield **17** (32.2 mg, 0.087 mmol, 64 % yield) as a white solid.

LCMS RT (Method 2) 3.38 min (m/z 371.1)

^{1}H NMR (400 MHz, $CDCl_3$) δ 8.20 (s, 1H), 7.43 (d, *J* = 7.6 Hz, 2H), 7.40 – 7.26 (m, 7H), 7.14 – 7.09 (m, 1H), 5.27 – 5.14 (m, 2H), 4.79 – 4.70 (m, 1H), 3.99 – 3.91 (m, 1H), 3.56 (d, *J* = 4.4 Hz, 1H), 3.13 (s, 3H), 1.81 (m, *J* = 6.7 Hz, 1H), 0.99 (d, *J* = 6.7 Hz, 6H).

^{13}C NMR (101 MHz, $CDCl_3$) δ 169.10, 158.45, 129.00, 128.60, 128.24, 127.77, 124.71, 120.06, 77.00, 75.21, 68.15, 30.22, 19.49, 17.87)

HRMS (ESI) *m/z* calcd for [M + H] 371.1965, found 371.1963.

(2S, 3R) $[\alpha]_D^{20}$: -118.7 (*c* 1.00, $CHCl_3$)

(2R, 3S) $[\alpha]_D^{20}$: +109.3 (*c* 1.00, $CHCl_3$) [Using (S, S)-catalyst]

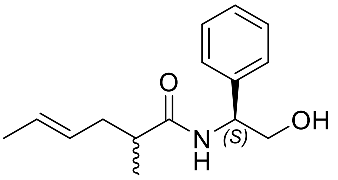

(4E)-N-((S)-2-hydroxy-1- phenylethyl)-2-methylhex-4-enamide, 19/*epi*-19

To a solution of (E)-2-methylhex-4-enoic acid (rac-**20**) (prepared according to Rich et al.[6]) (2.5 g, 19.51 mmol) in DCM was added HATU (8.90 g, 23.41 mmol) and DIPEA (4.09 mL, 23.41 mmol). After allowing 5 minutes for preactivation, (S)-2-amino-2- phenylethanol (3.48 g, 25.4 mmol) was added and the reaction was stirred overnight. The solvent was removed by rotary evaporation, then the crude was dissolved in EtOAc and which was washed with 1N HCl, 1N NaOH and brine. Organics were dried over $MgSO_4$, filtered, evaporated and purified by column chromatography (30 % EtOAc in Hexanes) to yield (E)-N-((S)-2-hydroxy-1- phenylethyl)-2-methylhex-4-enamide (4.36 g, 17.63 mmol, 90 % yield) as a separable mixture of diastereomers.

LCMS RT (Method 2) 2.89 min (m/z 248.1)
HRMS (ESI) m/z calcd for [M + H] 248.1645, found 248.1647.

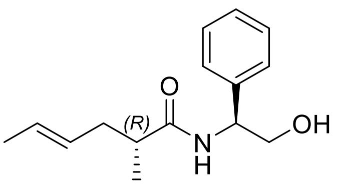

(2R,4E)-N-((S)-2-hydroxy-1-phenylethyl)-2-methylhex-4-enamide, 19

$[\alpha]_D^{20}$ +23.0 (*c* 1.00, $CHCl_3$)
^{1}H NMR (400 MHz, $CDCl_3$) 7.39 – 7.22 (m, 5H), 6.23 (d, J = 7.1 Hz, 1H), 5.51 – 5.38 (m, 1H), 5.32 (dddd, J = 15.1, 6.8, 5.2, 1.5 Hz, 1H), 5.03 (dt, J = 7.1, 4.9 Hz, 1H), 3.84 (td, J = 5.0, 4.4, 1.4 Hz, 2H), 2.96 – 2.88 (m, 1H), 2.35 – 2.20 (m, 2H), 2.15 – 2.01 (m, 1H), 1.59 (dq, J = 6.3, 1.2 Hz, 3H), 1.15 (d, J = 6.4 Hz, 3H).
^{13}C NMR (101 MHz, $CDCl_3$) 176.67, 139.03, 128.82, 128.12, 127.79, 127.73, 126.66, 66.69, 55.72, 41.73, 37.34, 17.90, 17.43.

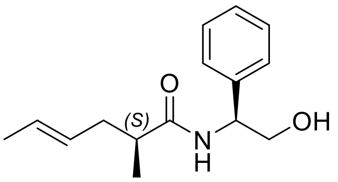

(2S,4E)-N-((S)-2-hydroxy-1-phenylethyl)-2-methylhex-4-enamide, *epi*-19

$[\alpha]_D^{20}$ +41.1 (*c* 1.00, $CHCl_3$)
^{1}H NMR (400 MHz, $CDCl_3$) ä 7.39 – 7.13 (m, 5H), 6.73 (d, J = 7.1 Hz, 1H), 5.51 – 5.27 (m, 2H), 5.01 – 4.86 (m, 1H), 3.71 (d, J = 5.2 Hz, 2H), 3.49 (s, 1H), 2.24 (h, J = 7.1 Hz, 2H), 2.03 (q, J = 10.5, 9.4 Hz, 1H), 1.69 – 1.56 (m, 3H), 1.04 (d, J = 6.6 Hz, 3H)
^{13}C NMR (101 MHz, $CDCl_3$) 176.85, 139.31, 128.64, 128.17, 127.55, 126.62, 66.11, 55.66, 41.33, 37.18, 17.96, 17.28

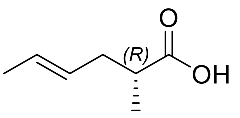

(2R,4E)-2-methylhex-4-enoic acid, 20

A solution of (2R,4E)-N-((S)-2-hydroxy-1-phenylethyl)-2-methylhex-4-enamide **19** (1 g, 4.04 mmol) in 6N H_2SO4 in 60 mL (H_2O/Dioxane) was heated to 80°C for 3 hours, then the reaction was cooled to room temperature. The reaction was diluted with water (50 mL), extracted with DCM and dried with Mg_2SO4. The volatiles were removed under reduced pressure to yield (R,E)-2-methylhex-4-enoic acid, **20** (0.503 g, 3.92 mmol, 97 % yield) as a translucent, yellow oil.

$[\alpha]_D^{20}$ -13.9 (*c* 1.00, $CHCl_3$) (Lit. $[\alpha]_D^{RT}$ -10.2 in Et_2O)
LCMS RT (Method 1) 2.95 min (no m/z in +ve mode)
^{1}H NMR (400 MHz, $CDCl_3$) δ 11.65 (d, *J* = 3.7 Hz, 1H), 5.57 – 5.44 (m, 1H), 5.44 – 5.31 (m, 1H), 2.49 (m, *J* = 6.9 Hz, 1H), 2.43 – 2.30 (m, 1H), 2.13 (dddt, *J* = 12.9, 8.1, 7.1, 1.1 Hz, 1H), 1.65 (dq, *J* = 6.3, 1.2 Hz, 3H), 1.16 (d, *J* = 7.0 Hz, 3H).
^{13}C NMR (101 MHz, $CDCl_3$) δ 182.54, 127.76, 127.49, 39.52, 36.32, 17.88, 16.22.

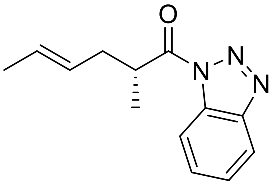

(R,E)-1-(1H-benzo[d][1,2,3]triazol-1-yl)-2-methylhex-4-en-1-one, 21

Following general procedure F. $SOCl_2$ (157 µl, 2.146 mmol) was added to a stirred solution of 1H-benzo[d][1,2,3]triazole (929 mg, 7.80 mmol) in DCM (9 mL), in one portion at room temperature. Stirring was continued for 30 min. (R,E)-2-methylhex-4-enoic acid (250mg, 1.951 mmol) in DCM (1 mL) was added to the reaction mixture, followed by almost immediate precipitation of 1H-benzo[d][1,2,3]triazole.HCl. The reaction mixture was agitated to continue stirring (precipitate forms). Stirring was continued overnight. The white precipitate was filtered off and washed with DCM, then the combined DCM filtrates were evaporated and the crude product was purified by column chromatography (100 % DCM) to yield **21** (422mg, 1.841 mmol, 94 %).

$[\alpha]_D^{20}$ – 64.5 (*c* 1.00, $CHCl_3$)
LCMS RT (Method 1) 3.68 min (m/z 230.1)
^{1}H NMR (400 MHz, $CDCl_3$) δ 8.29 (dt, *J* = 8.3, 1.0 Hz, 1H), 8.11 (dt, *J* = 8.3, 1.0 Hz, 1H), 7.64 (ddd, *J* = 8.3, 7.1, 1.0 Hz, 1H), 7.49 (ddd, *J* = 8.2, 7.1, 1.1 Hz, 1H), 5.57 – 5.37 (m, 2H), 4.13 (h, *J* = 6.9 Hz, 1H), 2.68 – 2.55 (m, 1H), 2.41 – 2.29 (m, 1H), 1.61 – 1.54 (m, 3H), 1.38 (d, *J* = 6.9 Hz, 3H).
^{13}C NMR (101 MHz, $CDCl_3$) δ 175.80, 146.17, 131.21, 130.27, 128.34, 127.00, 126.03, 120.05, 114.57, 39.35, 36.54, 17.85, 16.78.

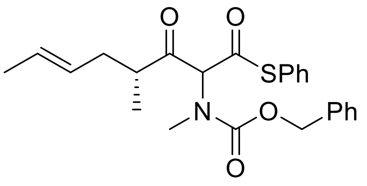

S-phenyl (4R,6E)-2-(((benzyloxy)carbonyl)(methyl)amino)-4-methyl-3-oxooct-6-enethioate, 23

To a stirred solution of (2R,4E)-1-(1H- benzo[d][1,2,3]triazol-1-yl)-2-methylhex-4-en-1-one **21** (385 mg, 1.679 mmol) in anhydrous DCM (6.72 mL) under cooling with an ice bath was added S-phenyl 2- (((benzyloxy)carbonyl)(methyl)amino)ethanethioate **22** (556 mg, 1.763 mmol) followed by magnesium bromide diethyl etherate (1518 mg, 5.88 mmol) and N, N-diisopropylethylamine (1.231 mL, 7.05 mmol). The ice bath was removed and the reaction was allowed to warm to room temperature, monitored by TLC and stirred for 24h. At this point the reaction was re-cooled to 0°C and slowly quenched by the initially dropwise addition of 1N HCl (10 mL). Stirring was continued with cooling for 5 minutes, then the ice bath was removed. The reaction was diluted with brine and EtOAc, the organic phase was separated and the aqueous was again extracted with EtOAc. The combined organic extracts were dried over $MgSO_4$ and evaporated to yield an oil which was purified by gradient column chromatography (0-30 % EtOAc in Hexanes) to yield **23** as a colourless oil (426mg, 1.001 mmol, 60 % yield).

LCMS RT(Method 2) 4.03 min (m/z 426.1)

^{1}H NMR (400 MHz, $CDCl_3$) δ 7.70 – 6.94 (m, 10H), 5.46 (dd, *J* = 13.6, 6.9 Hz, 1H), 5.37 – 5.20 (m, 2H), 5.07 (dd, *J* = 25.8, 12.4 Hz, 1H), 3.18 (d, *J* = 16.7 Hz, 3H), 2.57 – 2.37 (m, 1H), 2.23 (ddt, *J* = 26.6, 12.6, 7.1 Hz, 1H), 2.11 – 1.96 (m, 1H), 1.65 – 1.52 (m, 3H), 1.04 (dd, *J* = 59.9, 7.2 Hz, 3H).

^{13}C NMR (101 MHz, $CDCl_3$) δ 195.05, 194.91, 178.81, 178.66, 156.30, 156.16, 136.35, 136.31, 135.25, 135.14, 129.66, 129.60, 129.18, 129.15, 128.50, 128.41, 128.36, 128.15, 128.08, 128.02, 127.97, 127.87, 127.82, 127.81, 127.58, 127.56, 126.76, 126.72, 115.04, 114.67, 38.60, 38.35, 38.12, 36.66, 36.40, 36.28, 35.59, 17.82, 17.33, 17.00, 16.53.

HRMS (ESI) *m/z* calcd for [M + H] 426.1734, found 426.1744.

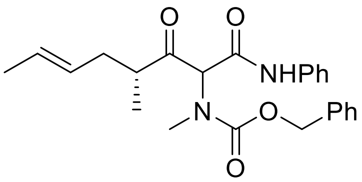

Benzyl methyl((4R, 6E)-4-methyl-1,3-dioxo-1 (phenylamino)oct-6-en-2-yl)carbamate, 24

To a solution of S-phenyl (4R,6E)-2 (((benzyloxy)carbonyl)(methyl)amino)-4-methyl-3-oxooct-6 enethioate **23** (300mg, 0.705 mmol) and aniline (77 µl, 0.846 mmol) in THF (5 mL) at room temperature was added silver trifluoroacetate (187 mg, 0.846 mmol) with stirring. Precipitation of Ag salts requires efficient stirring. Upon completion the reaction was diluted with EtOAc and filtered through Celite. Volatiles were removed by rotary evaporation and the crude material was purified by gradient flash chromatography (5-35 % EtOAc/Hexanes) over silica gel affording **24** (261 mg, 0.641 mmol, 91 % yield). *Rotameric, keto/enol*

^{1}H NMR (400 MHz, $CDCl_3$) δ 14.01 (s, 1H) 7.54 – 7.19 (m, 9H), 7.18 – 7.07 (m, 1H), 5.58 – 5.29 (m, 2H), 5.23 – 4.95 (m, 2H), 3.19 – 3.02 (m, 3H), 2.40 (d, *J* = 6.9 Hz, 1H), 2.35 – 2.22 (m, 1H), 2.12 – 1.98 (m, 1H), 1.63 – 1.56 (m, 3H), 1.04 (dd, *J* = 65.2, 6.8 Hz, 3H).).

^{13}C NMR (101 MHz, $CDCl_3$) δ 179.14, 168.45, 156.12, 128.98, 128.60, 128.56, 128.24, 128.10, 128.01, 127.56, 124.83, 120.83, 68.25, 67.87, 38.32, 36.78, 36.36, 17.92, 17.82.

HRMS (ESI) *m/z* calcd for [M + H] 409.2122, found 409.2131

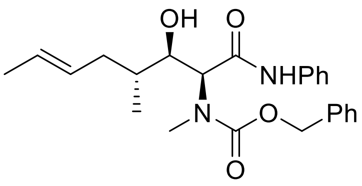

Benzyl ((2S,3R,4R,6E)-3-hydroxy-4-methyl-1-oxo-1 (phenylamino)oct-6-en-2-yl)(methyl)carbamate, 25

To benzyl methyl((4R,E)-4-methyl-1,3-dioxo-1-(phenylamino)oct-6-en-2-yl)carbamate, **24** (26mg, 0.064 mmol) under N_2 was added DCM (0.5 mL), [(R,R)-Teth-TsDpenRu] (3.72 mg, 6.36 µmol) followed by TBAI (47 mg, 0.127 mmol) and 0.5 mL of a 5N solution of HCO_2Na in H_2O. The reaction was sealed and stirred at ~1400 RPM for 48 hours. Following complete consumption of the starting material, the reaction was diluted with 3 mL of sat. $NaHCO_3$, then extracted with 2x3 mL of EtOAc. The organics were combined, dried over $MgSO_4$ and purified by gradient column chromatography to yield **25** (10.3 mg, 0.025 mmol, 61 % yield).

^{1}H NMR (400 MHz, $CDCl_3$) δ 8.18 (s, 1H), 7.65 – 7.15 (m, 9H), 7.16 – 7.08 (m, 1H), 5.46 (qd, *J* = 15.3, 7.1 Hz, 2H), 5.28 – 5.11 (m, 2H), 4.77 (d, *J* = 4.6 Hz, 1H), 4.10 – 3.96 (m, 1H), 3.93 – 3.78 (m, 1H), 3.14 (s, 3H), 2.39 (d, *J* = 13.6 Hz, 1H), 2.01 – 1.89 (m, 1H), 1.71 – 1.60 (m, 4H), 0.88 (d, *J* = 6.7 Hz, 3H).

^{13}C NMR (101 MHz, $CDCl_3$) δ 169.40, 158.45, 137.18, 136.11, 128.99, 128.89, 128.60, 128.23, 127.74, 127.13, 124.72, 120.11, 74.33, 68.16, 60.34, 35.35, 35.06, 33.32, 18.01, 15.82.

HRMS (ESI) *m/z* calcd for [M + H] 411.2278, found 411.2290

(2S, 3R, 4R) $[\alpha]_D^{20}$: -81.8 (*c* 1.00, $CHCl_3$)

(2R, 3S, 4R) $[\alpha]_D^{20}$: +107.6 (*c* 1.00, $CHCl_3$) [Using (S, S)-catalyst]

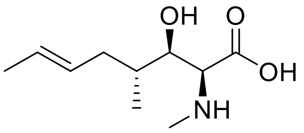

(2S,3R,4R,6E)-3-hydroxy-4-methyl-2-(methylamino)oct-6-enoic acid, MeBmt – 2. Benzyl(E)-(3-hydroxy-4-methyl-1-oxo-1-(phenylamino)oct-6-en-2-yl)(methyl)carbamate **25** (20mg, 0.049 mmol) was added to 0.5 mL of 2N KOH, in a sealed reaction vessel and heated to 80°C by means of an oil bath, followed by overnight stirring. The following day the reaction was cooled to room temperature and diluted with 2 mL of water, then extracted with 2x2 mL Et_2O. The resultant aqueous layer was acidified with DOWEX ion exchange resin, which was washed with water. The amino acid was eluted with 1M aq. NH_3 in H_2O, to yield **1** as an off-white solid (9.2 mg, 0.023 mmol, 46 % yield) whose data were in accordance with previously reported spectra.[5]

LCMS RT (Method 2) 2.24 min (m/z) 202.1.

^{1}H NMR (400 MHz, D_2O) δ 5.40 (qd, *J* = 15.4, 7.0 Hz, 2H), 3.55 (t, *J* = 5.6 Hz, 1H), 3.43 (s, 1H), 3.31 (d, *J* = 5.5 Hz, 1H), 2.56 (s, 2H), 2.45 (s, 3H), 2.15 (d, *J* = 14.0 Hz, 1H), 1.78 – 1.69 (m, 1H), 1.57 (tt, *J* = 6.3, 3.2 Hz, 1H), 1.52 (d, *J* = 5.9 Hz, 3H), 0.78 (d, *J* = 6.8 Hz, 3H).

HRMS (ESI) *m/z* calcd for $[M + H]^+$ 202.1438, found 202.1441.

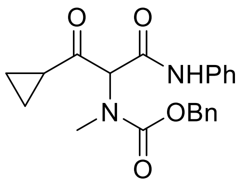

Benzyl (1-cyclopropyl-1,3-dioxo-3-(phenylamino)propan-2-yl)(methyl)carbamate Following general procedure **E**, *S*-phenyl 2-(((benzyloxy)carbonyl)(methyl)amino)-3-cyclopropyl-3-oxopropanethioate (107mg, 0.281 mmol) underwent substitution with aniline (31.4 mg, 0.338 mmol) and silver trifluoroacetate (63.4 mg, 0.309 mmol). The crude material was purified by gradient flash chromatography (15-35 % EtOAc/Hexanes) over silica gel affording the title compound (86.5 mg, 0.236 mmol, 84 % yield) as a clear colourless oil. *Rotameric, keto/enol.*

LCMS RT (Method 2) 3.65min (m/z 367.2)

^{1}H NMR (400 MHz, $CDCl_3$) δ 14.13 (s, 1H), 9.48 (d, *J* = 65.8 Hz, 1H), 7.59 – 7.18 (m, 9H), 7.16 – 7.07 (m, 1H), 5.28 – 5.03 (m, 2H), 3.16 (d, *J* = 26.7 Hz, 3H), 1.52 – 0.61 (m, 5H).

^{13}C NMR (101 MHz, $CDCl_3$) δ 189.08, 168.19, 136.76, 136.40, 128.99, 128.95, 128.51, 128.23, 127.94, 124.66, 124.54, 120.71, 120.09, 67.76, 37.70, 11.22, 7.96, 7.61.

HRMS (ESI) *m/z* calcd for [M + H] 367.1652, found 367.1652.

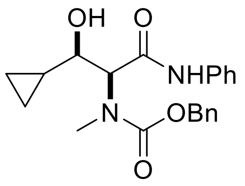

***syn*-benzyl-(1-cyclopropyl-1-hydroxy-3-oxo-3-(phenylamino)propan-2-yl)(methyl)carbamate, 28/29**

Following general procedure **D**, benzyl (1-cyclopropyl-1,3-dioxo-3-(phenylamino)propan-2-yl)(methyl)carbamate (49.7mg, 0.136 mmol)) underwent ATH DKR with pre-formed [(R,R)- Teth-TsDpen Ru] (7.92 mg, 0.014 mmol) and TBAI (122 mg, 0.330 mmol) in in a DCM/HCO_2Na emulsion. The crude is purified by gradient column chromatography to yield the title compound (39.1 mg, 0.106 mmol, 78 % yield) as a white solid.

LCMS RT (Method 2) 3.11 min (m/z 369.1).

^{1}H NMR (400 MHz, $CDCl_3$) δ 8.28 (s, 1H), 7.56 – 7.25 (m, 9H), 7.17 – 7.08 (m, 1H), 5.19 (s, 2H), 4.71 (d, *J* = 6.5 Hz, 1H), 3.80 (s, 1H), 3.59 (d, *J* = 14.2 Hz, 2H), 3.01 (s, 3H), 1.05 – 0.97 (m, 1H), 0.48 (d, *J* = 31.3 Hz, 3H), 0.32 (s, 1H).

^{13}C NMR (101 MHz, $CDCl_3$) δ 178.43, 169.22, 129.02, 128.59, 128.26, 127.90, 124.77, 120.21, 73.88, 68.01, 64.26, 32.80, 14.81, 2.61, 1.80.

HRMS (ESI) *m/z* calcd 369.1809 for [M + H], found 369.1802.

28: (2S, 3R) $[\alpha]_D^{20}$: -23.8 (*c* 1.00, $CHCl_3$)

29: (2R, 3S) $[\alpha]_D^{20}$: +19.2 (*c* 1.00, $CHCl_3$) [Using (S, S)-catalyst]

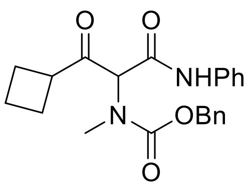

Benzyl (1-cyclobutyl-1,3-dioxo-3-(phenylamino)propan-2-yl)(methyl)carbamate

Following general procedure **E**, *S*-phenyl 2-(((benzyloxy)carbonyl)(methyl)amino)-3-cyclobutyl-3-oxopropanethioate (111mg, 0.281 mmol) underwent substitution with aniline (31.4 mg, 0.338 mmol) and silver trifluoroacetate (63.4 mg, 0.309 mmol). The crude material was purified by gradient flash chromatography (15-35 % EtOAc/Hexanes) over silica gel affording the title compopund (82 mg, 0.224 mmol, 80 % yield) as a clear colourless oil. *Rotameric, keto/enol.*

LCMS RT (Method 2) 3.65min (m/z 381.1)

^{1}H NMR (400 MHz, $CDCl_3$) δ 14.10 (s, 1H), 9.31 (s, 1H), 7.57 – 7.09 (m, 10H), 5.12 (m, 2H), 3.09 (s, 3H), 2.47 – 1.70 (m, 7H).

^{13}C NMR (101 MHz, $CDCl_3$) δ 176.41, 168.40, 136.67, 128.99, 128.55, 128.27, 128.18, 127.92, 124.79, 120.80, 120.09, 67.71, 43.33, 38.03, 35.33, 25.32, 18.28.

HRMS (ESI) *m/z* calcd for [M + H] 381.1809, found 381.1815.

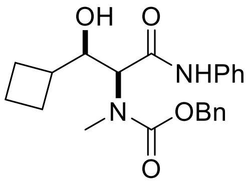

***syn*-benzyl(-1-cyclobutyl-1-hydroxy-3-oxo-3-(phenylamino)propan-2-yl)(methyl) carbamate, 30/31**

Following general procedure **D**, benzyl (1-cyclobutyl-1,3-dioxo-3-(phenylamino)propan-2-yl)(methyl)carbamate (50.3 mg, 0.136 mmol)) underwent ATH DKR with pre-formed [(R,R)- Teth-TsDpen Ru] (7.92 mg, 0.014 mmol) and TBAI (122 mg, 0.330 mmol) in in a DCM/HCO_2Na emulsion. The crude was purified by gradient column chromatography to yield the title compound (27 mg, 0.073 mmol, 54 % yield) as a white solid.

LCMS RT (Method 2) 3.07 min (m/z 383.2).

^{1}H NMR (400 MHz, $CDCl_3$) δ 8.14 (s, 1H), 7.71 – 6.95 (m, 10H), 5.18 (s, 2H), 4.48 (s, 1H), 4.04 (s, 1H), 3.55 (s, 1H), 2.97 (s, 3H), 2.50 (s, 1H), 2.17 – 1.62 (m, 6H).

^{13}C NMR (101 MHz, $CDCl_3$) δ 177.83, 169.32, 129.01, 128.63, 128.32, 127.92, 120.16, 71.98, 68.11, 65.95, 34.76, 23.95, 23.23, 18.04.

HRMS (ESI) *m/z* calcd 383.1965 for [M + H], found 383.1967.

30: (2S, 3R) $[\alpha]_D^{20}$: +53.2 (*c* 1.00, $CHCl_3$)

31: (2R, 3S) $[\alpha]_D^{20}$: -60.2 (*c* 1.00, $CHCl_3$) [Using (S, S)-catalyst]

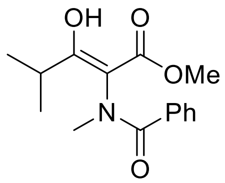

Methyl-3-hydroxy-4-methyl-2-(N-methylbenzamido)pent-2-enoate, 6a

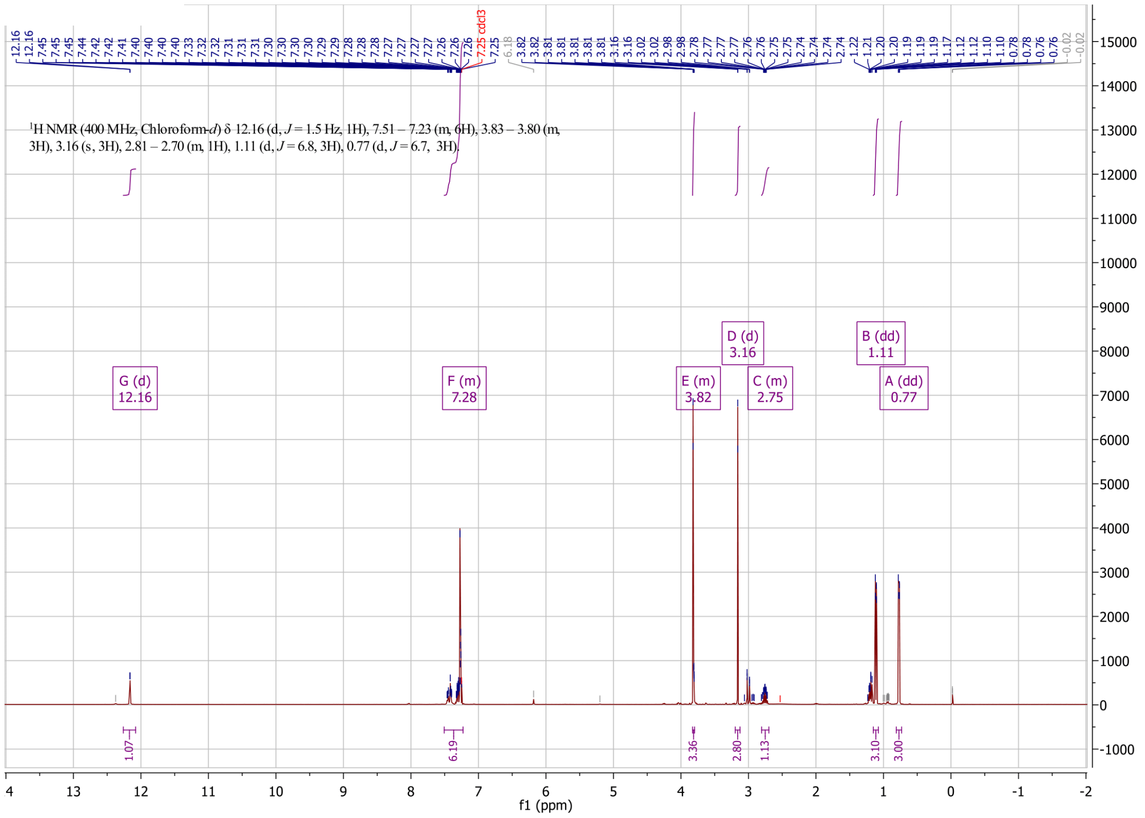

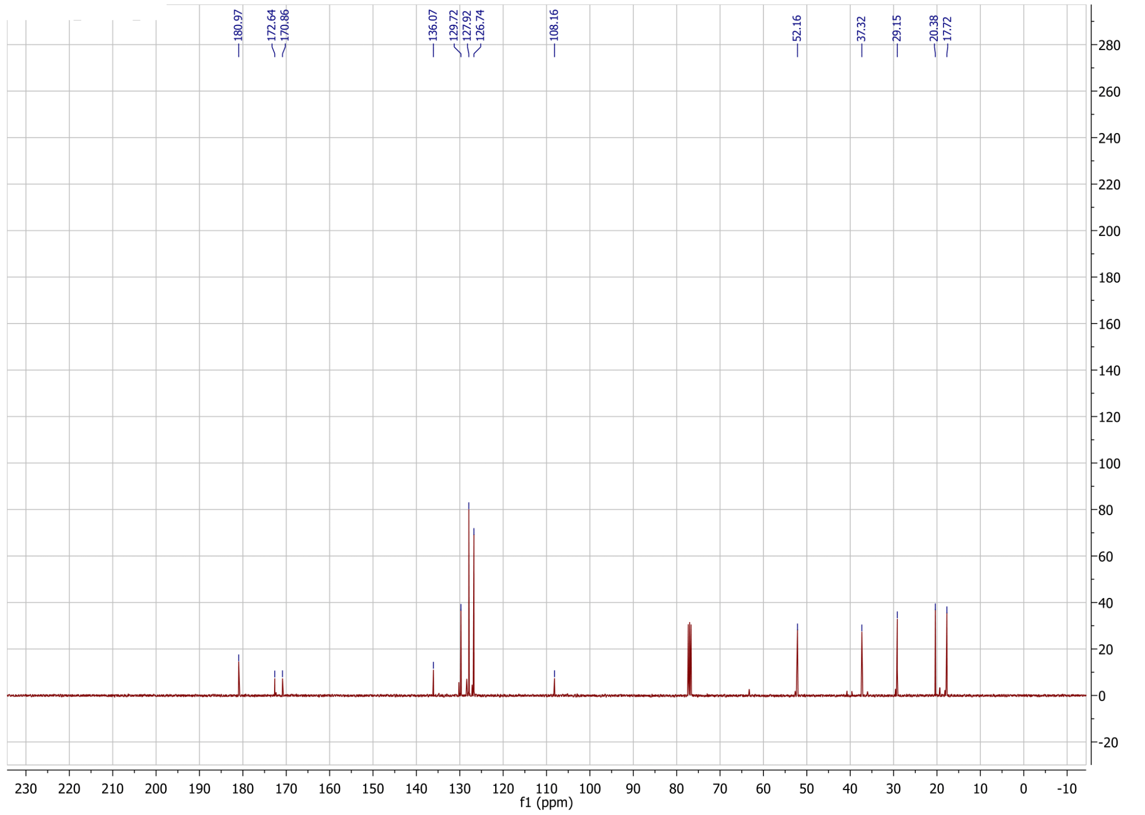

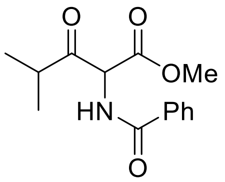

Methyl 2-benzamido-4-methyl-3-oxopentanoate, 6b

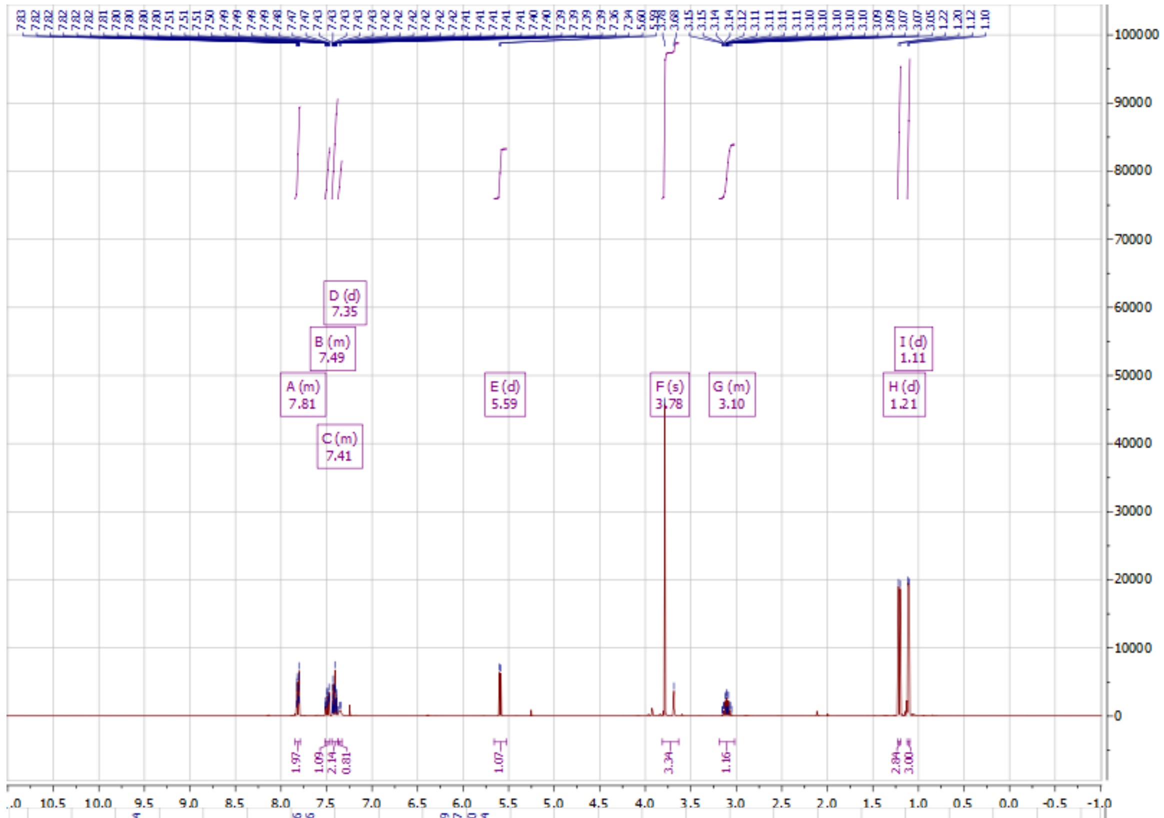

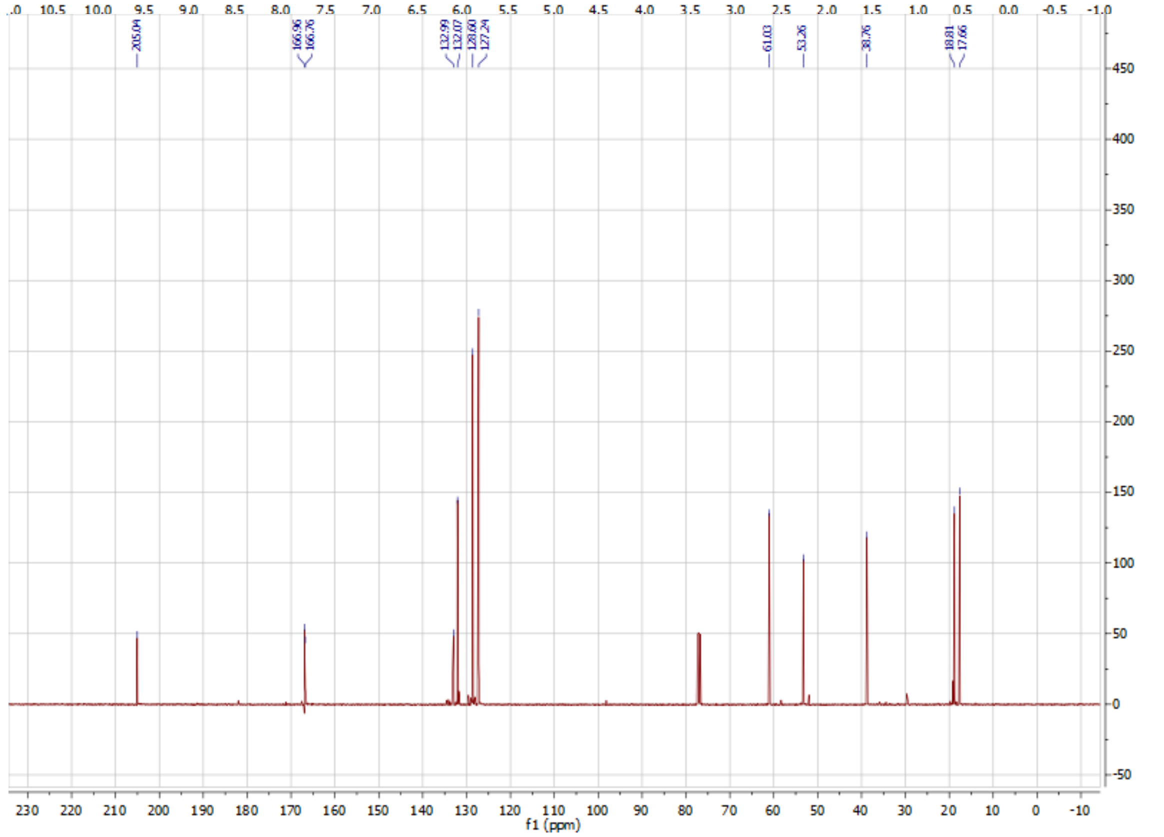

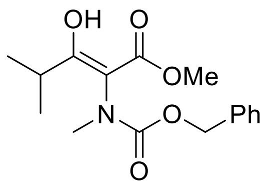

Methyl-2-(((benzyloxy)carbonyl)(methyl)amino)-3-hydroxy-4-methylpent-2-enoate, 6c

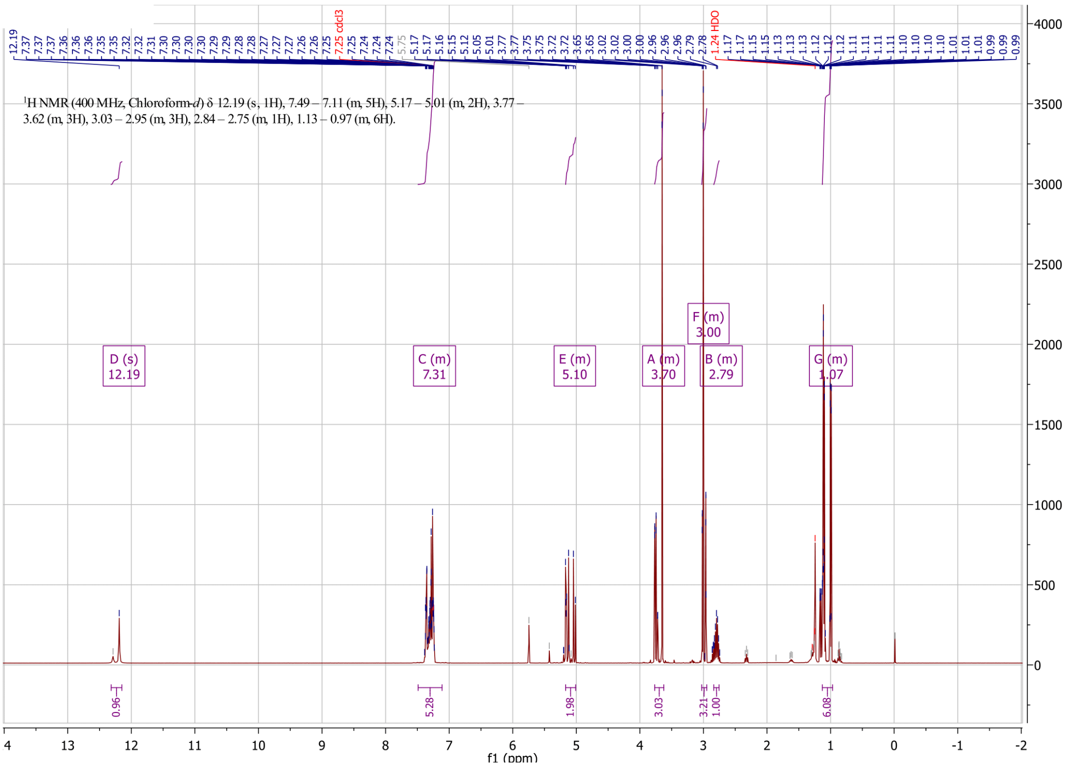

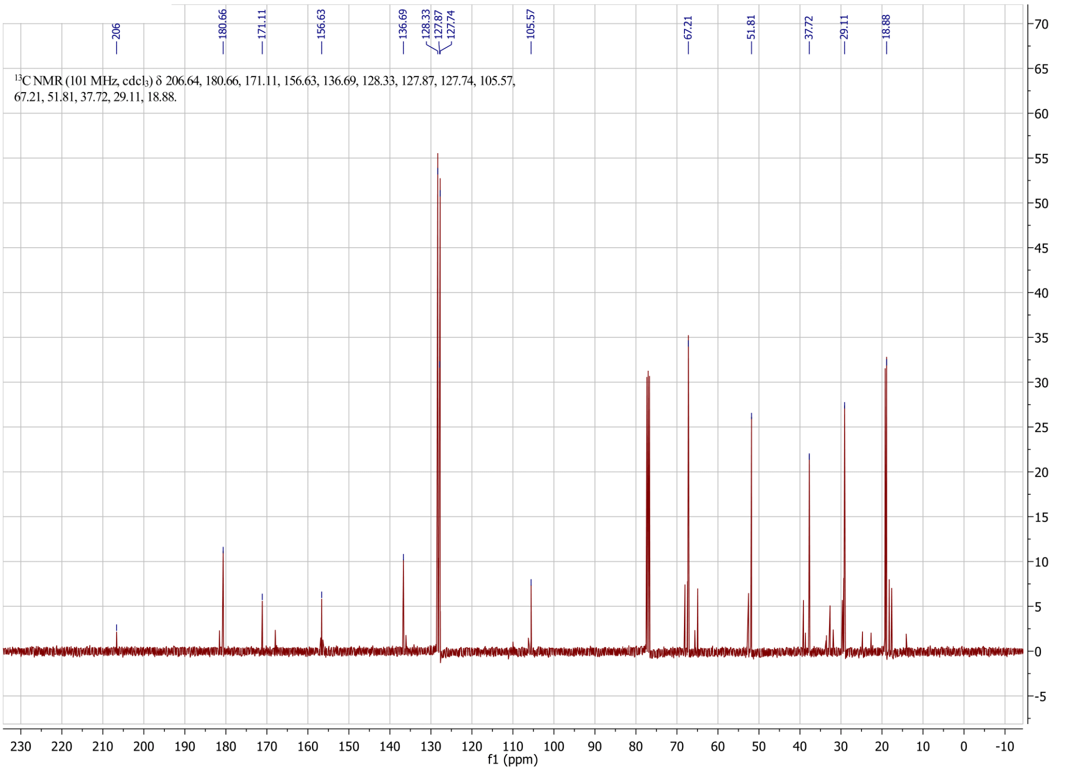

Methyl (2S,3S)-2-benzamido-3-hydroxy-4-methylpentanoate, 8b

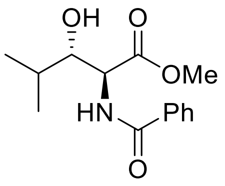

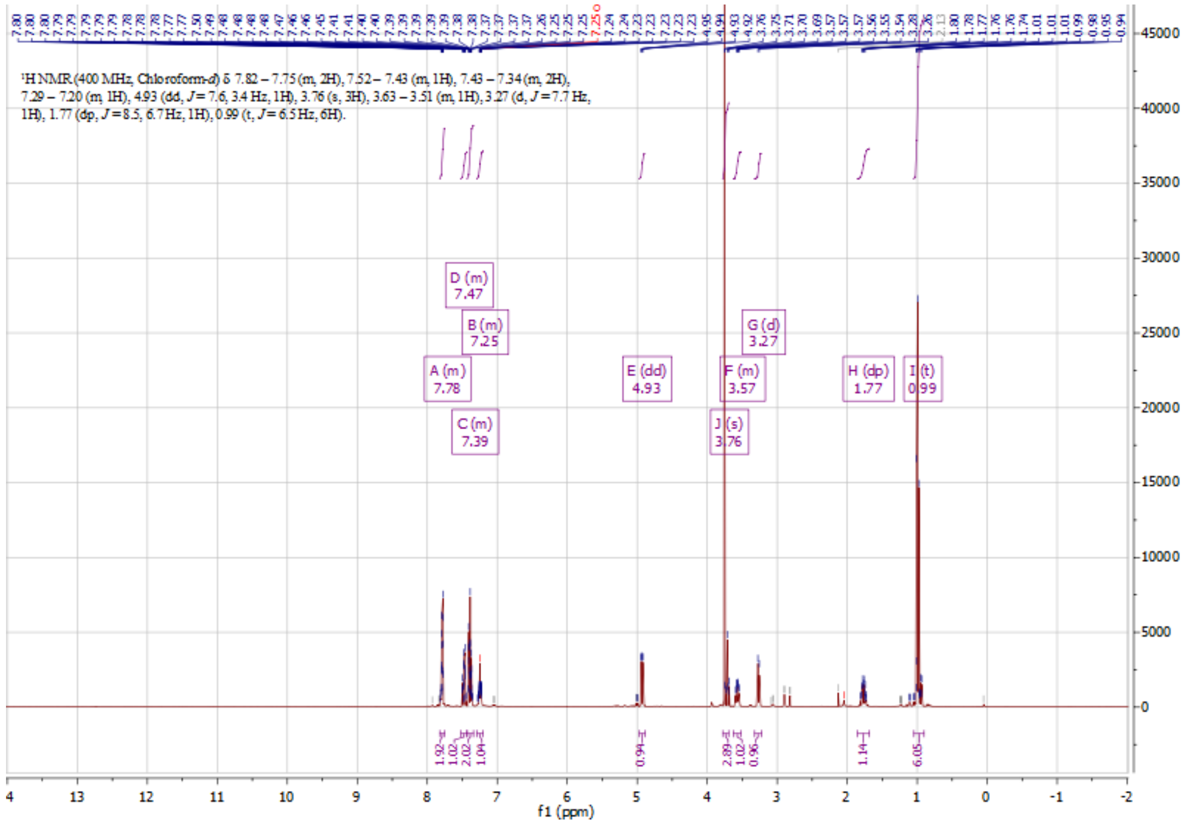

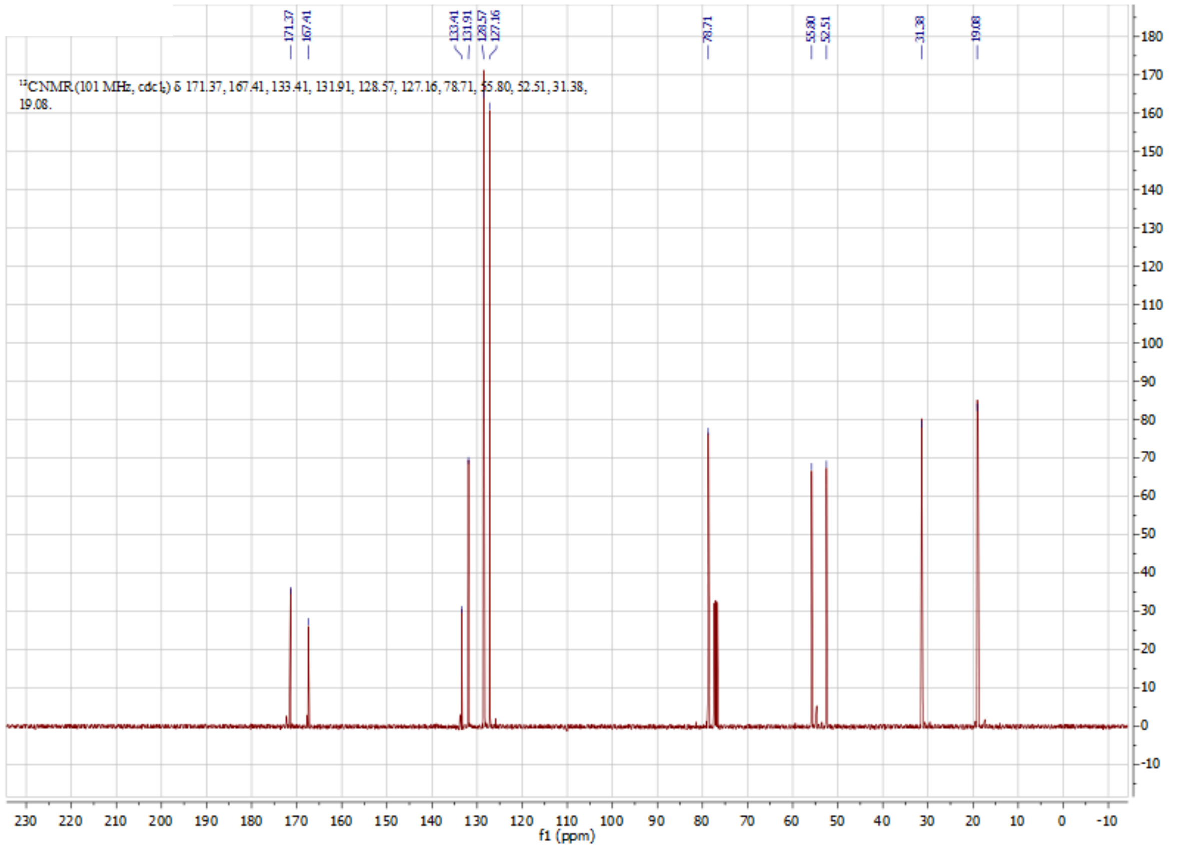

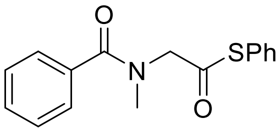

S-phenyl 2-(N-methylbenzamido)ethanethioate

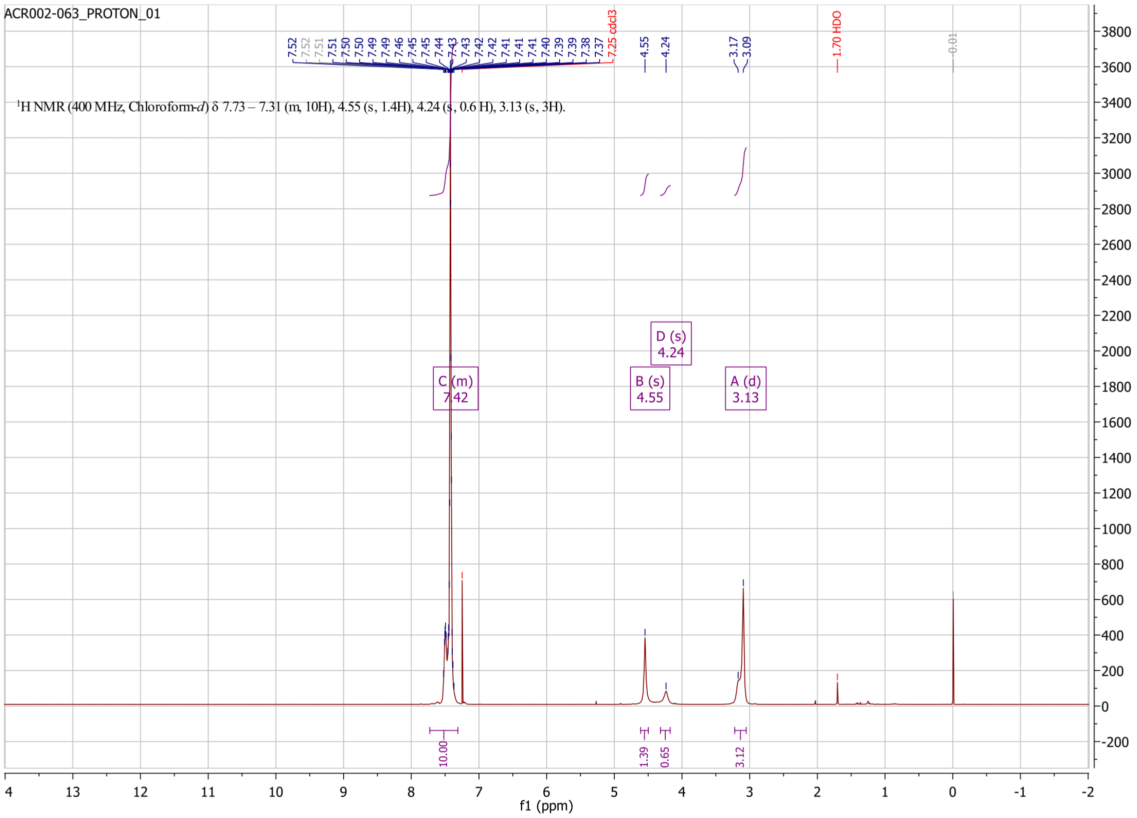

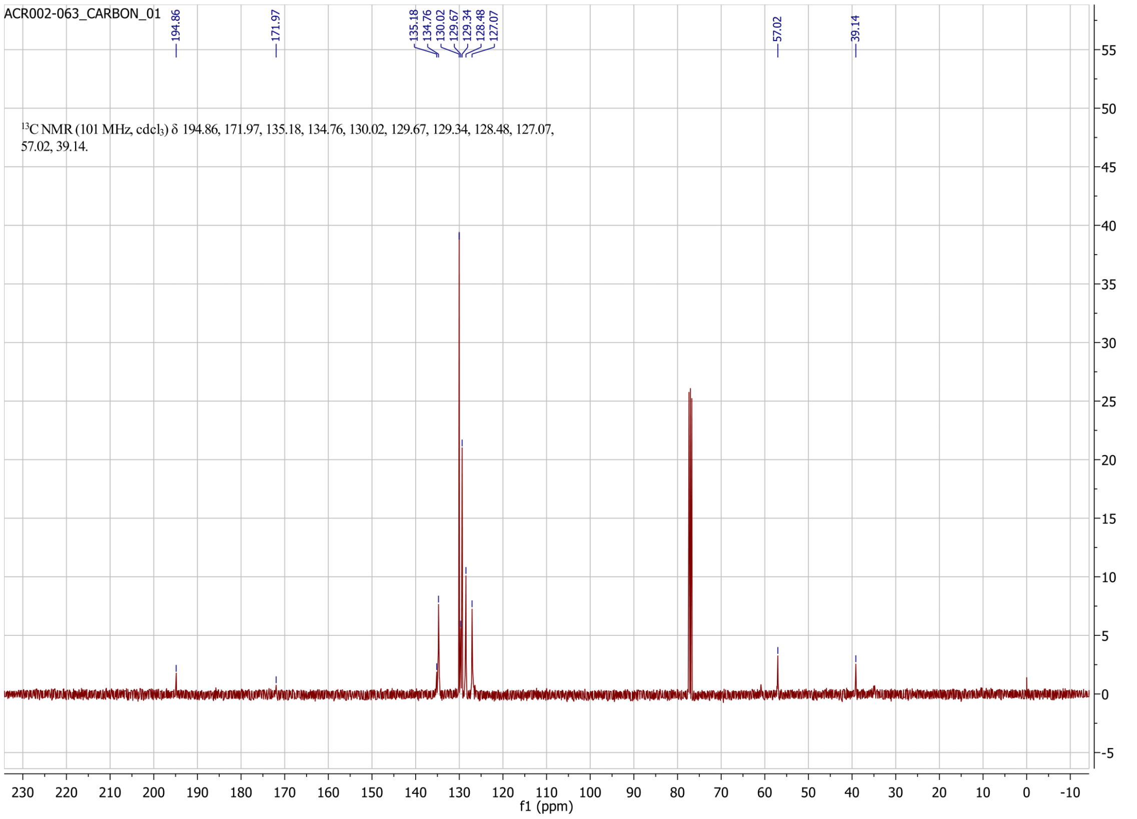

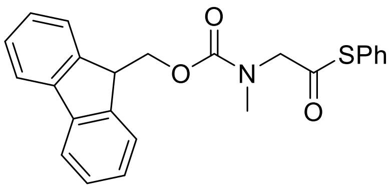

S-phenyl 2-((((9H-fluoren-9yl)methoxy)carbonyl)(methyl)amino) ethane thioate

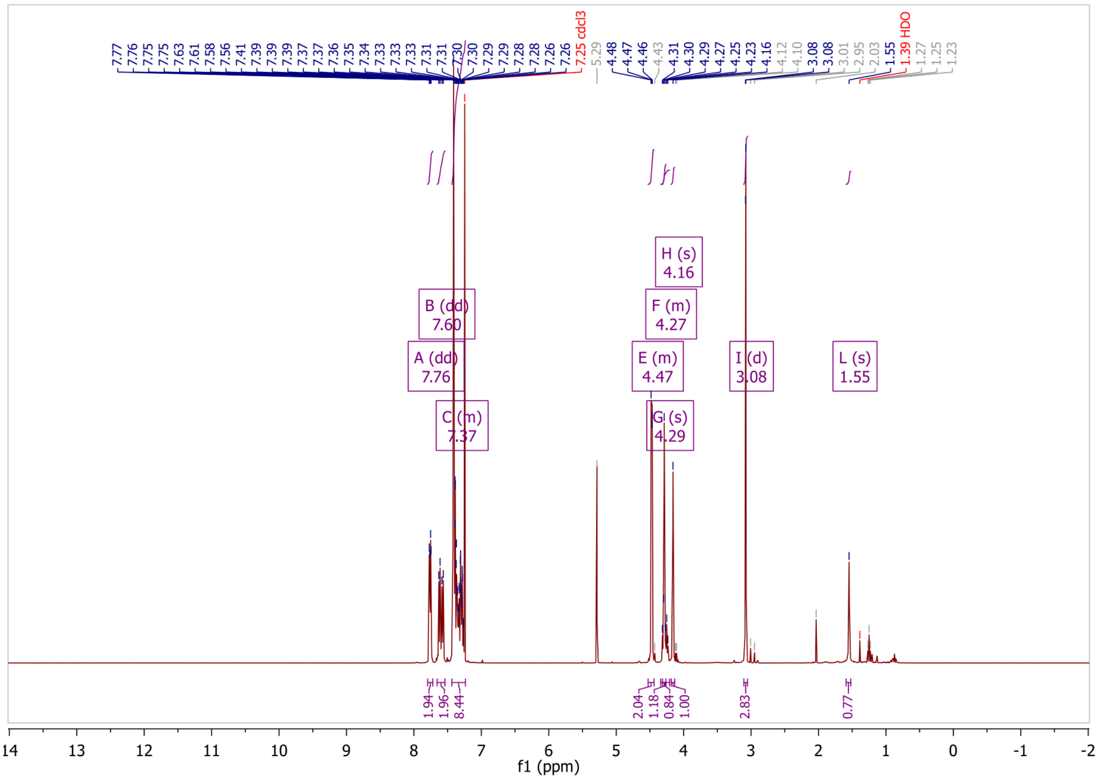

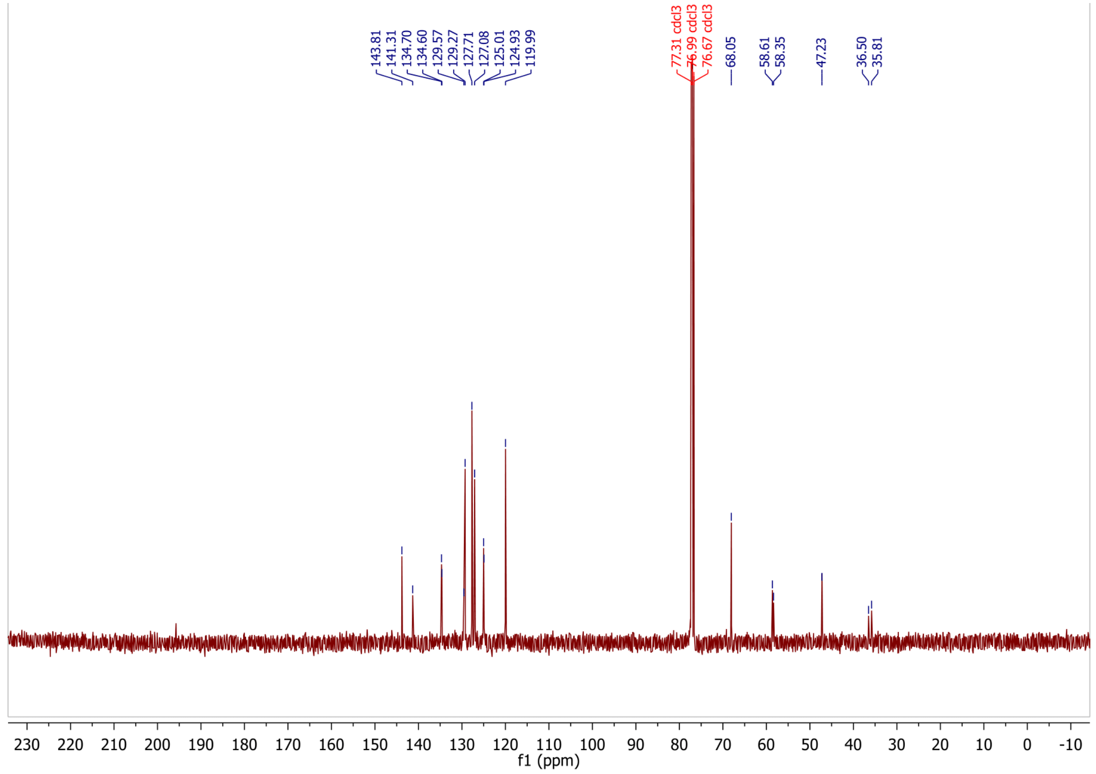

^{13}C NMR (101 MHz, cdcl$_3$) δ 143.81, 141.31, 134.70, 134.60, 129.57, 129.27, 127.71, 127.08, 125.01, 124.93, 119.99, 77.31, 76.99, 76.67, 68.05, 58.61, 58.35, 47.23, 36.50, 35.81.

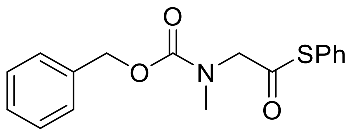

S-phenyl 2- (((benzyloxy)carbonyl)(methyl)amino)ethanethioate, 22

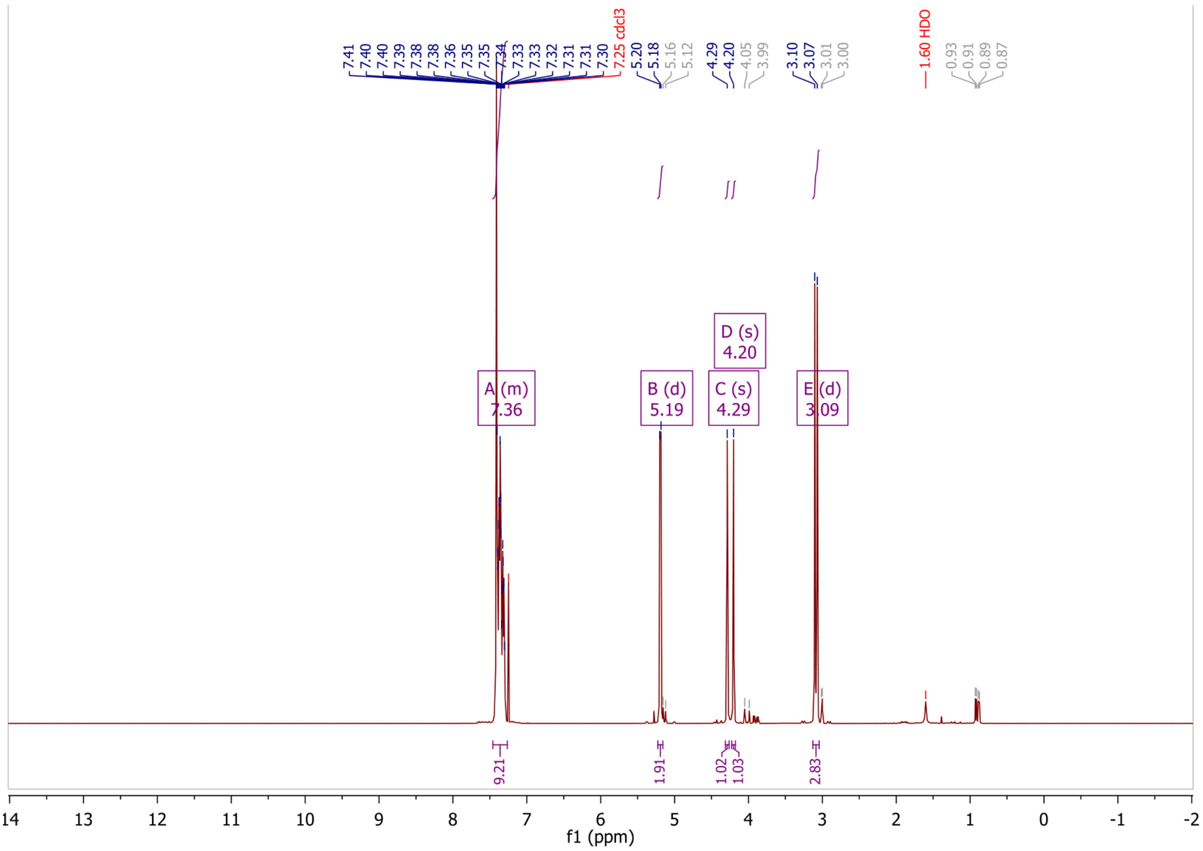

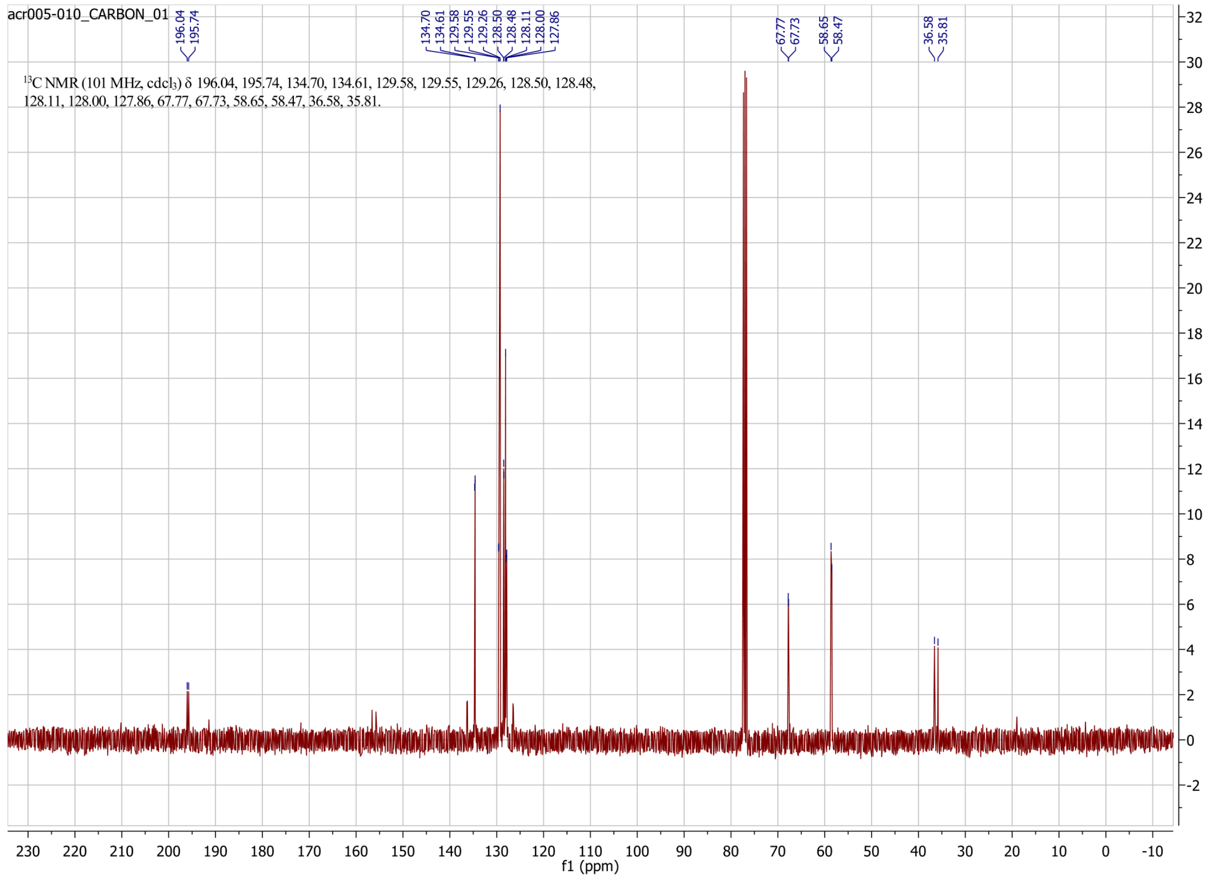

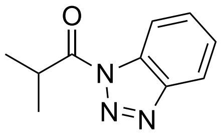

1-(1*H*-benzo[*d*][1,2,3]triazol-1-yl)-2-methylpropan-1-one, 10b

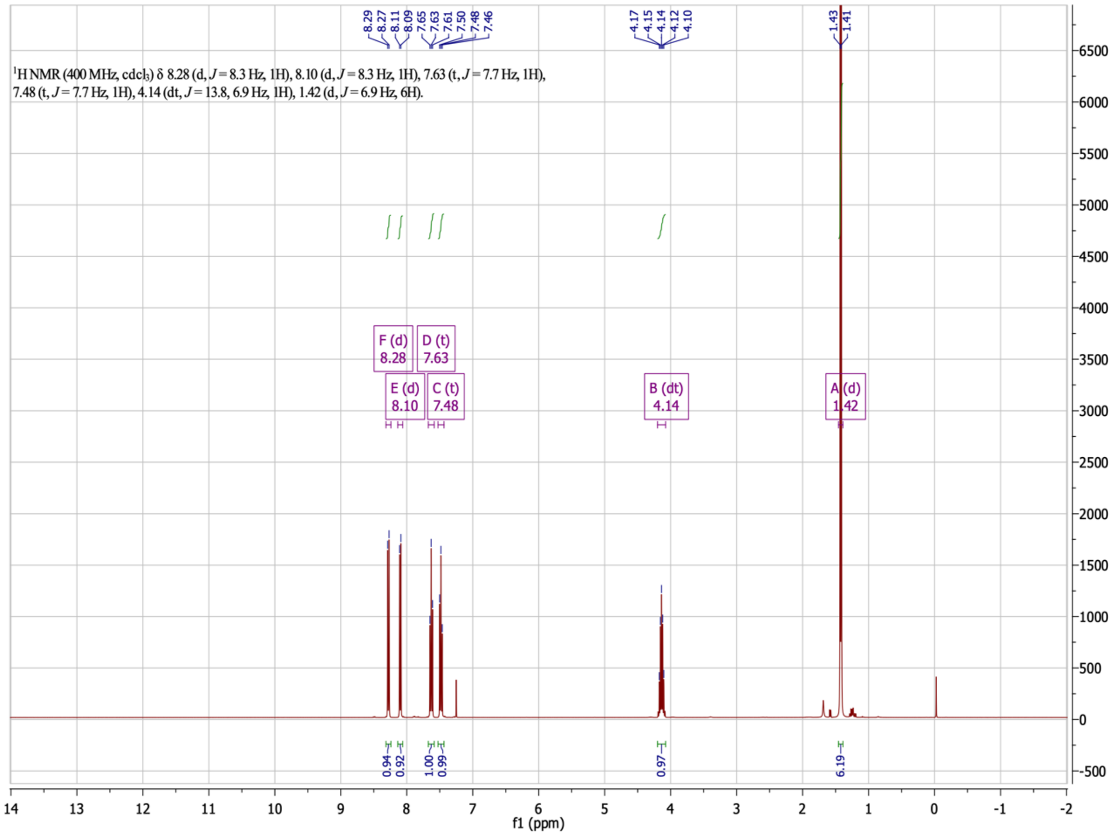

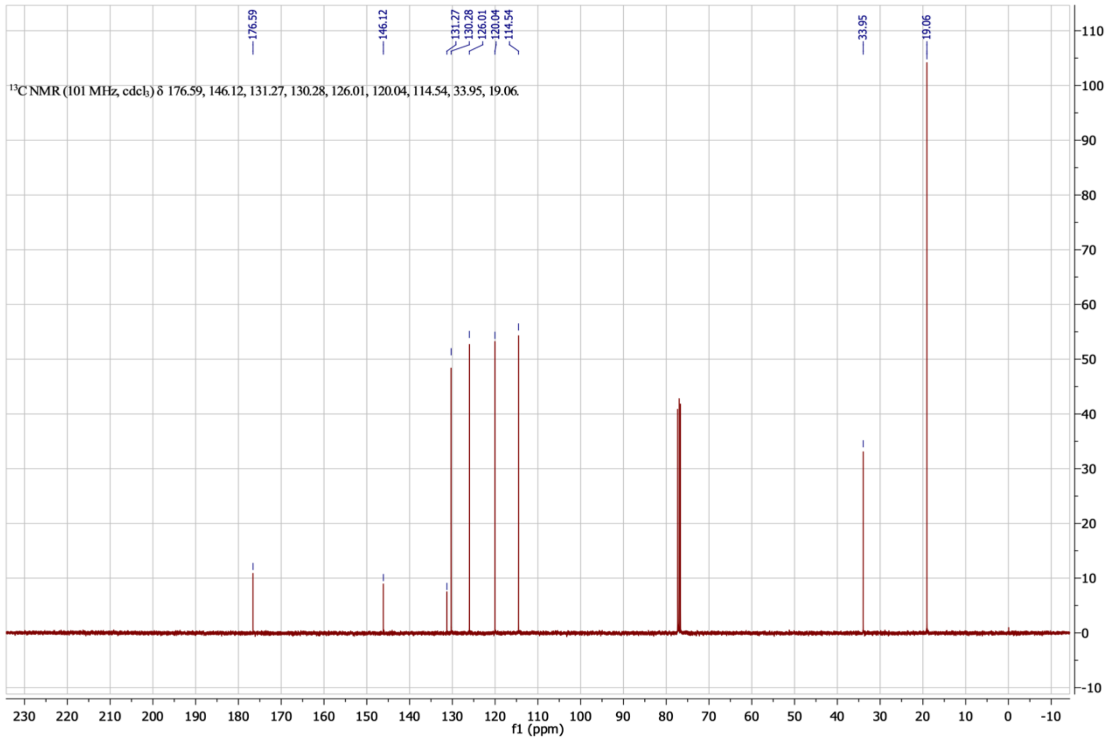

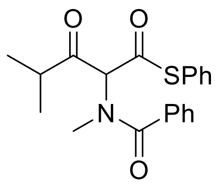

S-phenyl 4-methyl-2-(N-methylbenzamido)-3-oxopentanethioate

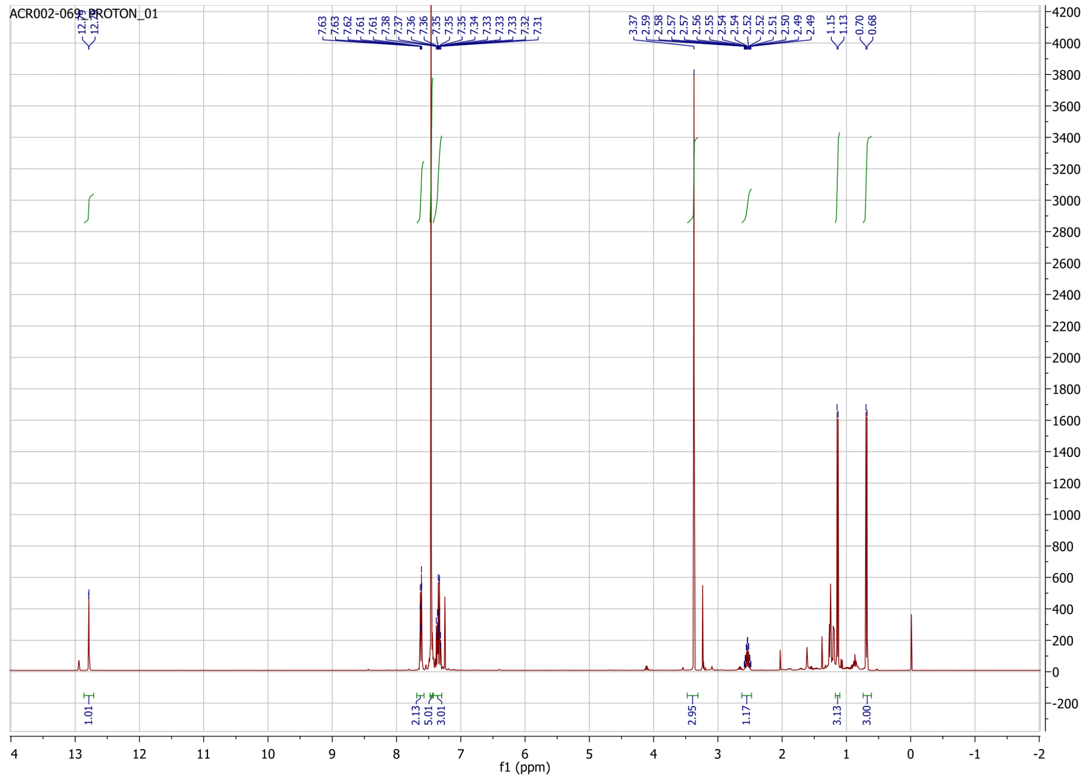

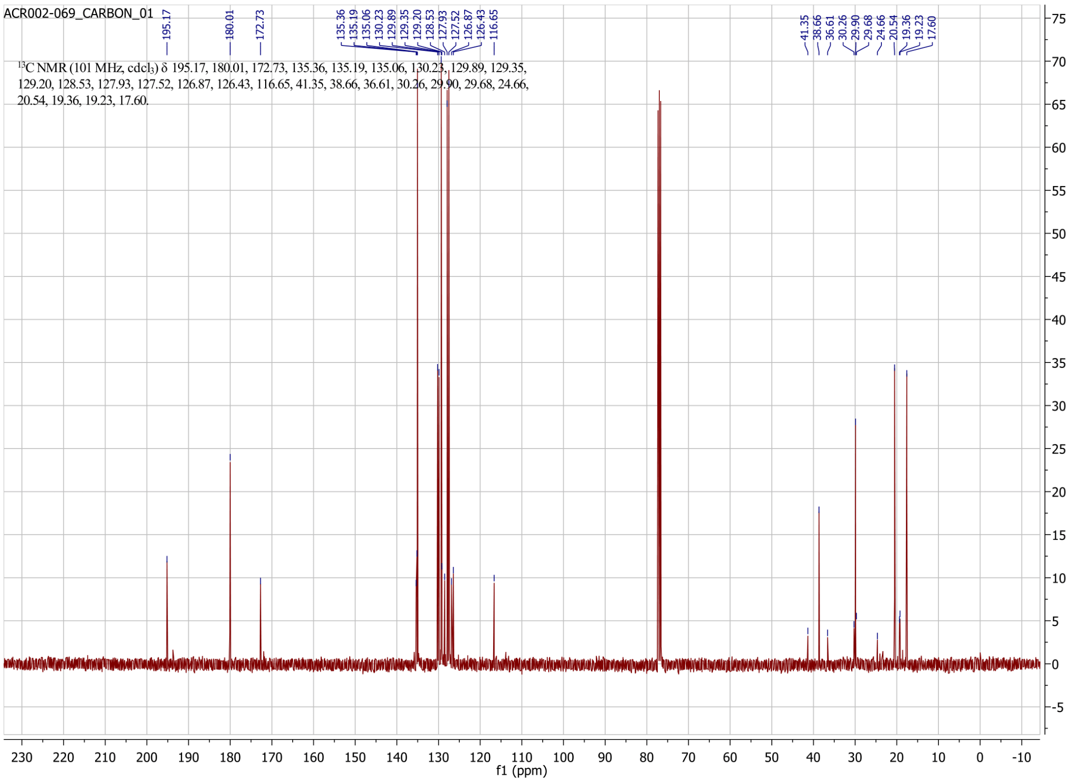

S-phenyl 2-((((9H-fluoren-9-yl)methoxy)carbonyl)(methyl)amino)-4-methyl-3-oxopentanethioate

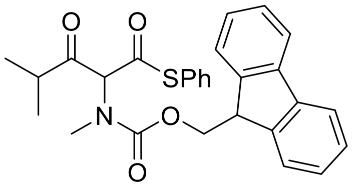

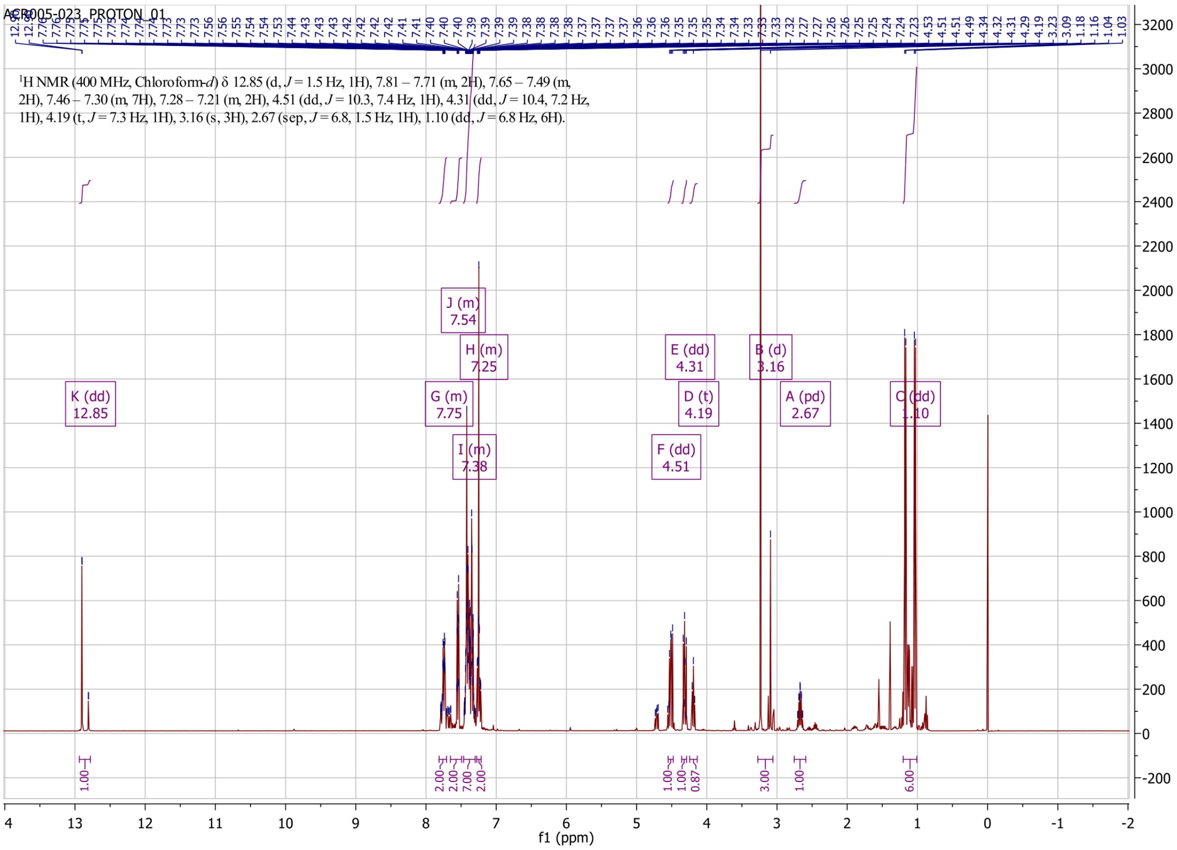

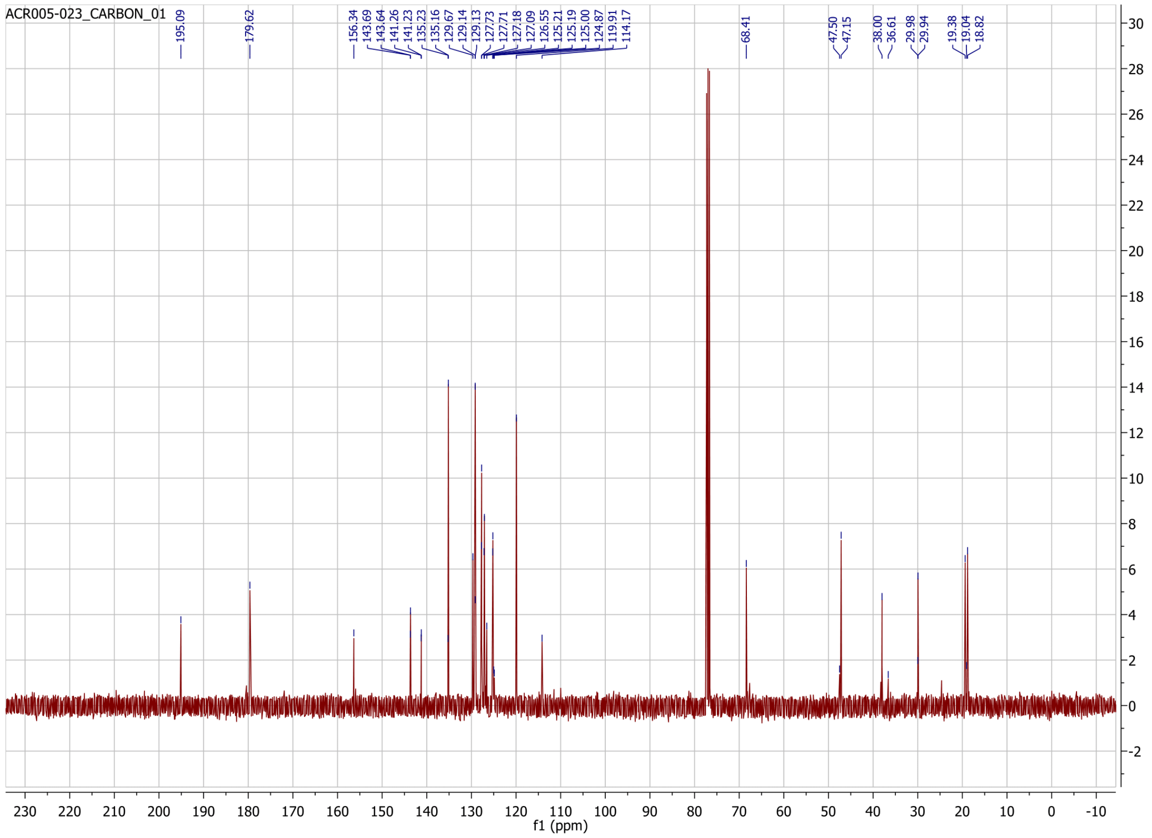

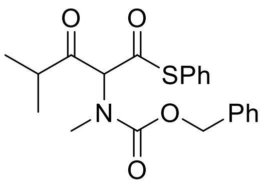

S-phenyl-2(((benzyloxy)carbonyl)(methyl)amino)-4-methyl-3-Oxopentanethioate

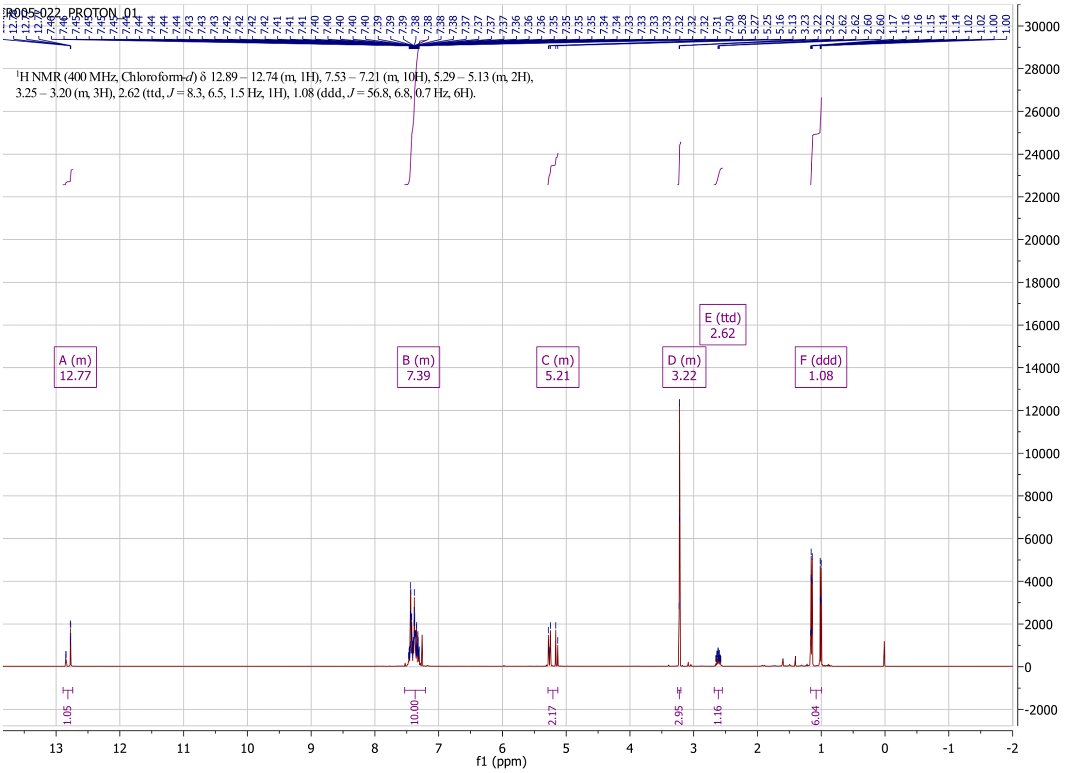

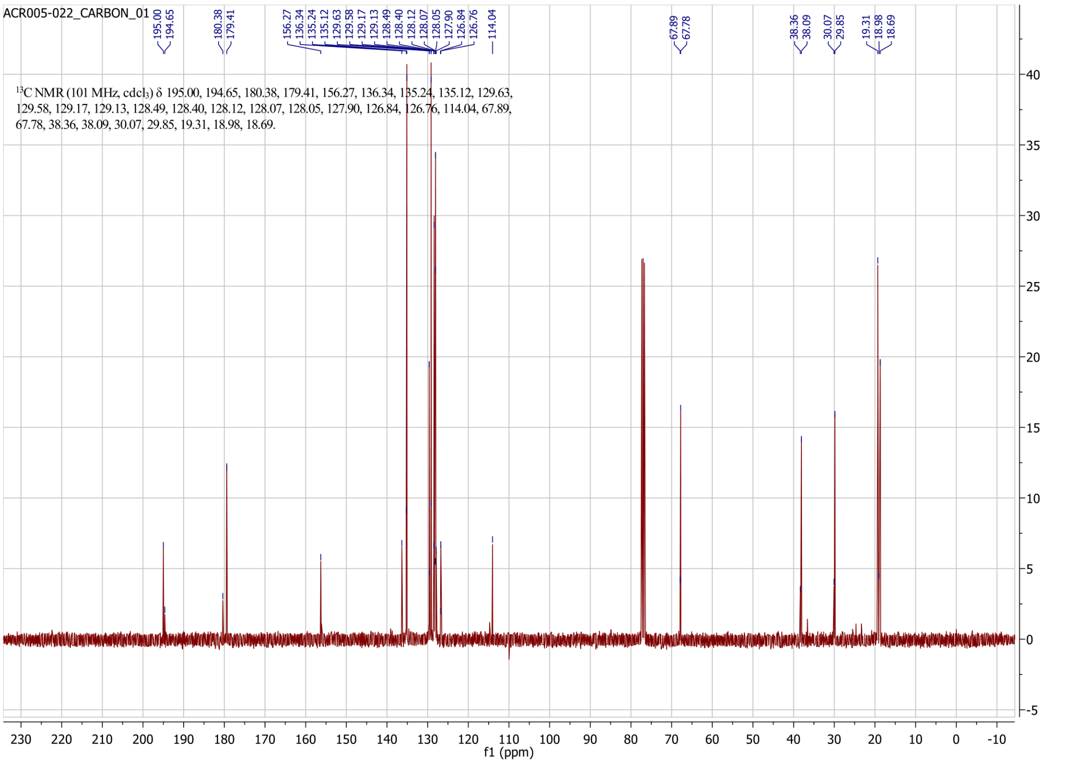

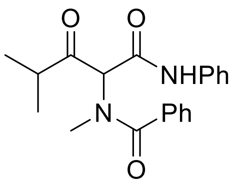

N-methyl-N-(4-methyl-1,3-dioxo-1-(phenylamino)pentan-2-yl)benzamide, 13

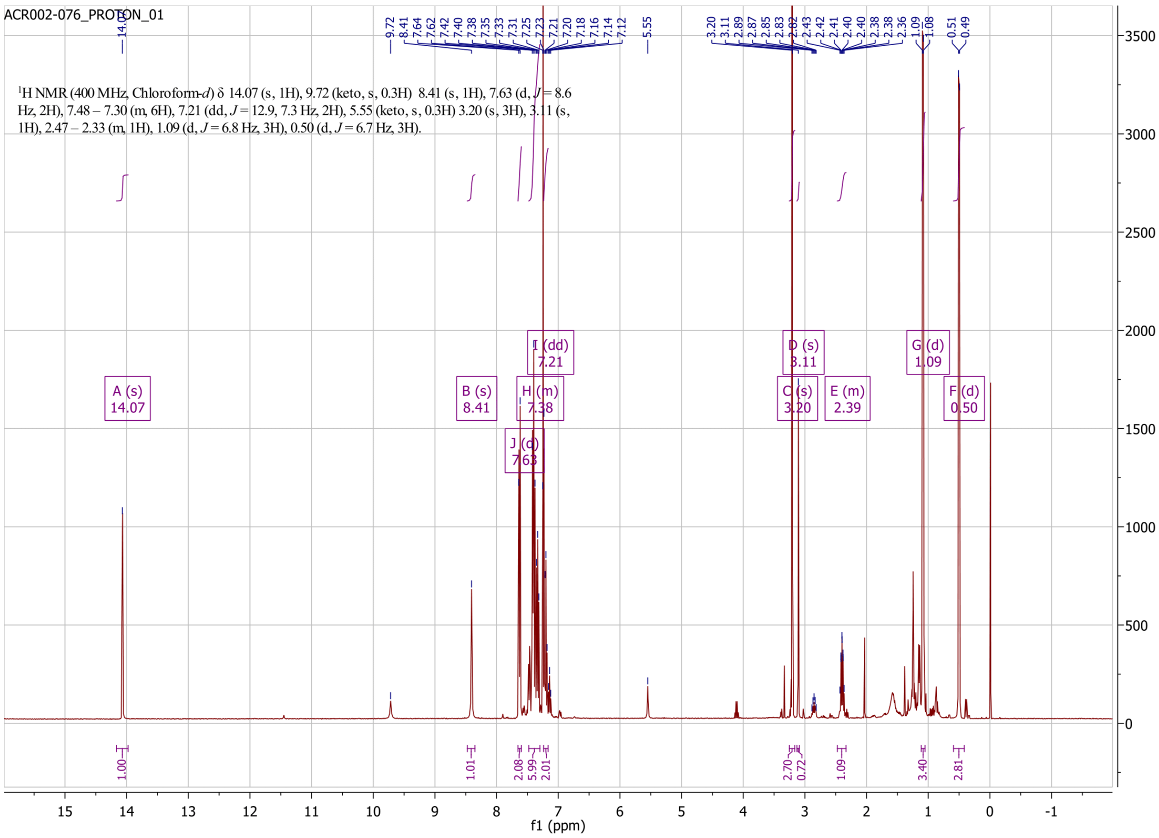

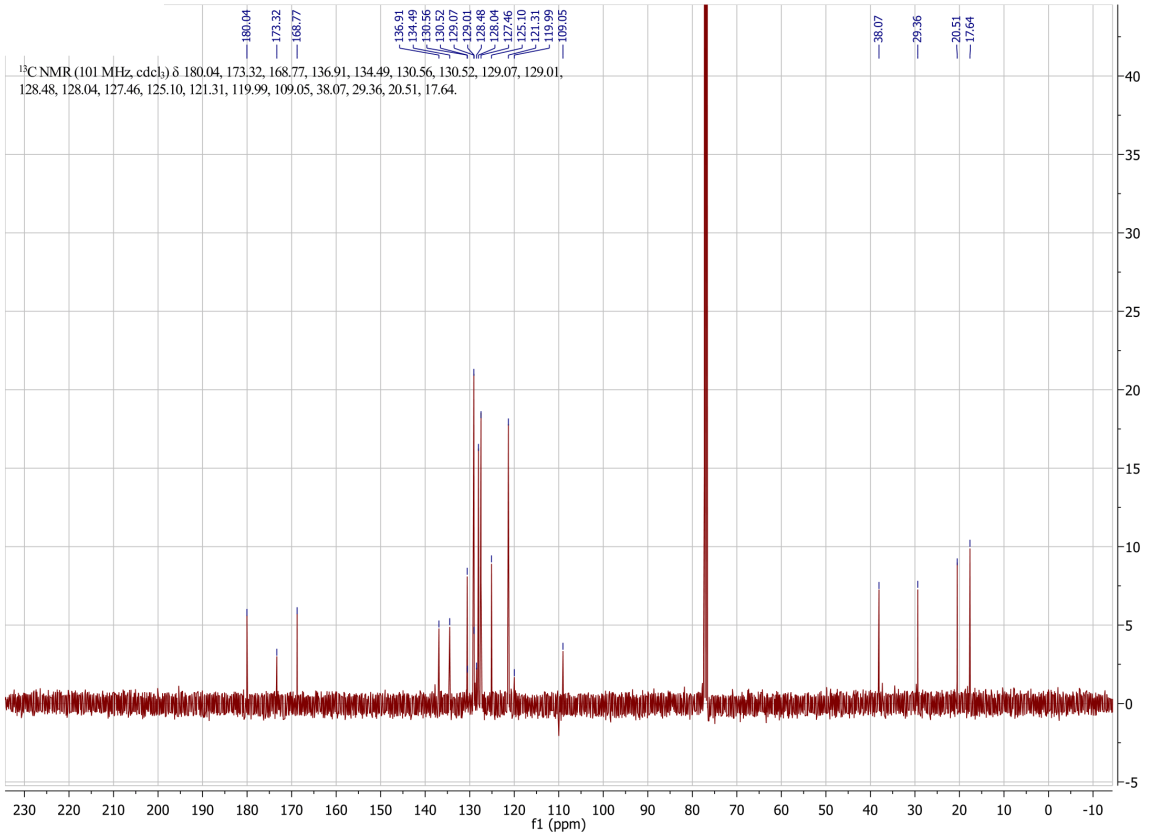

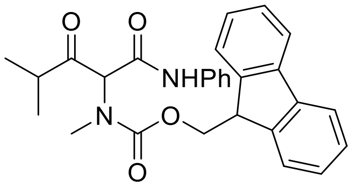

(9H-fluoren-9-yl)methylmethyl(4-methyl-1,3-dioxo-1(phenylamino)pentan-2-yl)carbamate, 14

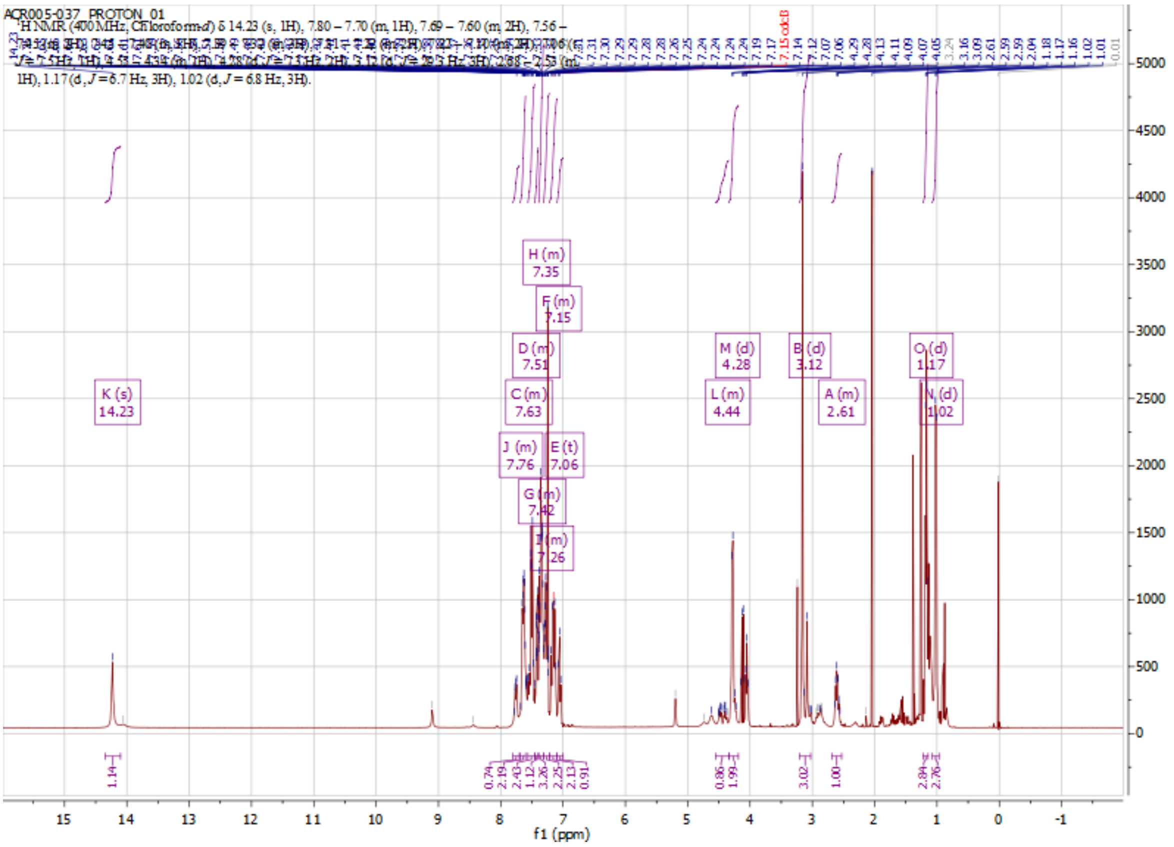

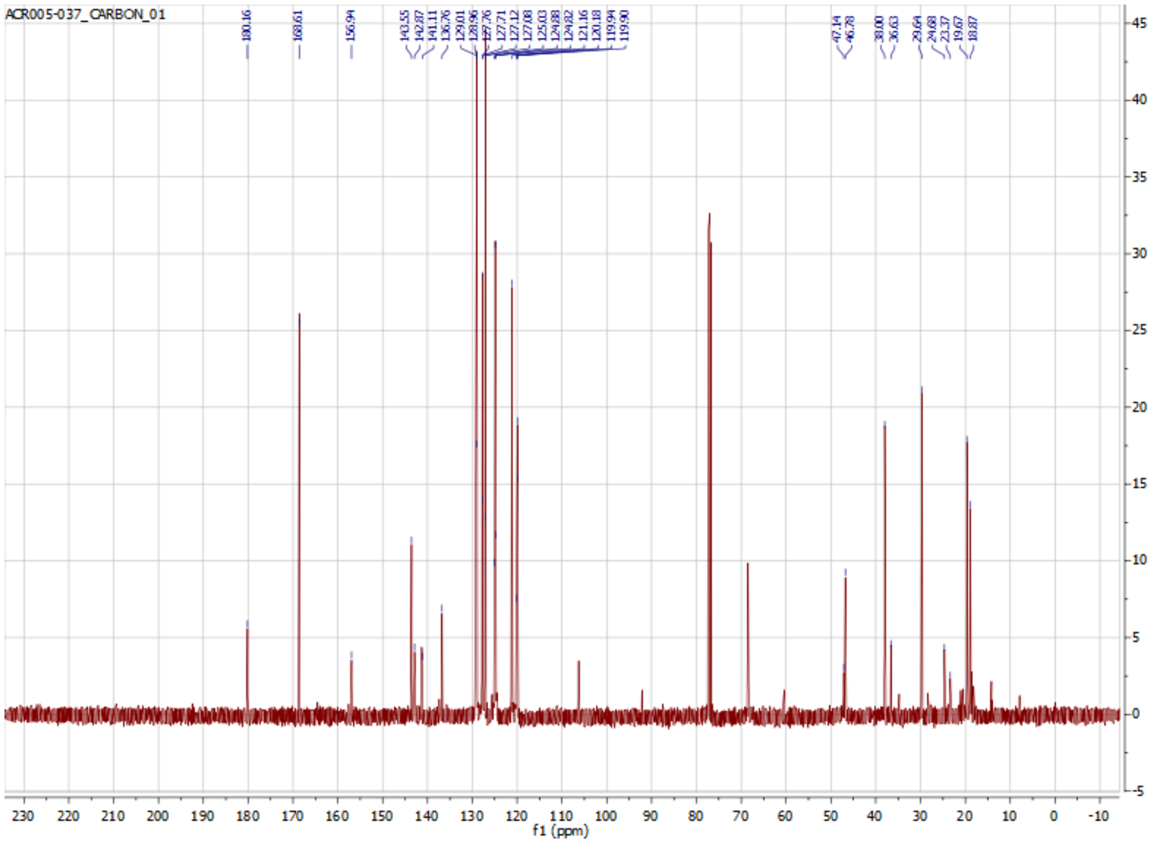

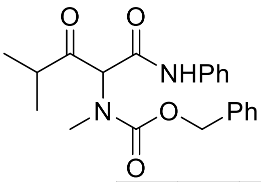

Benzyl methyl(4-methyl-1,3-dioxo-1-(phenylamino)pentan-2-yl)carbamate, 9

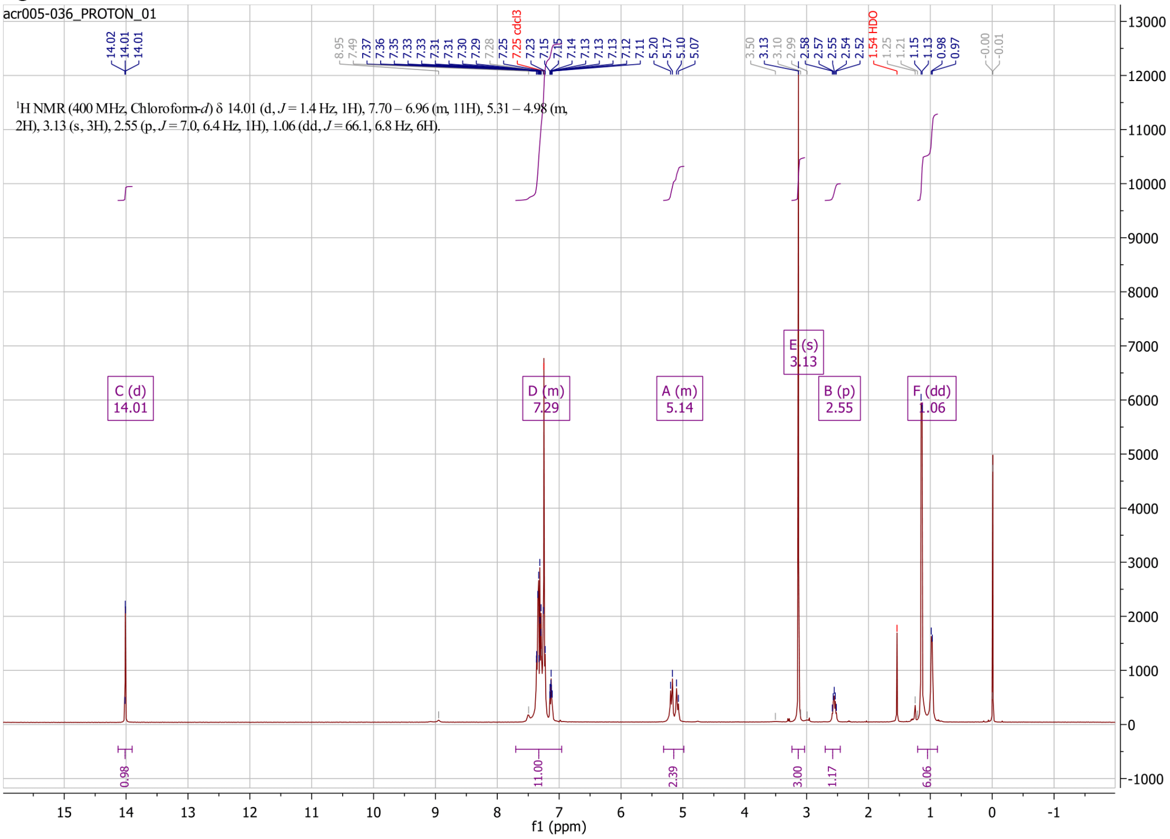

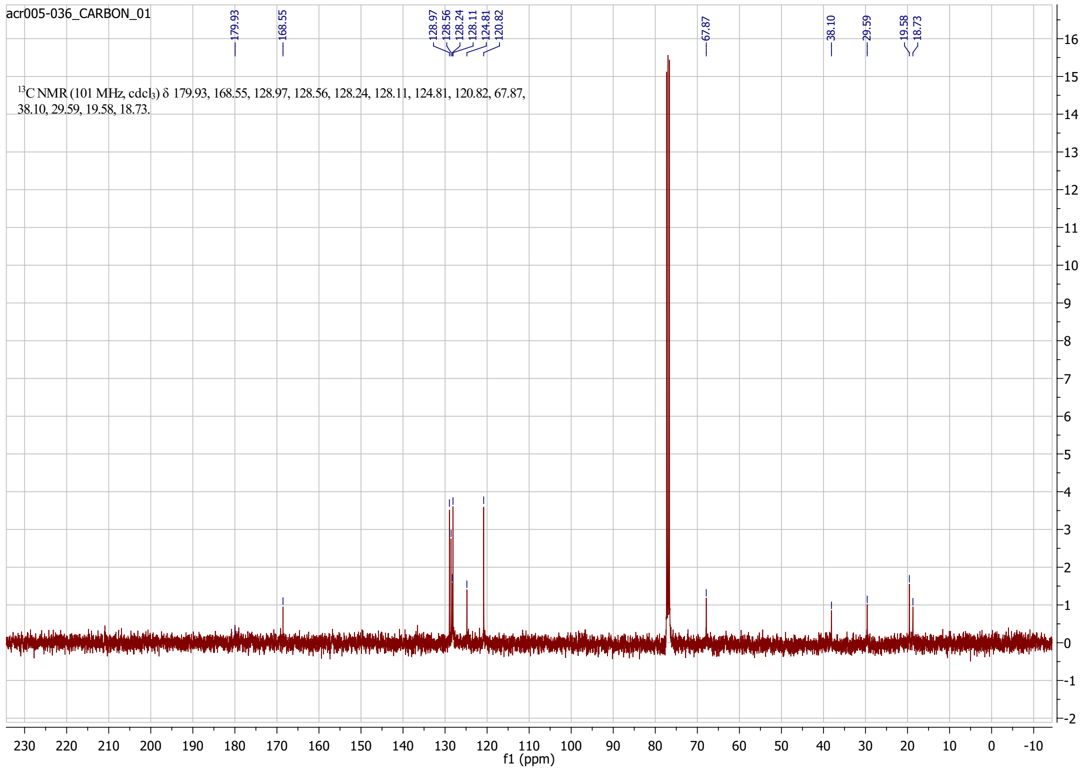

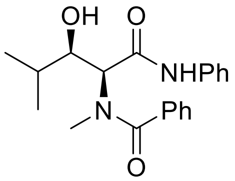

N-((2S,3R)-3-hydroxy-4-methyl-1-oxo-1-(phenylamino)pentan-2-yl)-Nmethyl benzamide, 15

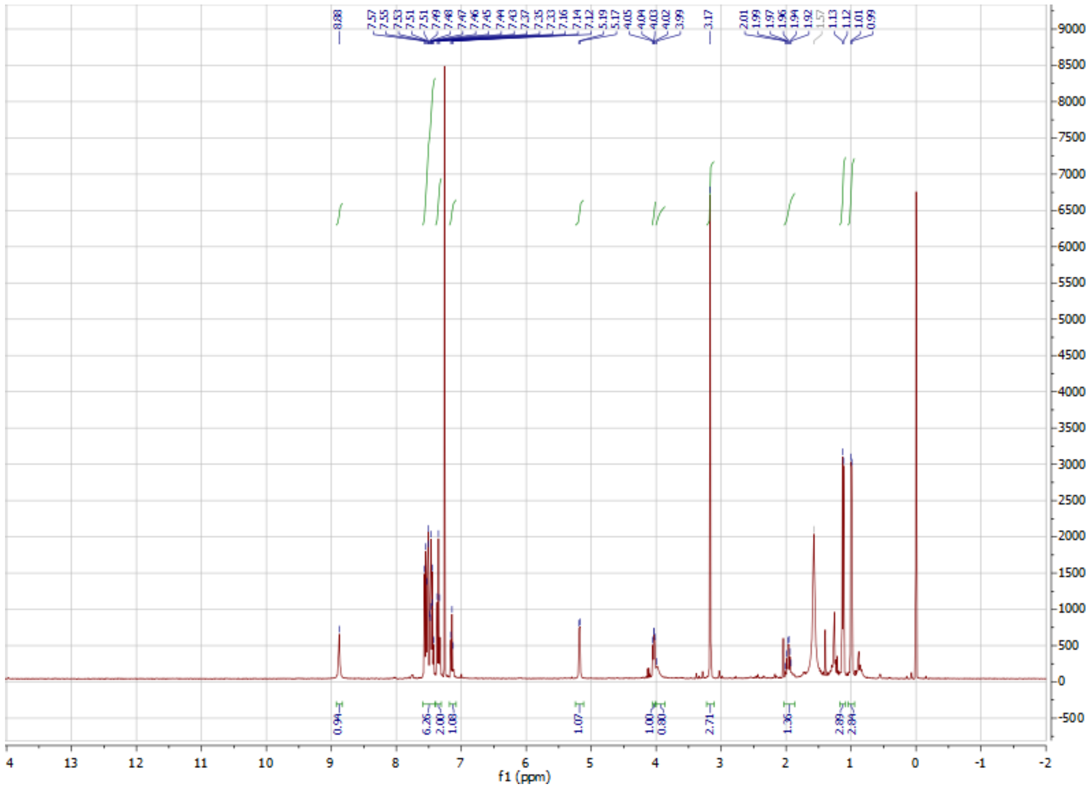

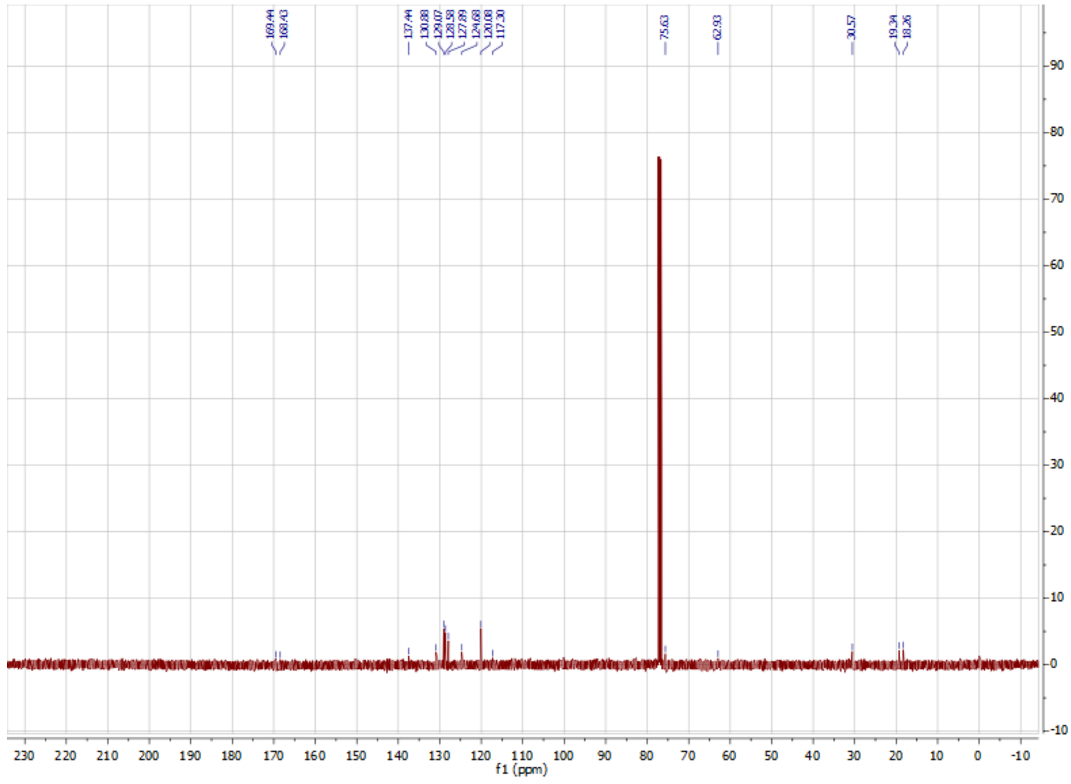

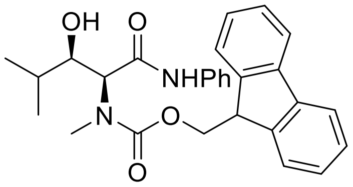

(9H-fluoren-9-yl)methyl((2S,3R)-3-hydroxy-4-methyl-1-oxo-1-(phenyl amino)pentan-2-yl)(methyl) carbamate, 16

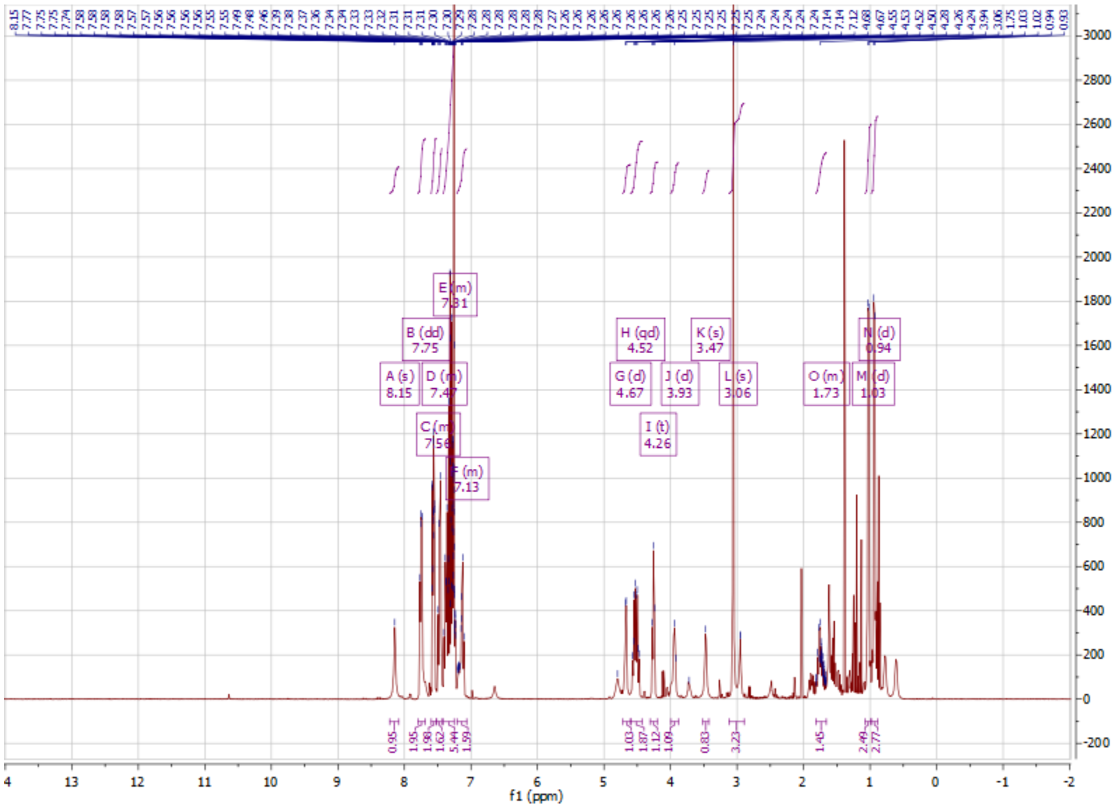

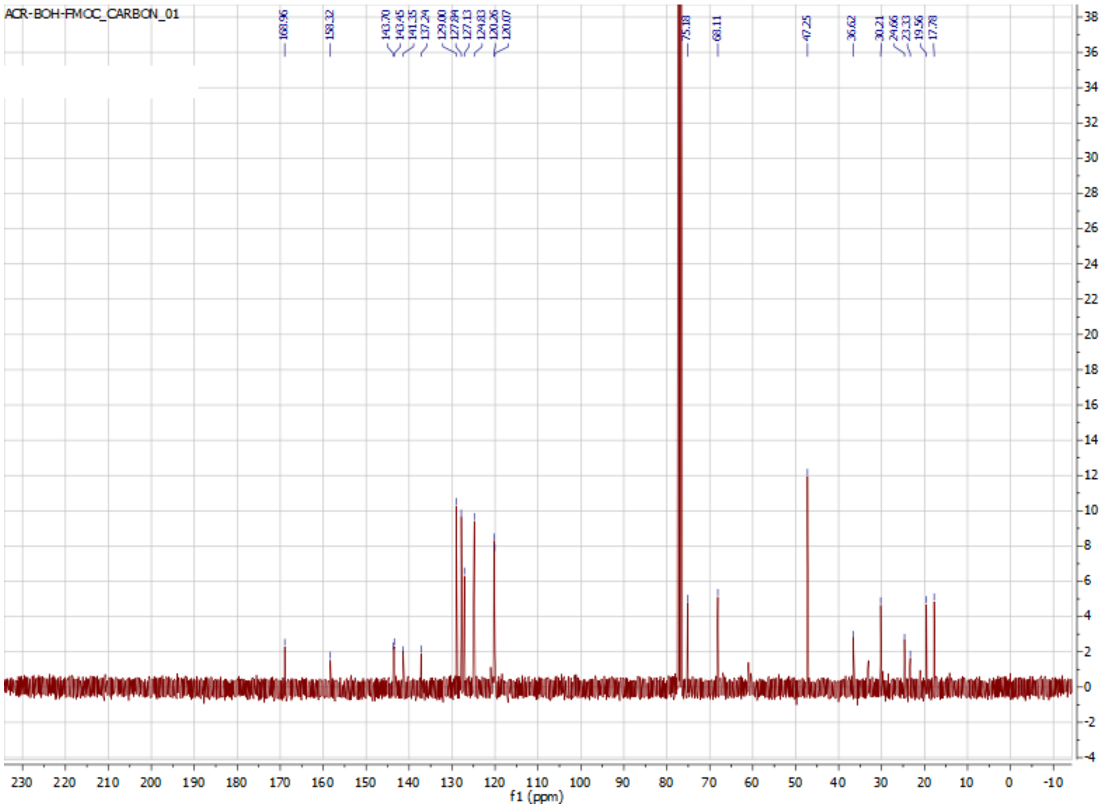

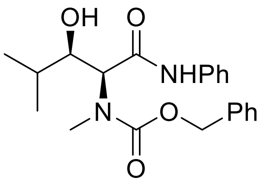

Benzyl ((2S,3R)-3-hydroxy-4-methyl-1-oxo-1-(phenylamino)pentan-2-yl)(methyl) carbamate, 17

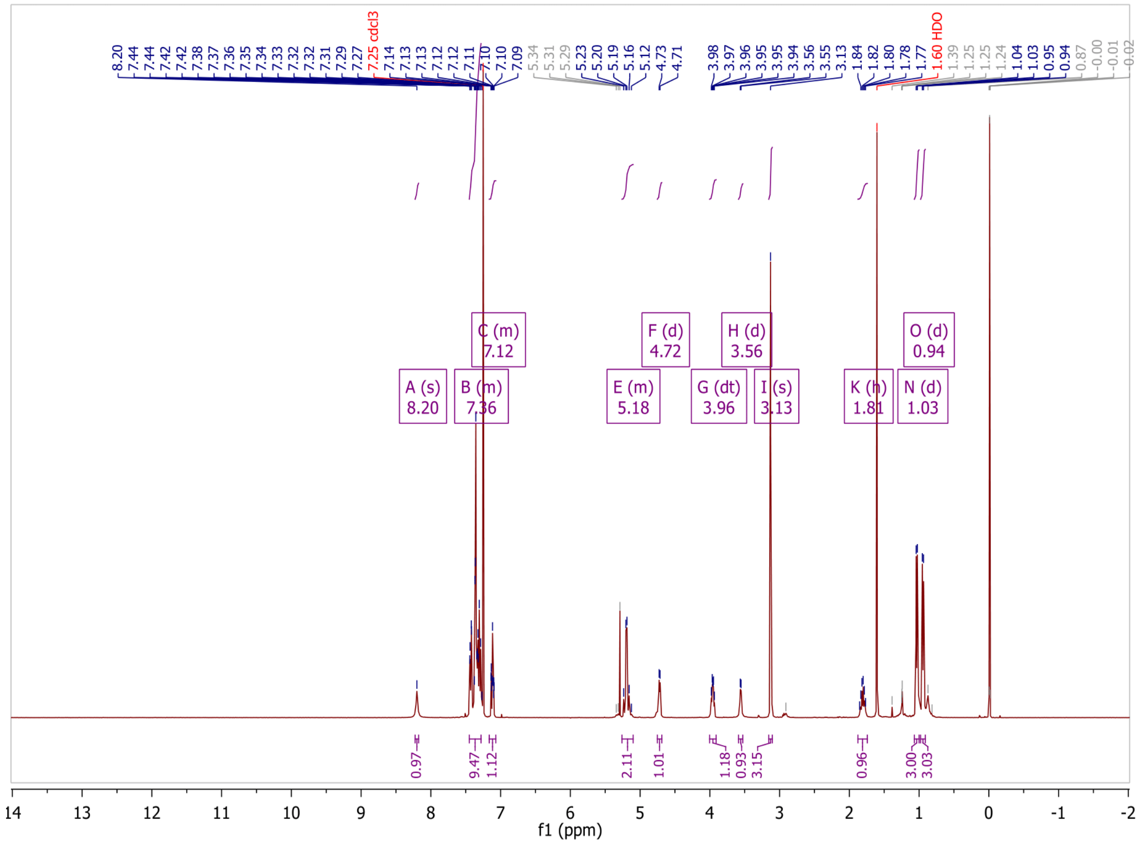

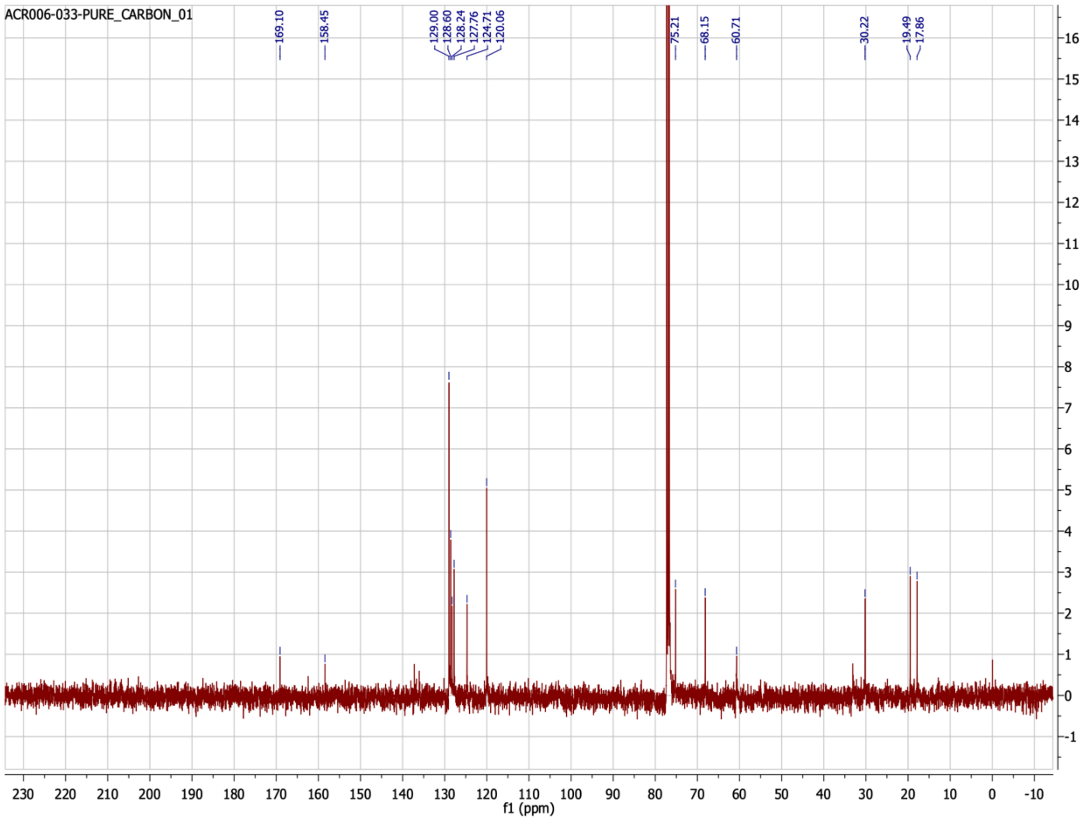

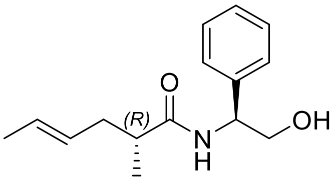

(2R,4E)-N-((S)-2-hydroxy-1-phenylethyl)-2-methylhex-4-enamide – 19

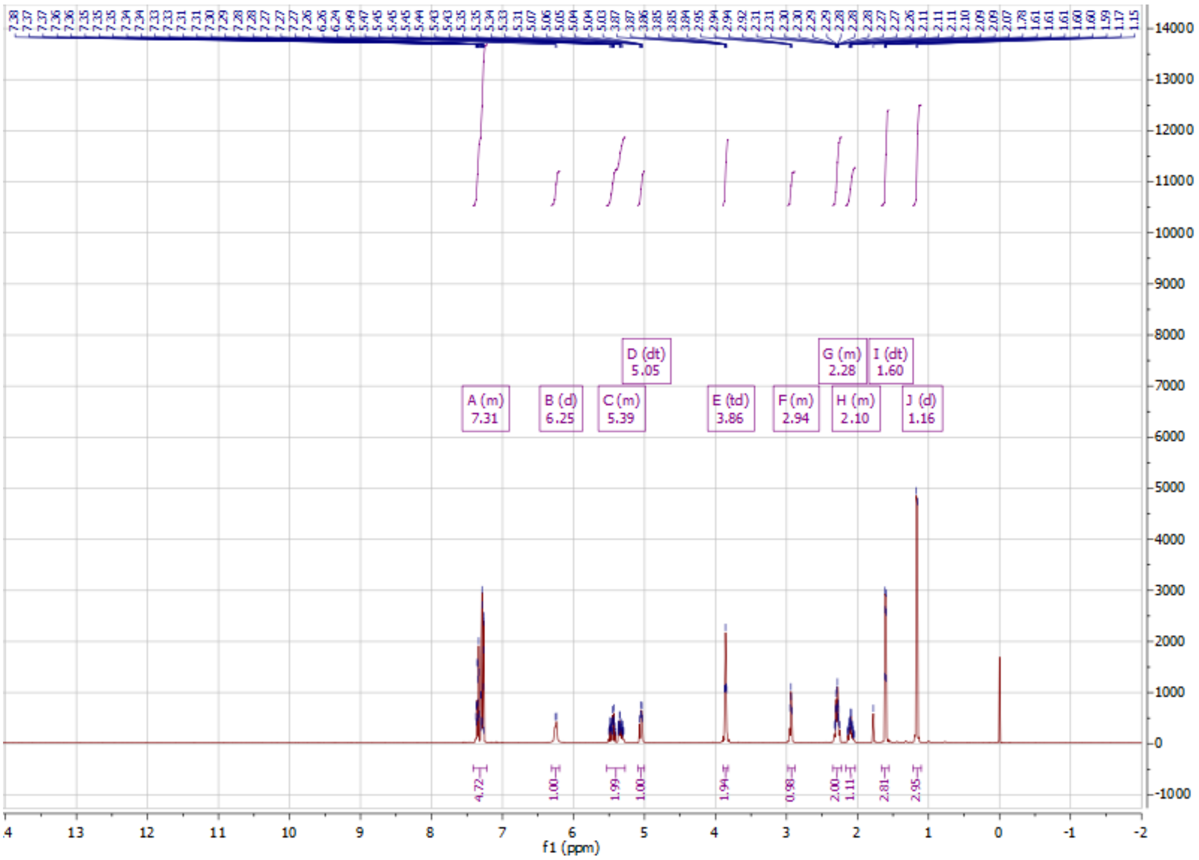

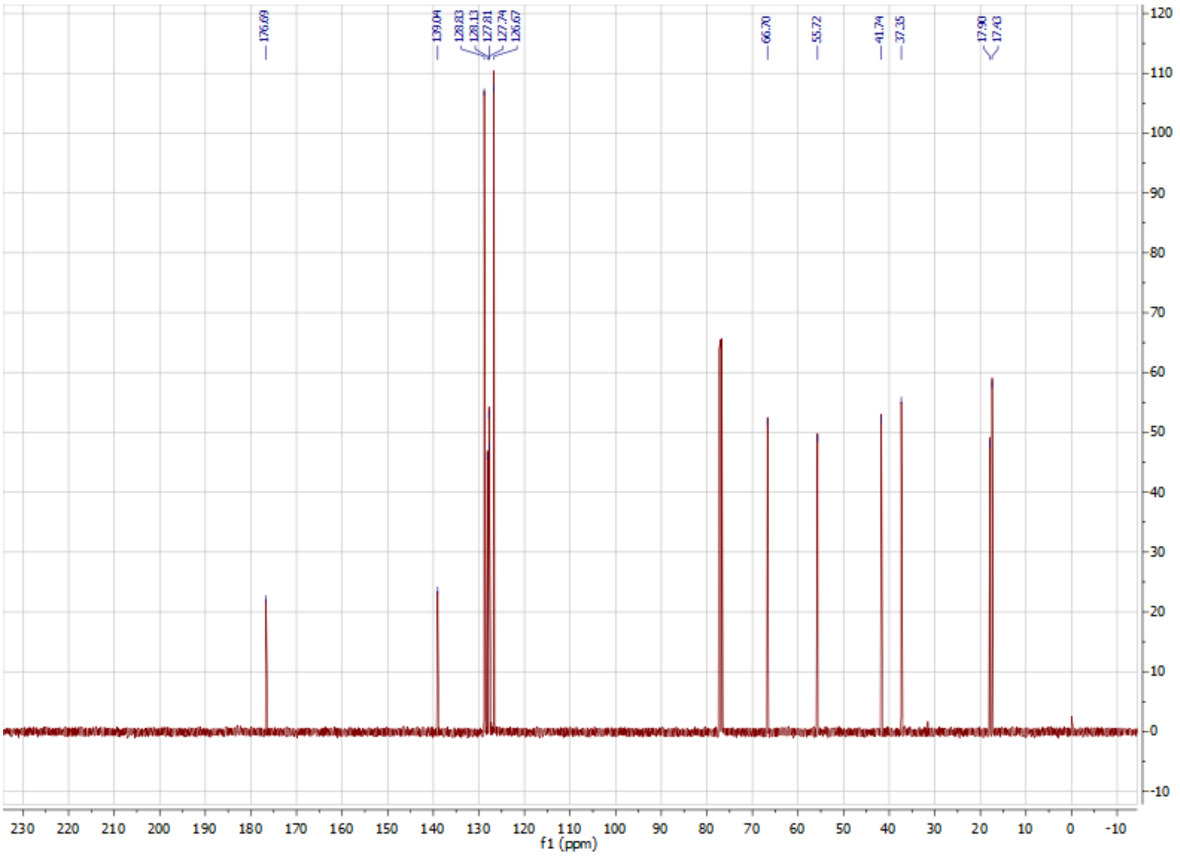

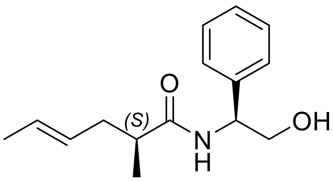

(2S,4E)-N-((S)-2-hydroxy-1-phenylethyl)-2-methylhex-4-enamide – epi-19

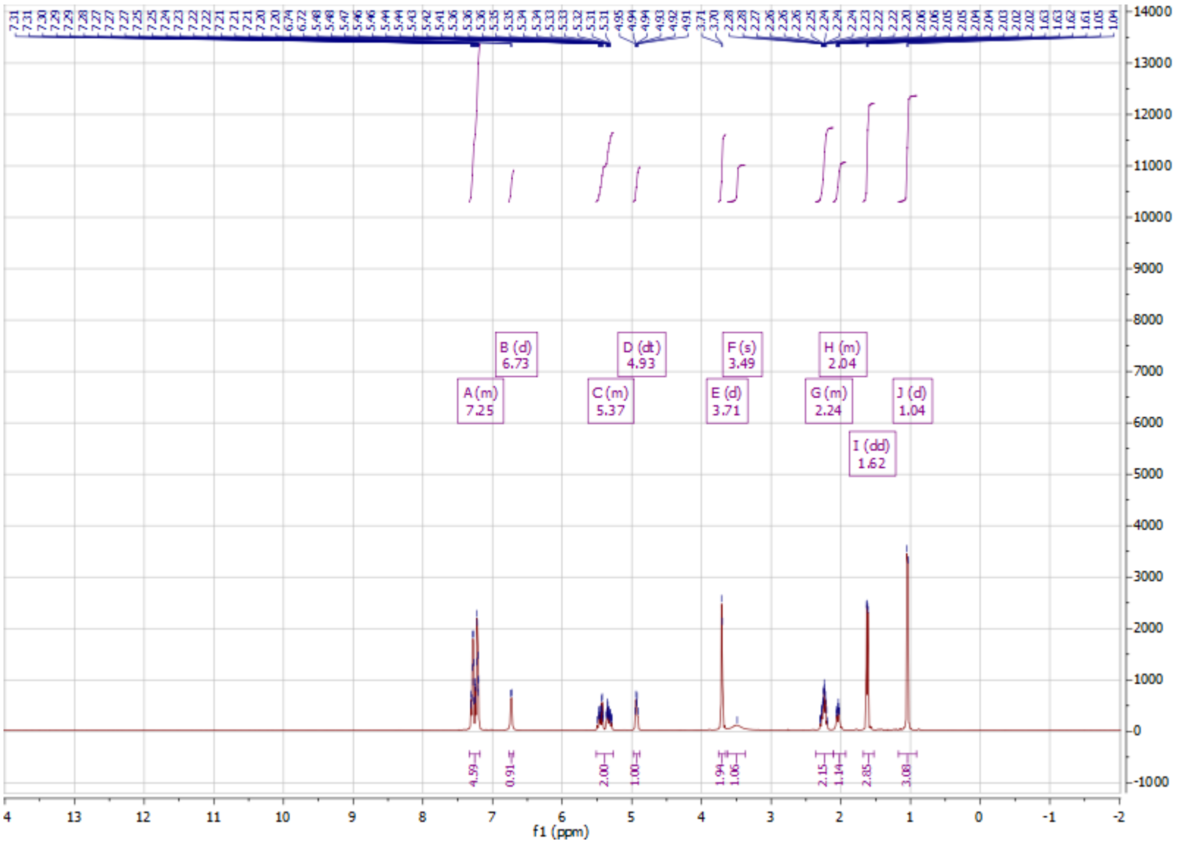

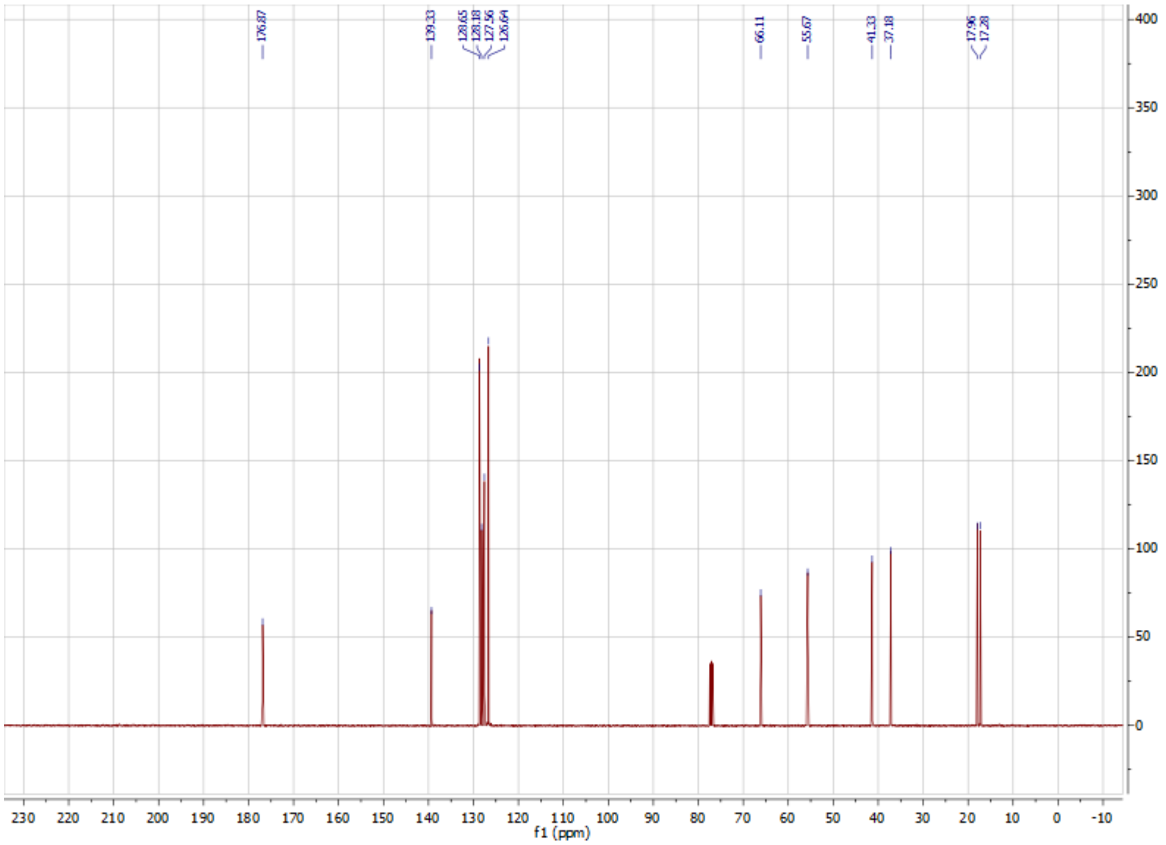

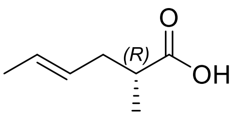

(2R,4E)-2-methylhex-4-enoic acid, 20

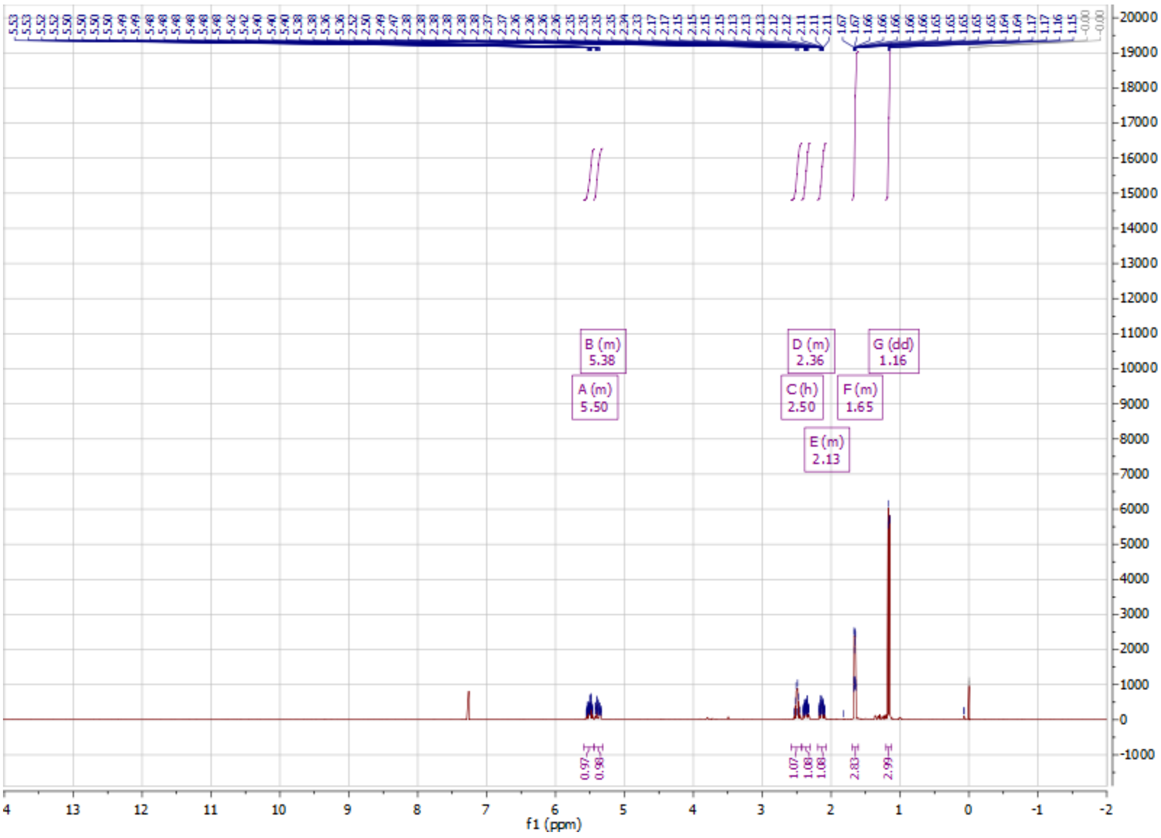

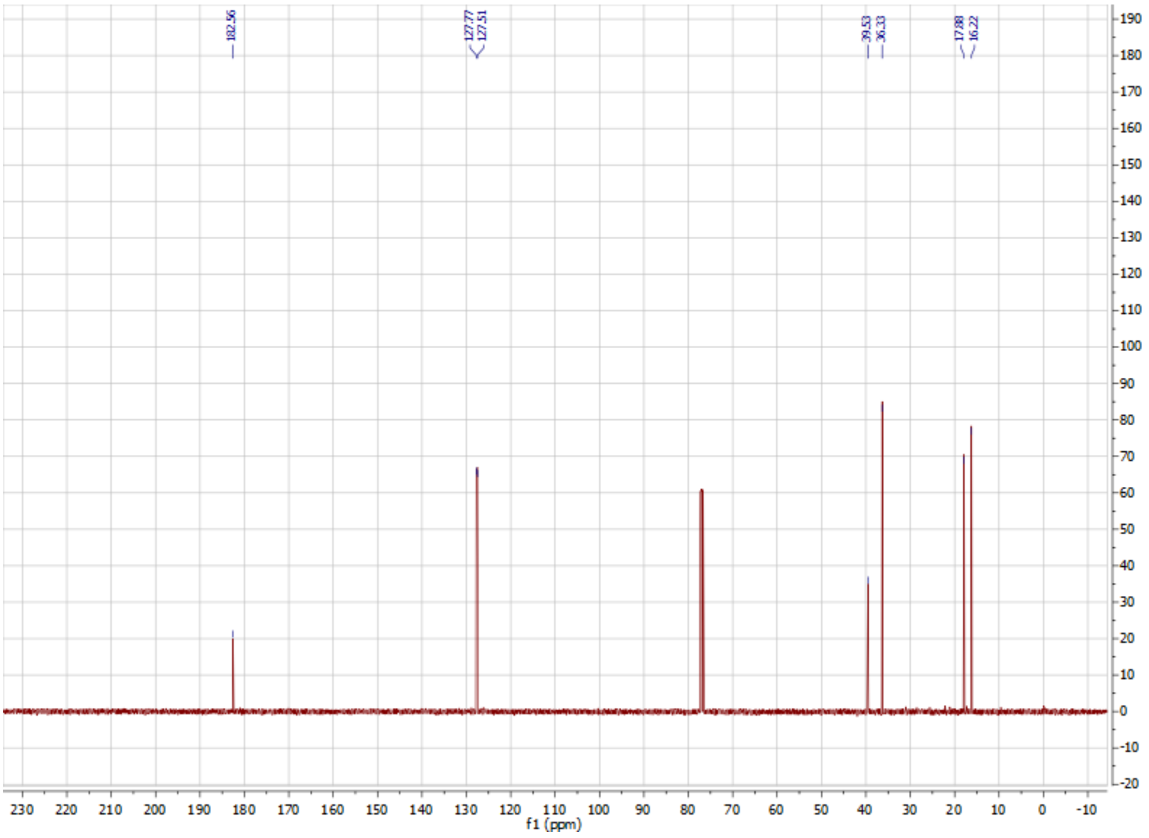

(2R,4E)-1-(1H-benzo[d][1,2,3]triazol-1-yl)-2-methylhex-4-en-1-one, 21

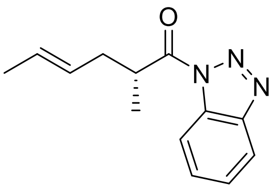

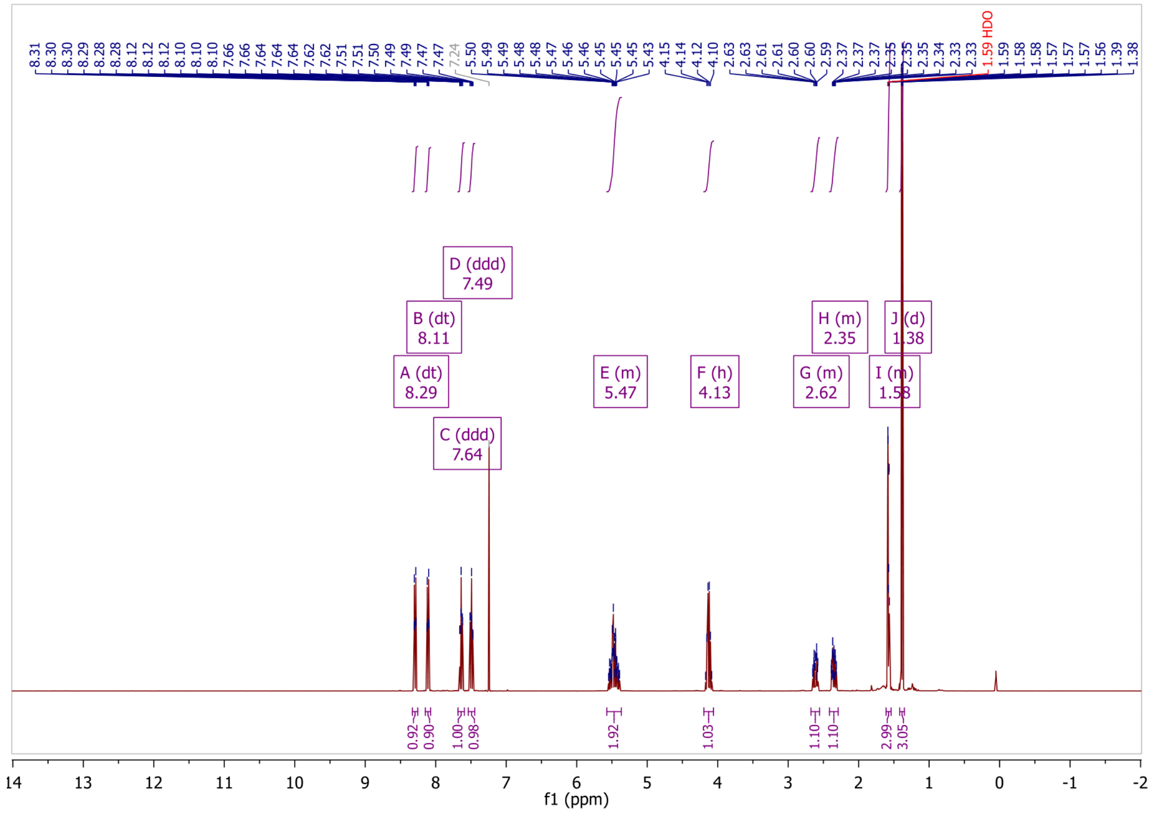

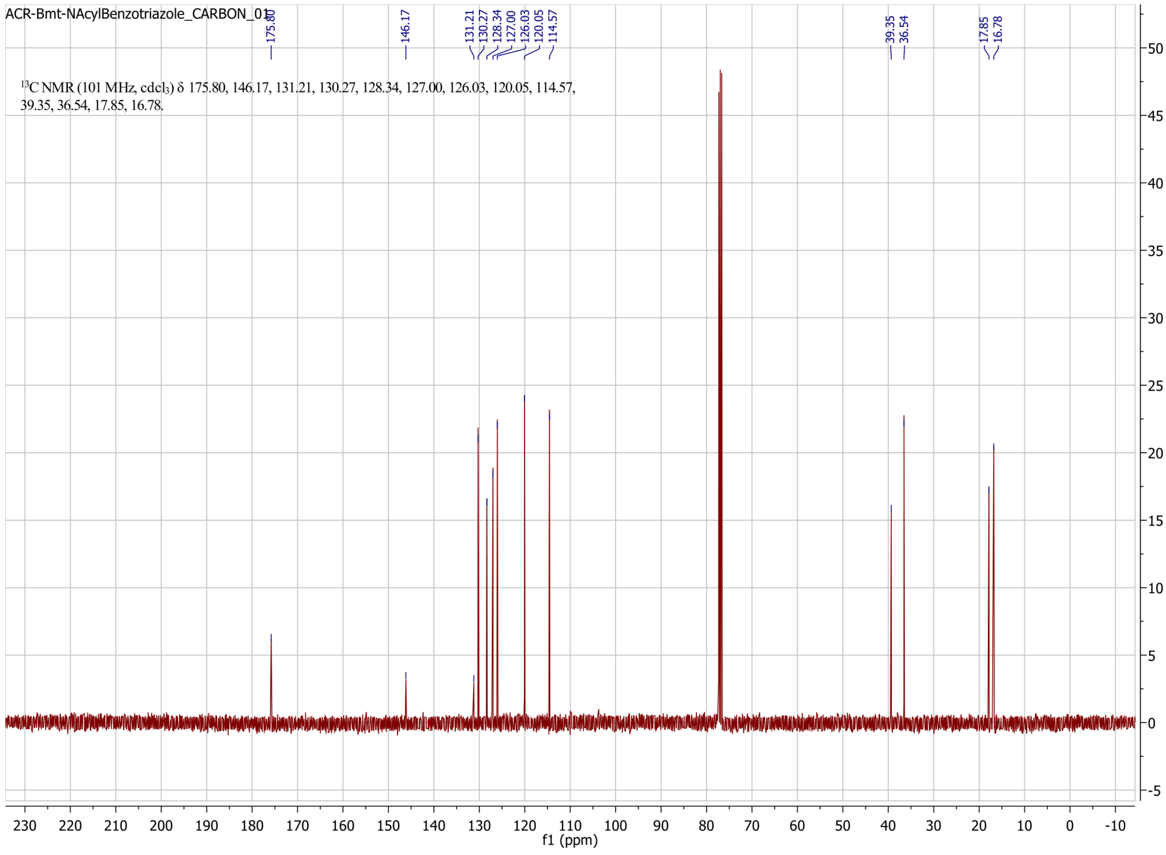

^{13}C NMR (101 MHz, cdcl$_3$) δ 175.80, 146.17, 131.21, 130.27, 128.34, 127.00, 126.03, 120.05, 114.57, 39.35, 36.54, 17.85, 16.78.

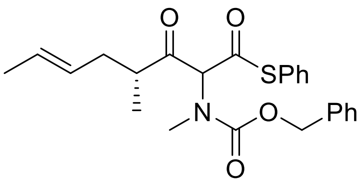

S-phenyl (4R,6E)-2-(((benzyloxy)carbonyl)(methyl)amino)-4-methyl-3-oxooct-6-enethioate, 23

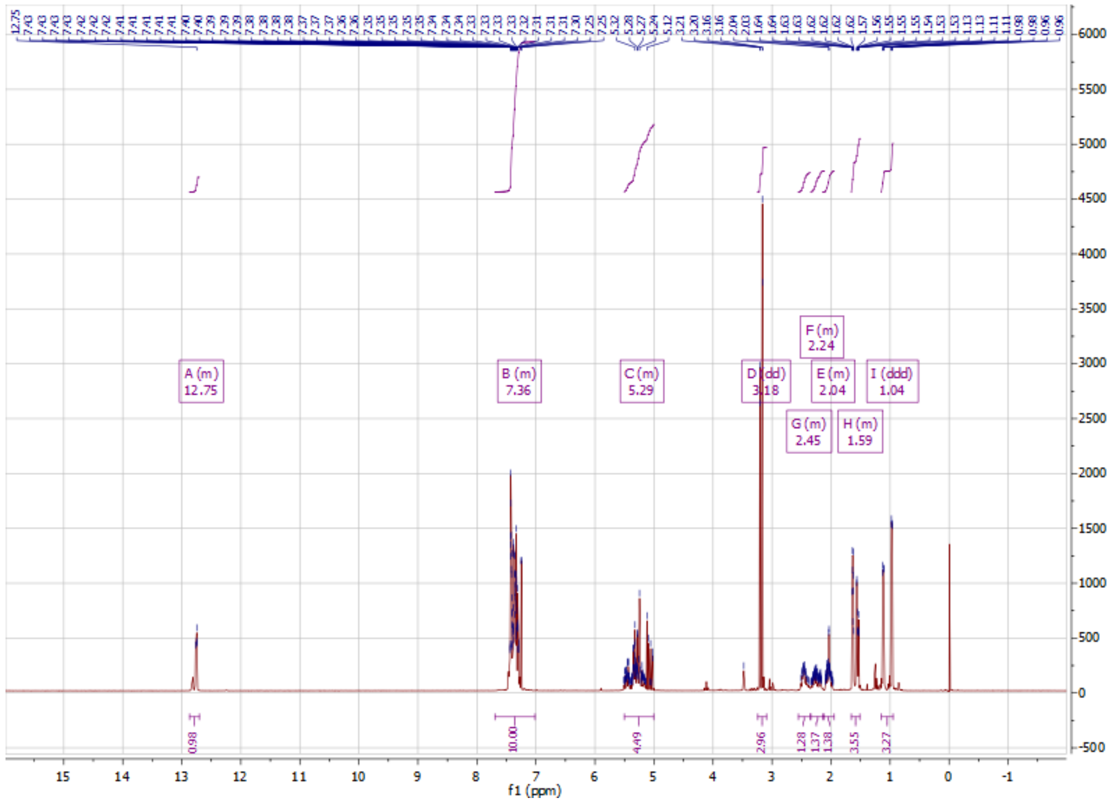

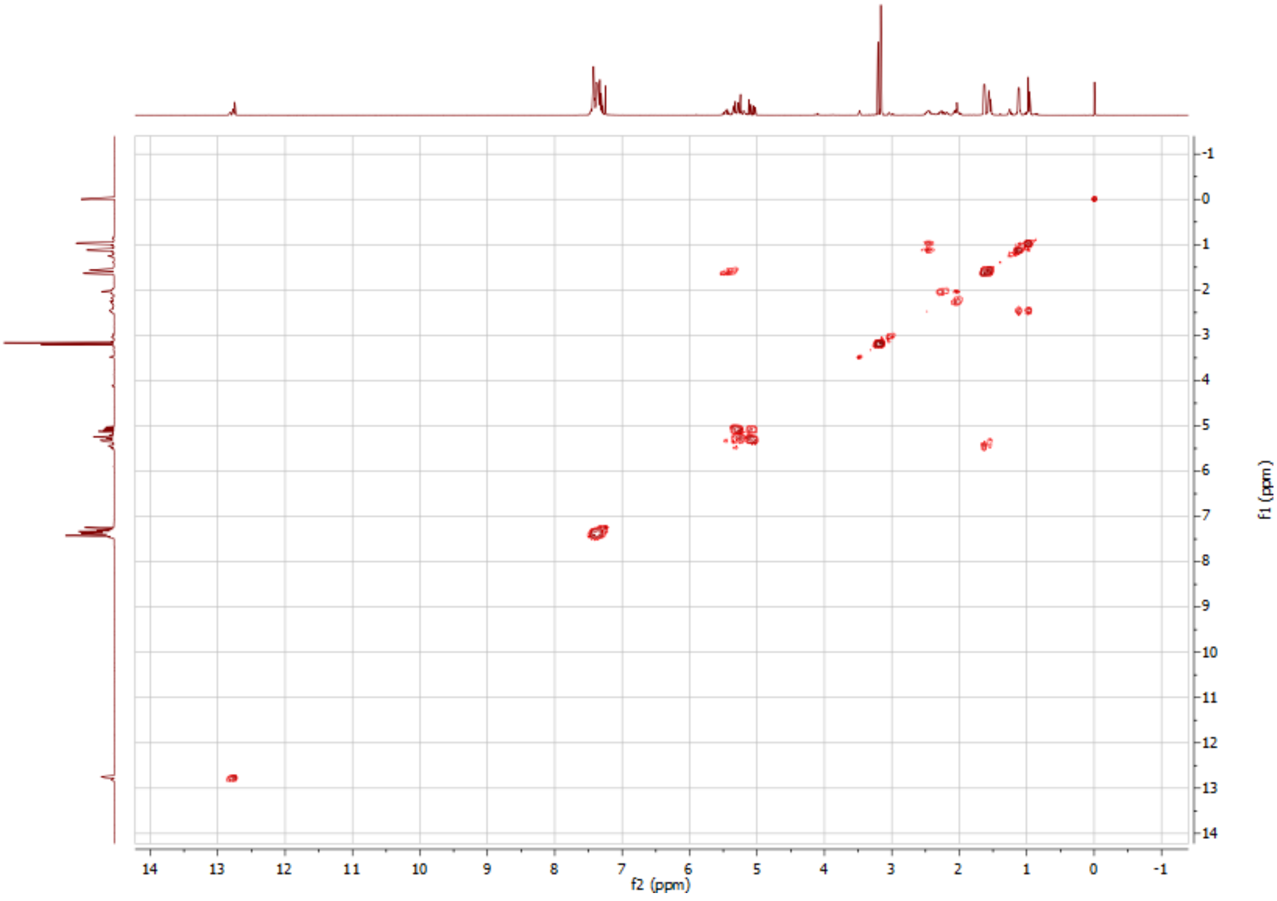

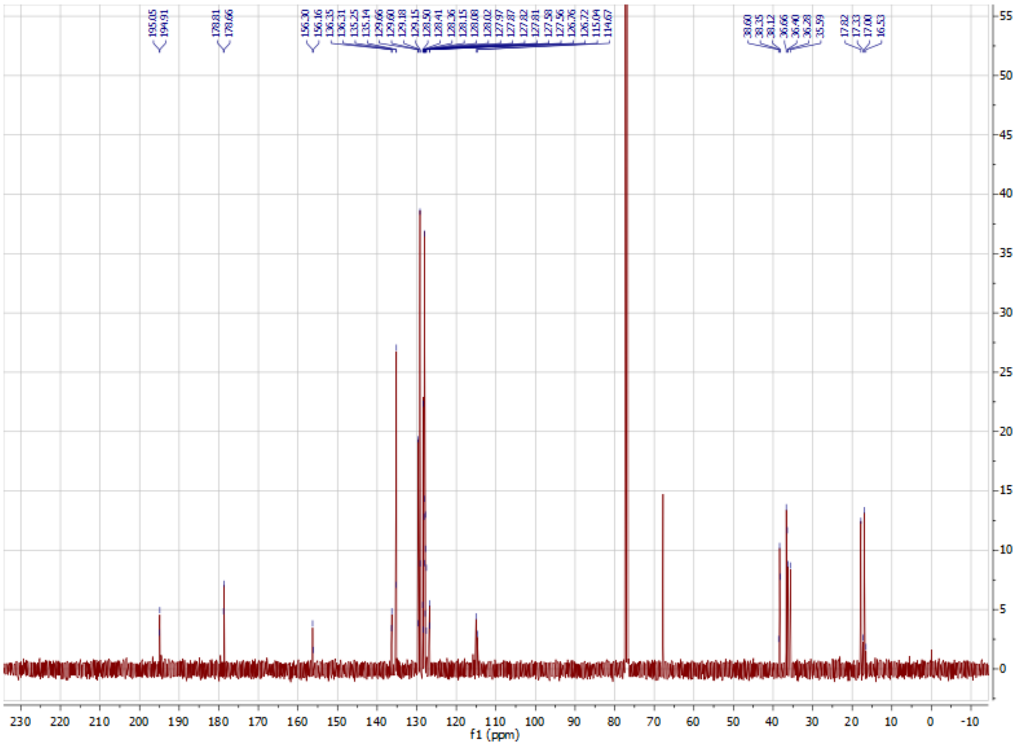

195.05
194.91
178.81
178.66
156.30
156.16
136.35
136.31
135.25
135.14
129.66
129.60
129.18
129.15
128.50
128.41
128.36
128.15
128.08
128.02
127.97
127.87
127.82
127.81
127.58
127.56
126.76
126.72
115.04
114.67
38.60
38.35
38.12
36.66
36.49
36.28
35.59
17.82
17.33
17.00
16.53
f1 (ppm)

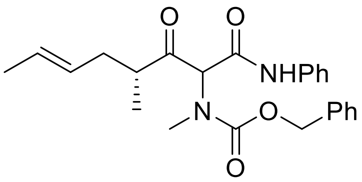

Benzyl methyl((4R,6E)-4-methyl-1,3-dioxo-1 (phenylamino)oct-6-en-2- yl) carbamate, 24

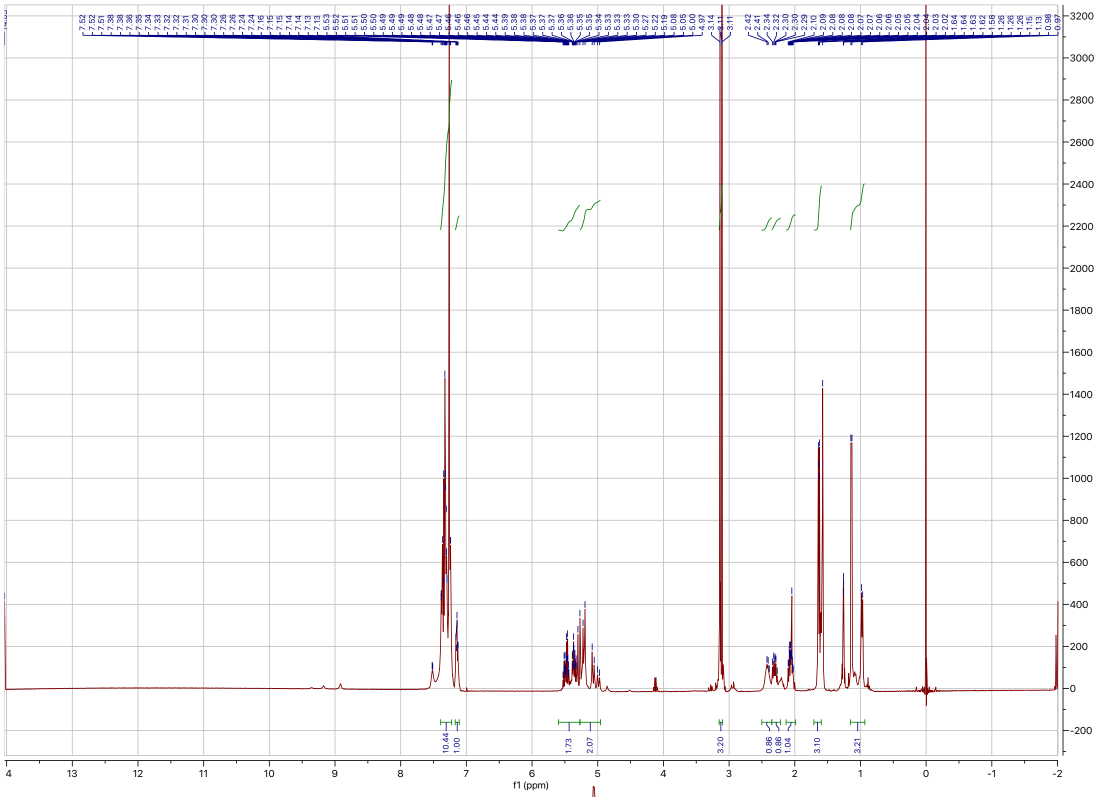

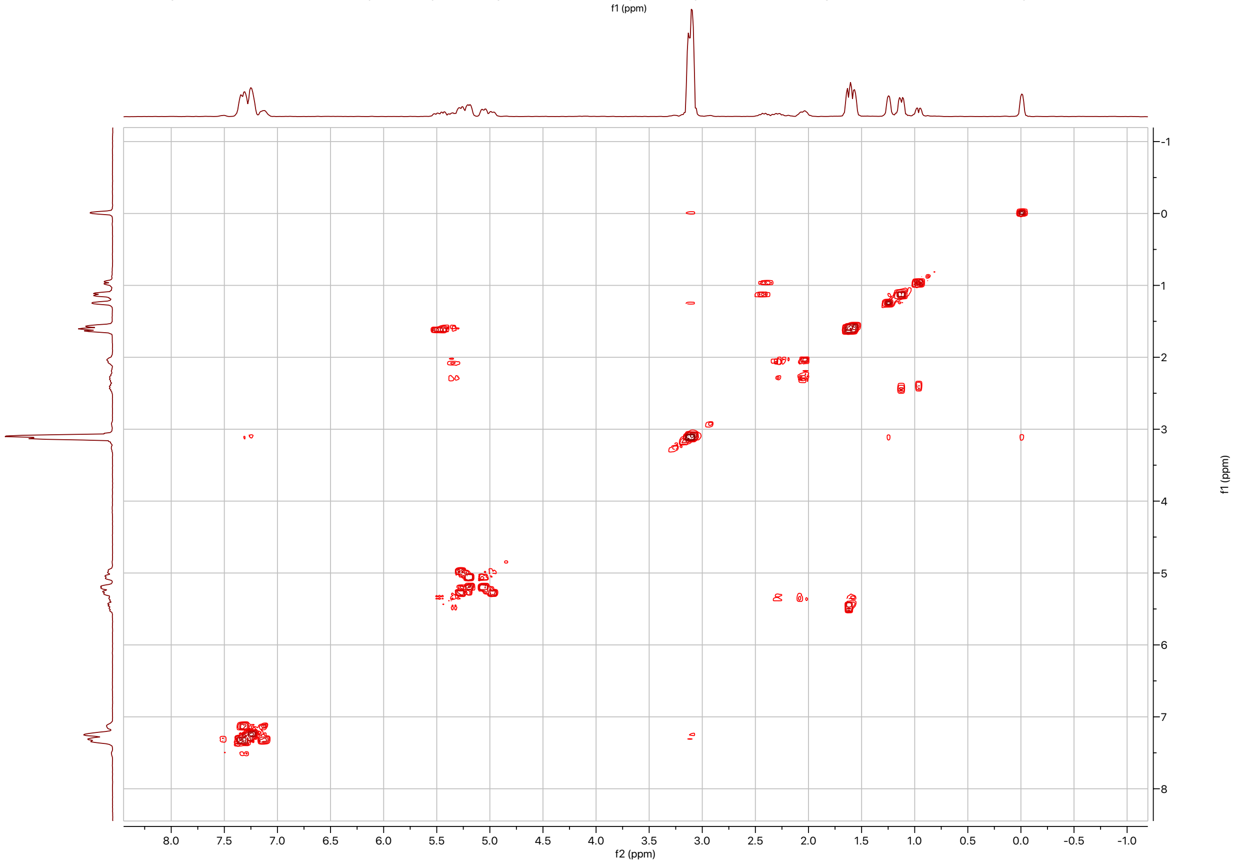

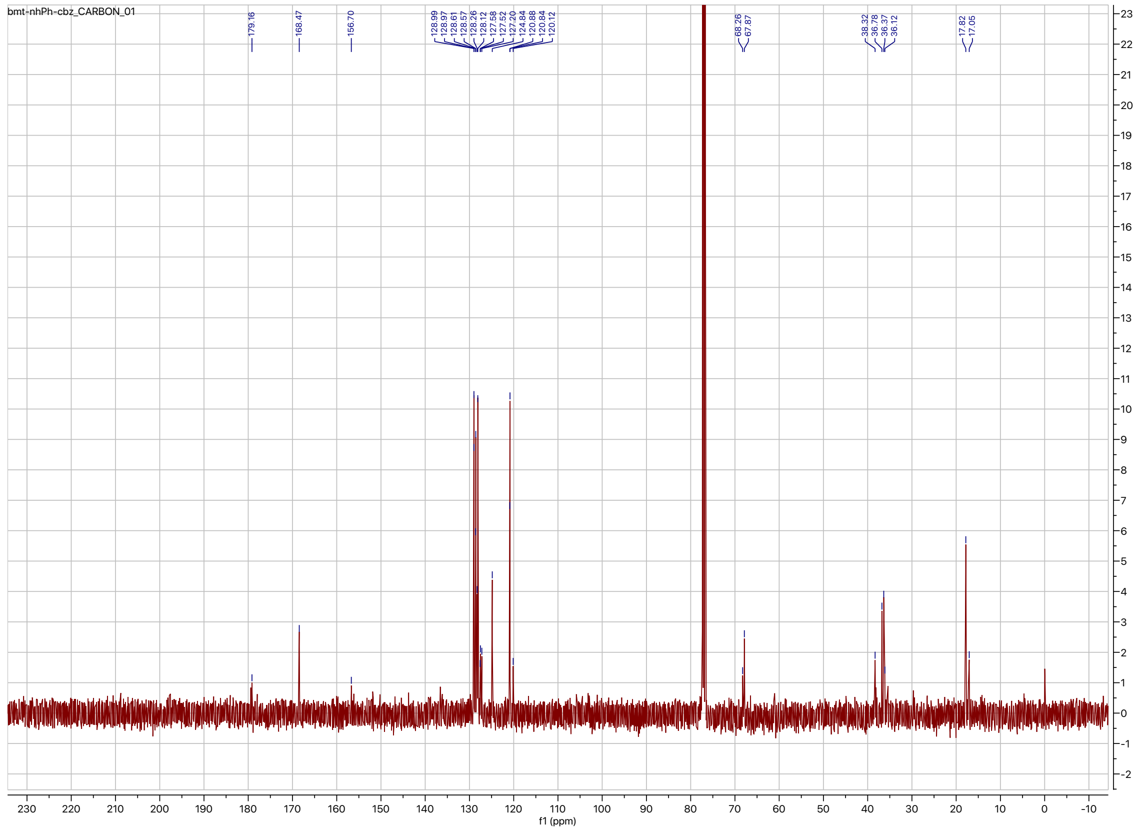

bmt-nhPh-cbz_CARBON_01
179.16
168.47
156.70
128.99
128.97
128.61
128.57
128.26
128.12
127.58
127.52
127.20
124.84
120.88
120.84
120.12
68.26
67.87
38.32
36.78
36.37
36.12
17.82
17.05
f1 (ppm)

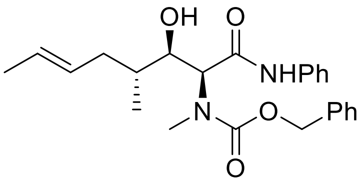

Benzyl ((2S,3R,4R,6E)-3-hydroxy-4-methyl-1-oxo-1 (phenylamino)oct-6-en-2-yl)(methyl)carbamate, 25

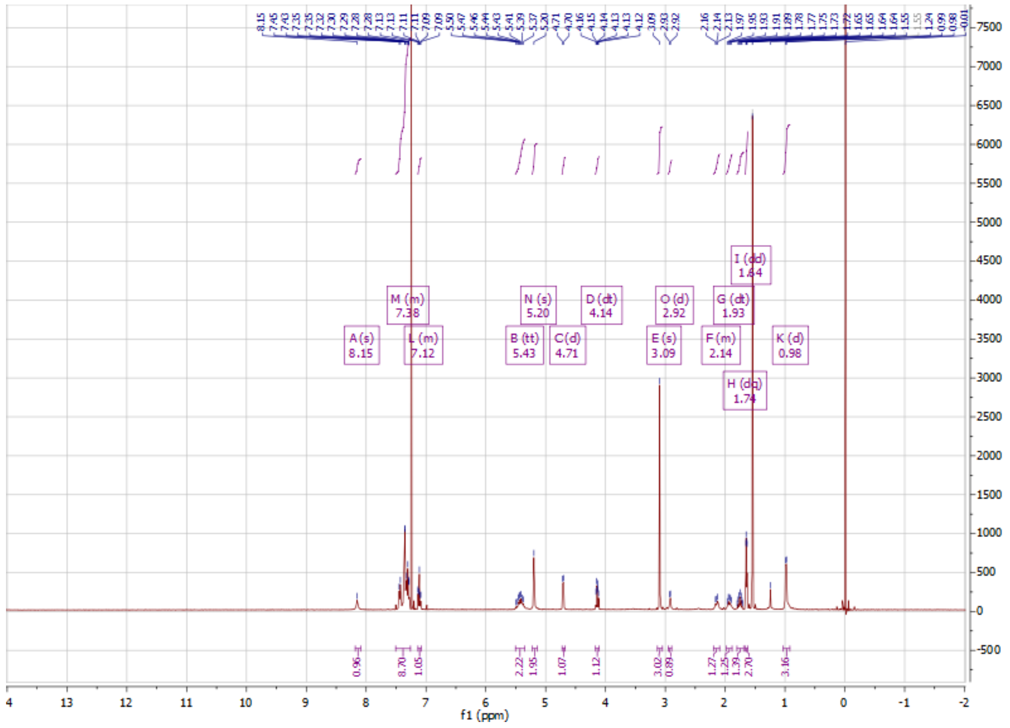

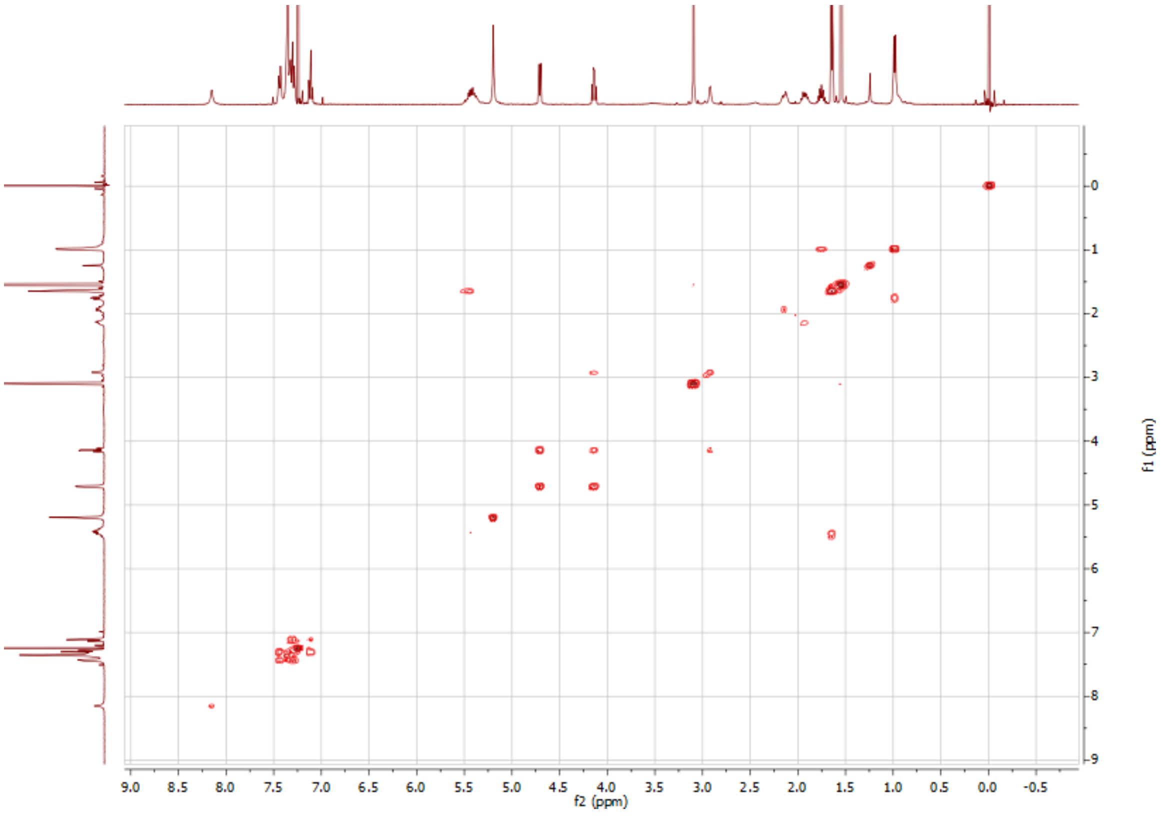

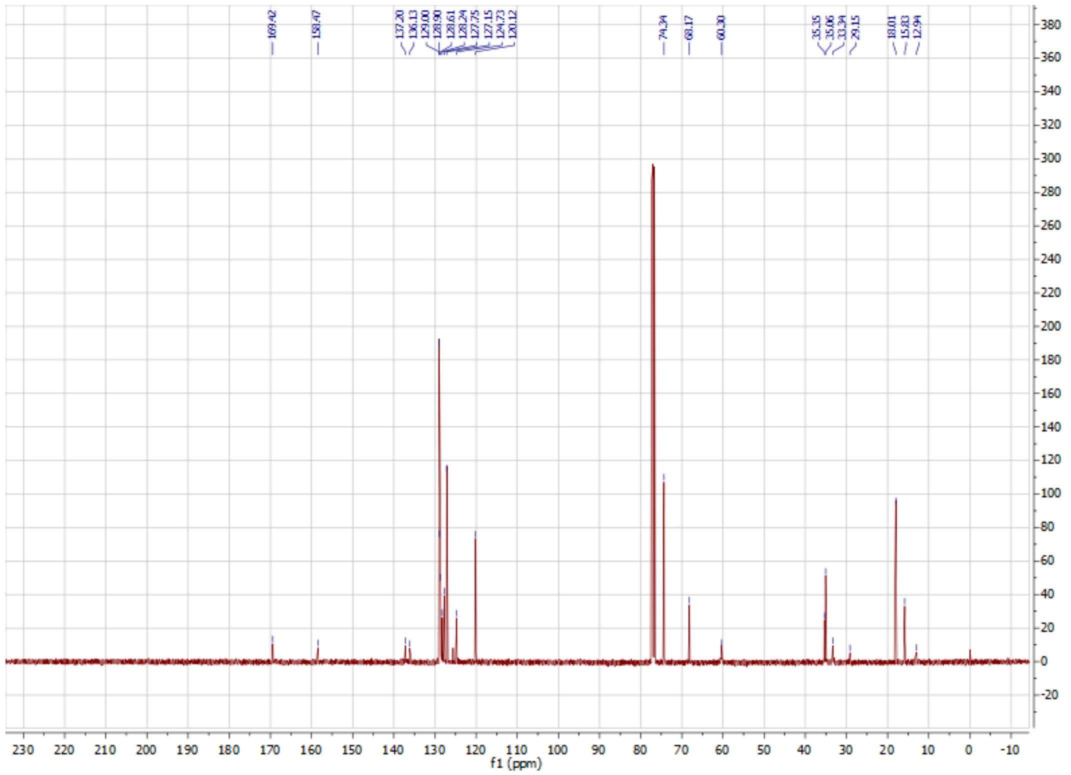

169.42
158.47
137.20
136.13
129.00
128.90
128.61
128.24
127.75
127.15
124.73
120.12
74.34
68.17
60.30
35.35
35.06
33.34
29.15
18.01
15.83
12.94
f1 (ppm)

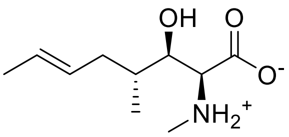

(2S,3R,4R,E)-3-hydroxy-4-methyl-2-(methylamino)oct-6-enoic acid, MeBmt, 2

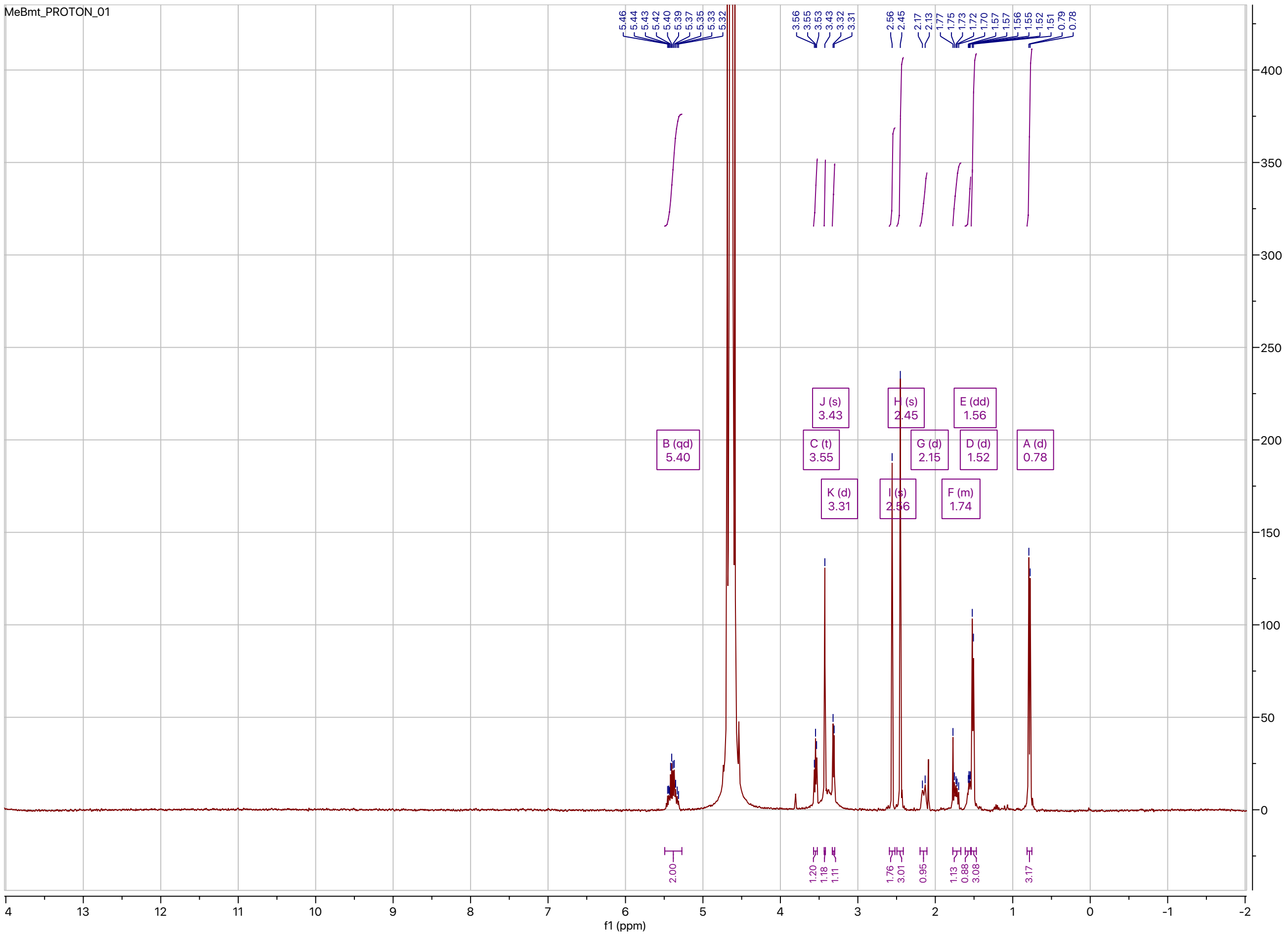

Qualitative Compound Report

**Data File**	ACR008-002.d	**Sample Name**	ACR008-002
**Sample Type**	Sample	**Position**	Vial 18
**Instrument Name**	Instrument 1	**User Name**	
**Acq Method**	JUL13.m	**Acquired Time**	7/19/2017 4:01:59 PM
**IRM Calibration Status**	Success	**DA Method**	Formula confirmation_update03.m
**Comment**	201.14		

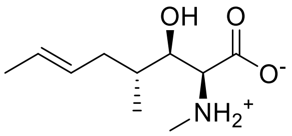

**Sample Group**		**Info.**	
**Formula1**	C10H19NO3	**Fragmentor**	
**VCap**		**Formula2**	
**Skimmer**			

Fragmentor Voltage 175 **Collision Energy** 0 **Ionization Mode** ESI

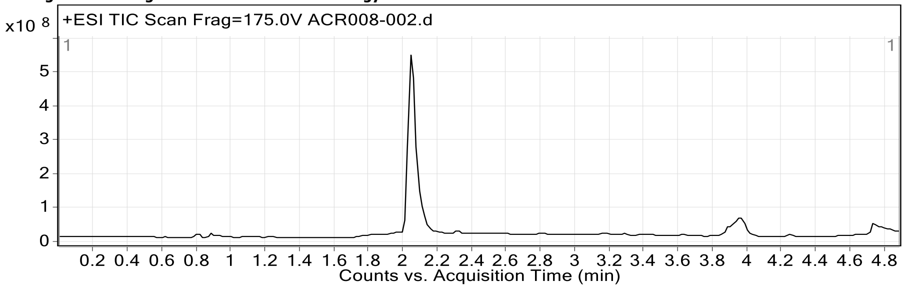

Fragmentor Voltage **Collision Energy** **Ionization Mode**

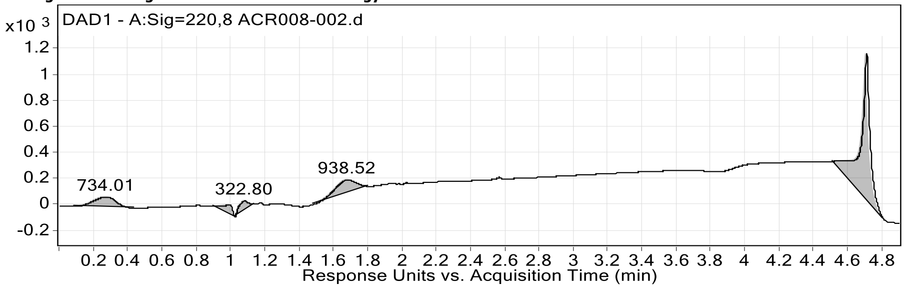

User Chromatogram Peak List

RT	Height	Area	Width
0.272	78.37	734.01	0.355
0.988	70.6	278.63	0.125
1.078	75.58	322.8	0.117
1.678	102.65	938.52	0.327
4.703	1119.2	4205.03	0.295

Compound Table

Compound Label	RT	Mass	Abund	Formula	Tgt Mass	Diff (ppm)	MFG Formula	DB Formula
Cpd 1:	2.04	201.1368	5593432	C10H19NO3	NaN	NaN	C10H19NO3	C10H19NO3

Compound Label	*m/z*	RT	Algorithm	Mass
Cpd 1:	227.1298	2.04	Find By Formula	201.1368

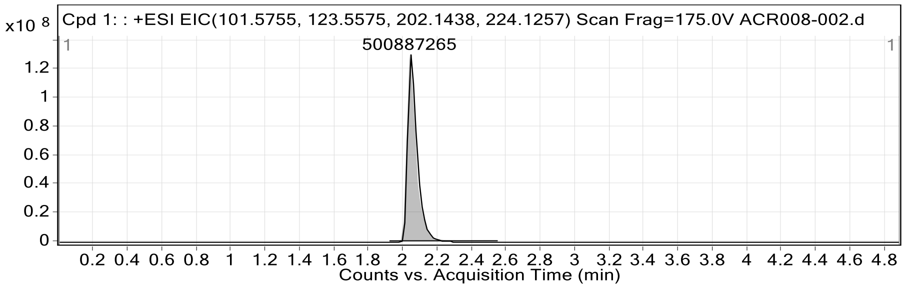

MS Spectrum

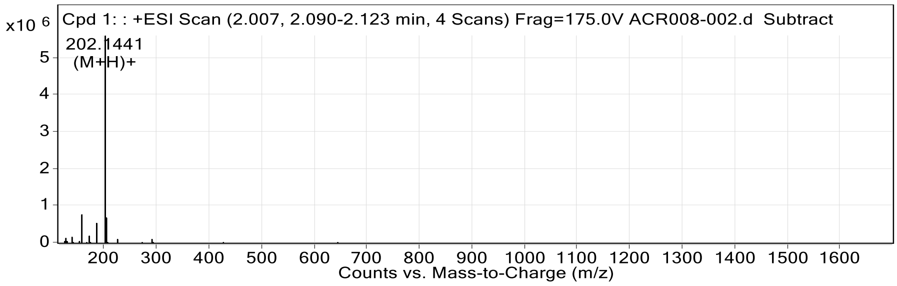

MS Zoomed Spectrum

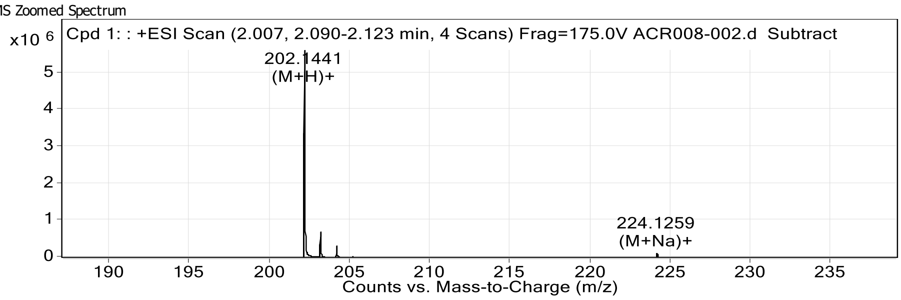

MS Spectrum Peak List

m/z	*Calc m/z*	Diff(ppm)	z	Abund	Formula	Ion
202.1441	202.1438	1.68	1	5599180.3	C10H20NO3	(M+H)+
202.1441				5599180.3		
203.1477	203.147	3.4	1	684777.5	C10H20NO3	(M+H)+
204.1573	204.1491	39.99	1	328224	C10H20NO3	(M+H)+
205.1606	205.1517	43.47	1	34744.8	C10H20NO3	(M+H)+
224.1259	224.1257	0.98	1	129262.5	C10H19NNaO3	(M+Na)+
225.1279	225.1289	-4.52	1	14634	C10H19NNaO3	(M+Na)+
226.1377	226.131	29.34	1	5752.2	C10H19NNaO3	(M+Na)+
227.1298	227.1336	-16.66	1	720	C10H19NNaO3	(M+Na)+

--- End Of Report ---

Benzyl (1-cyclopropyl-1,3-dioxo-3-(phenylamino)propan-2-yl)(methyl)carbamate

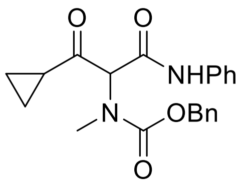

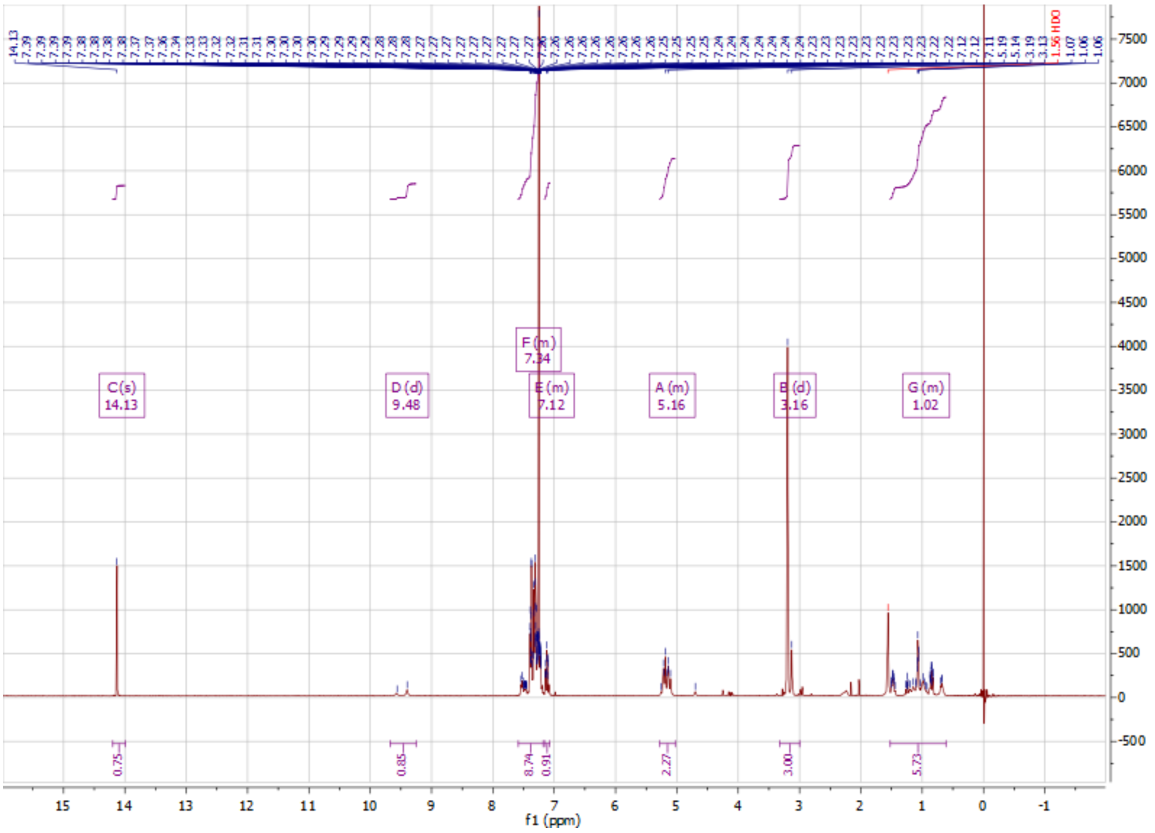

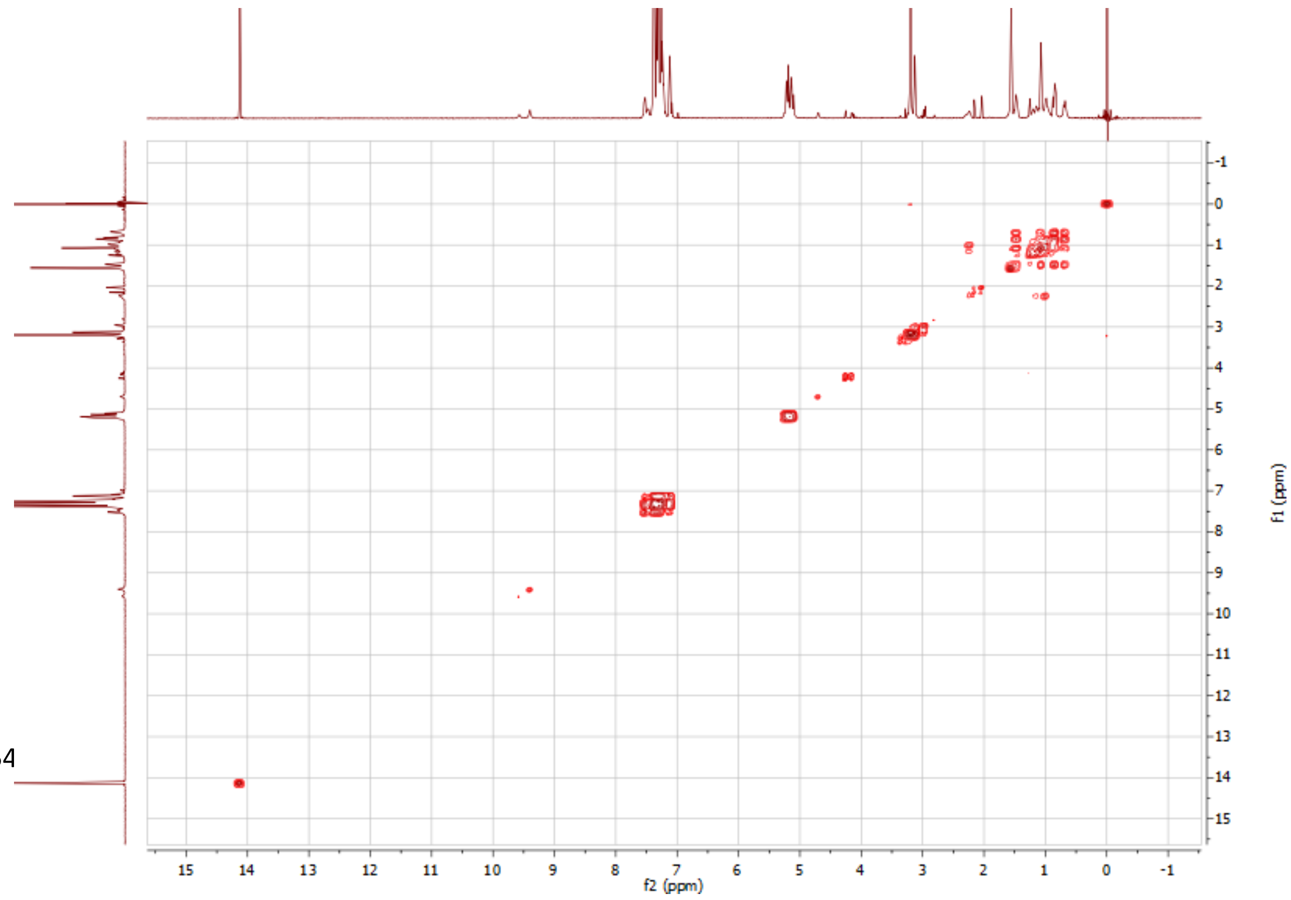

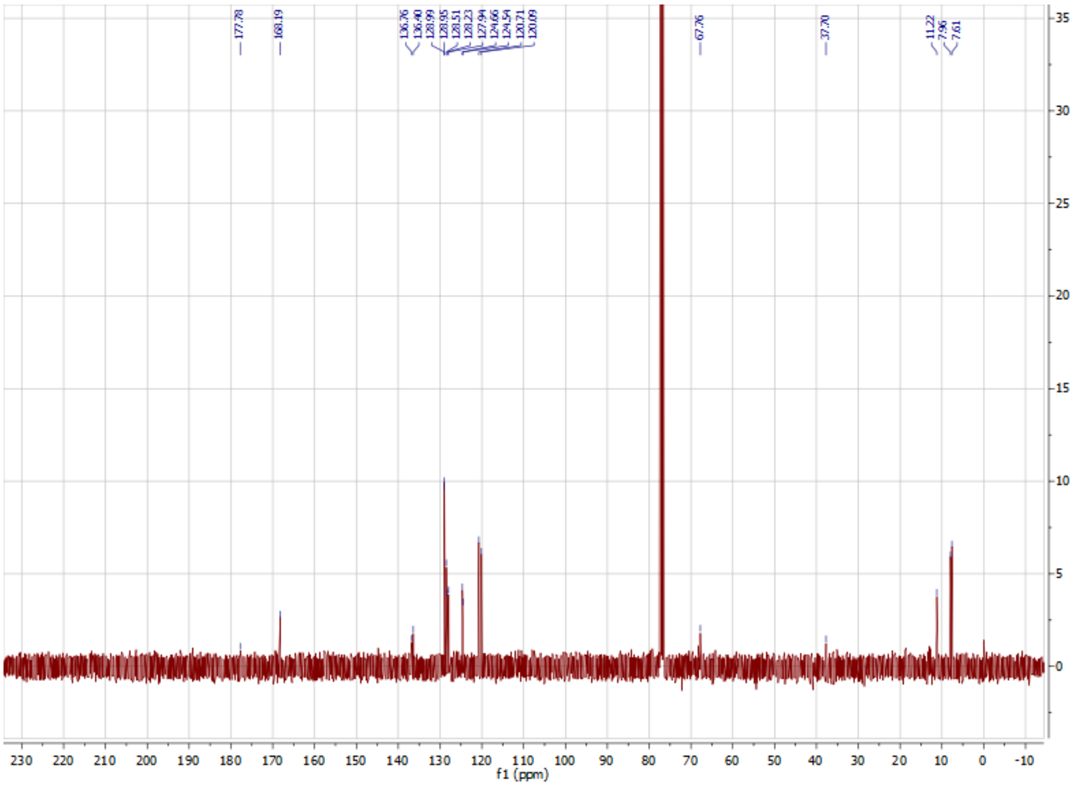
177.78
168.19
136.76
136.40
128.99
128.95
128.51
128.23
127.94
124.66
124.54
120.71
120.09
67.76
37.70
11.22
7.96
7.61
f1 (ppm)

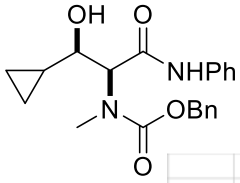

***syn*-benzyl-(1-cyclopropyl-1-hydroxy-3-oxo-3-(phenylamino)propan-2-yl)(methyl) carbamate, 28/29**

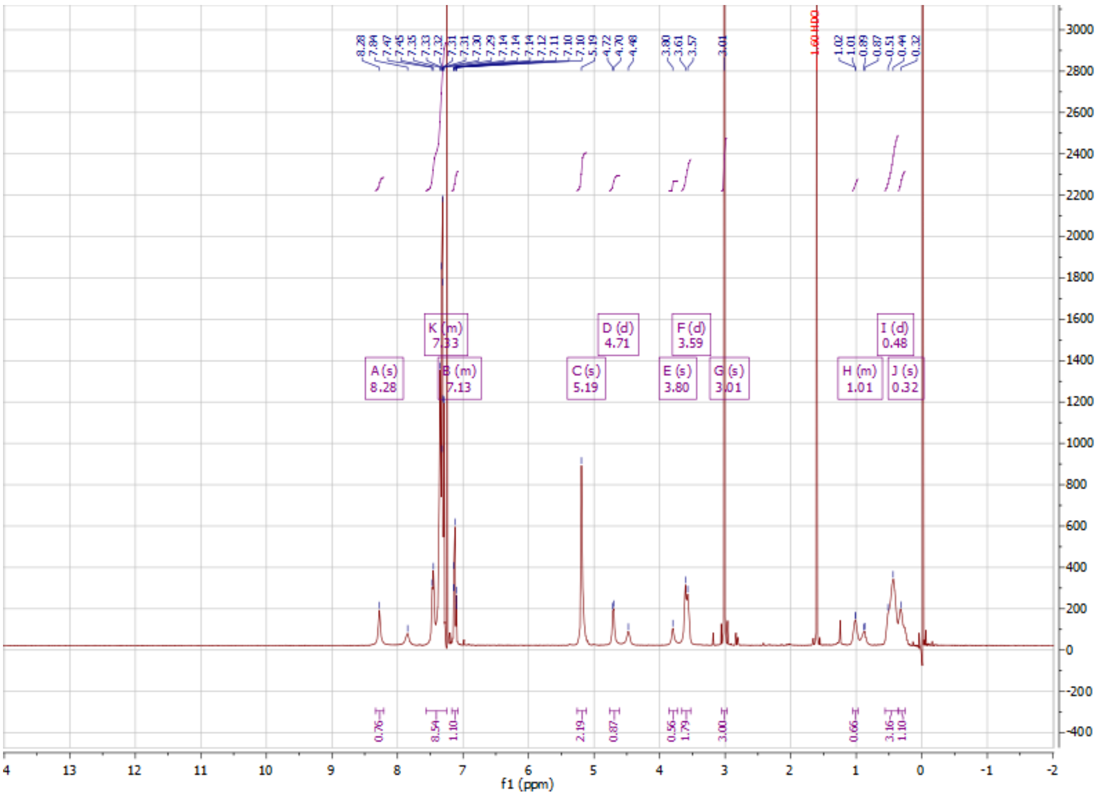

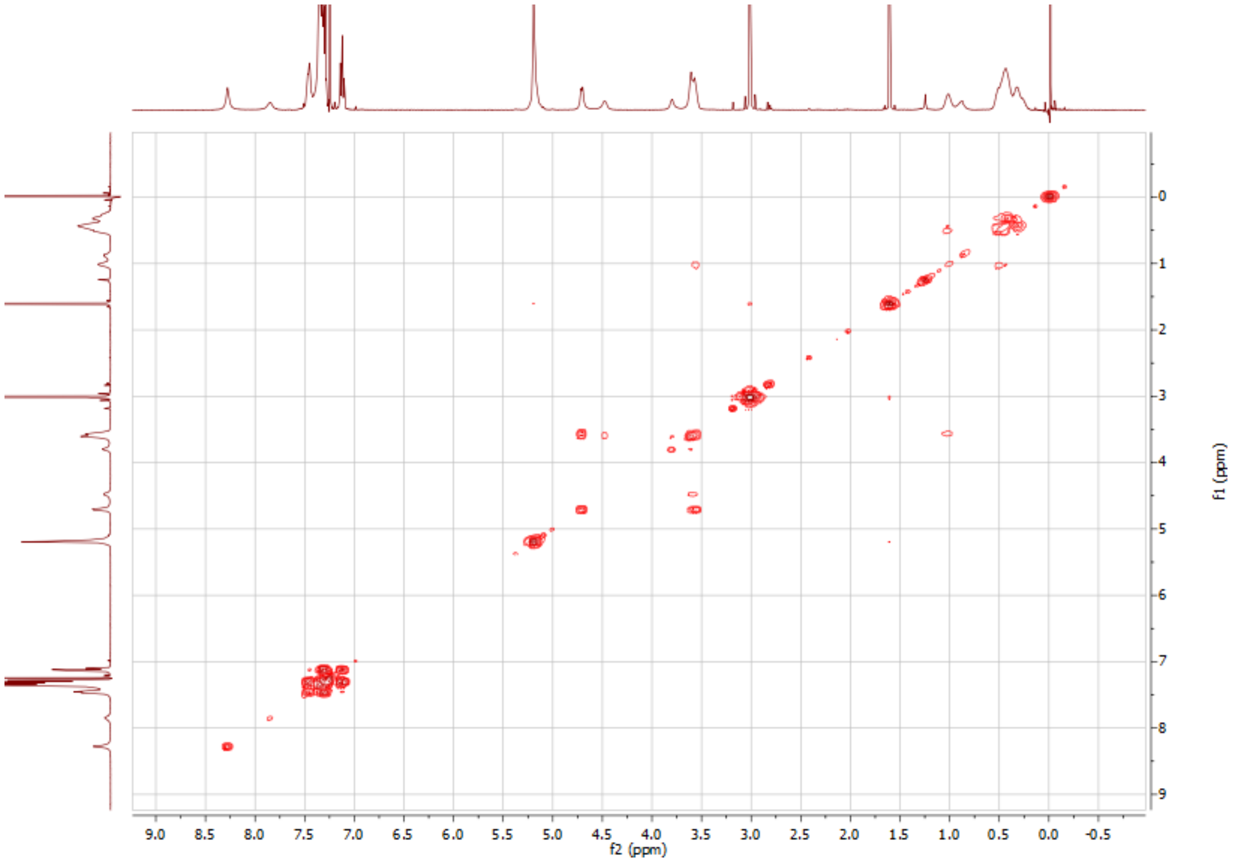

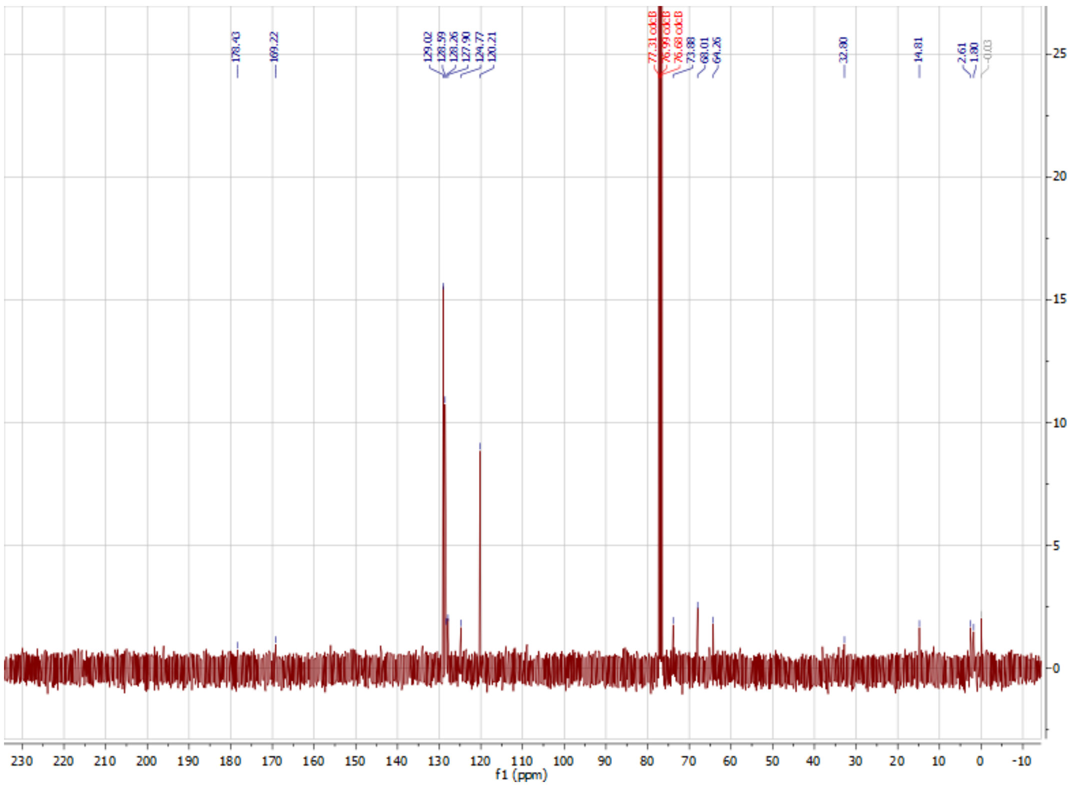

178.43
169.22
129.02
128.59
128.26
127.90
124.77
120.21
77.31 cdcl3
76.99 cdcl3
76.68 cdcl3
73.88
68.01
64.26
32.80
14.81
2.61
1.80
-0.03
f1 (ppm)

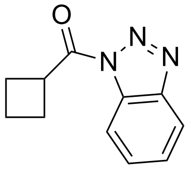

(1*H*-benzo[*d*][1,2,3]triazol-1-yl)(cyclobutyl)methanone

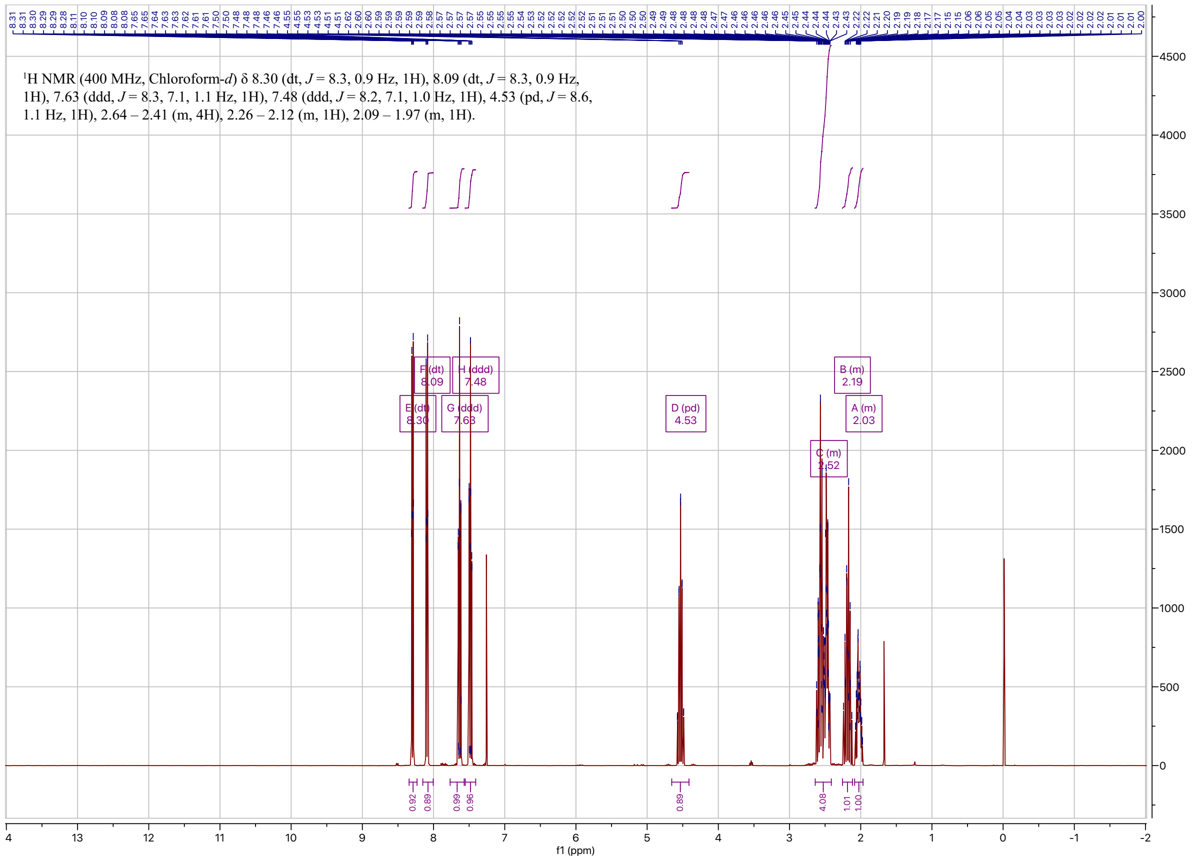

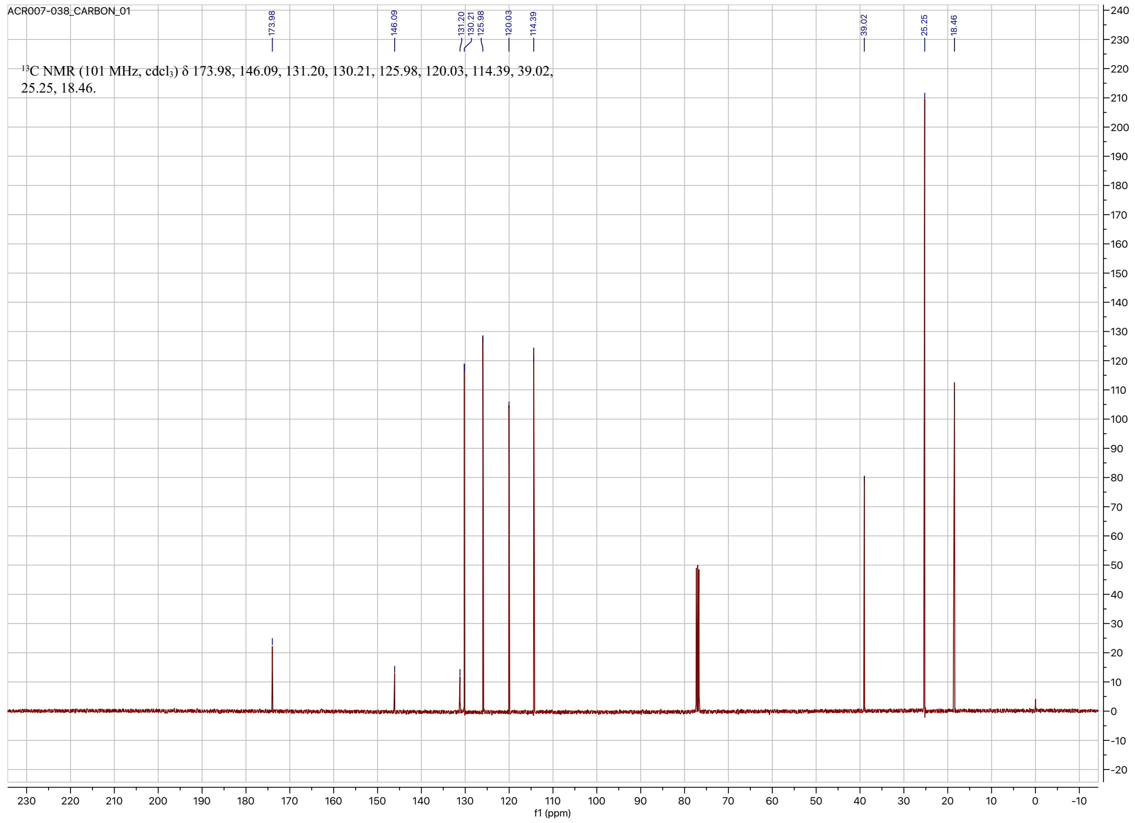

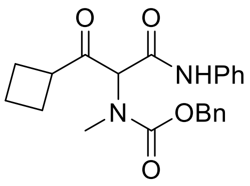

Benzyl (1-cyclobutyl-1,3-dioxo-3-(phenylamino)propan-2-yl)(methyl)carbamate

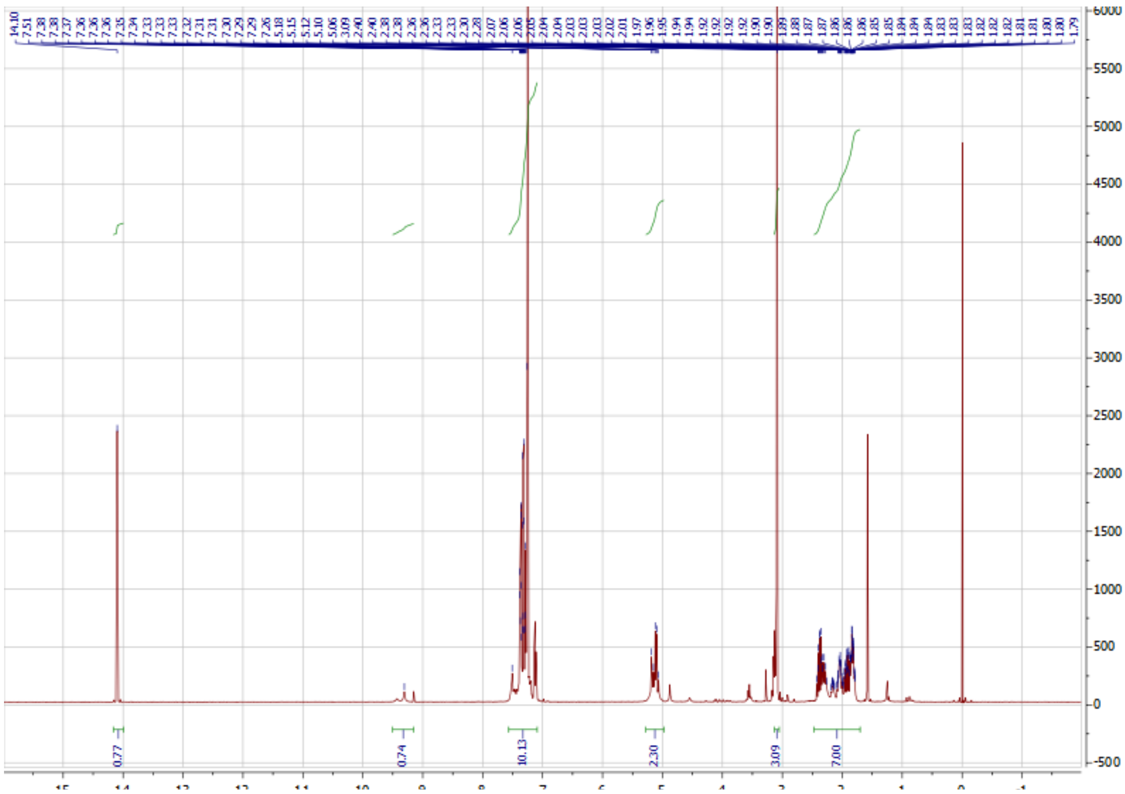

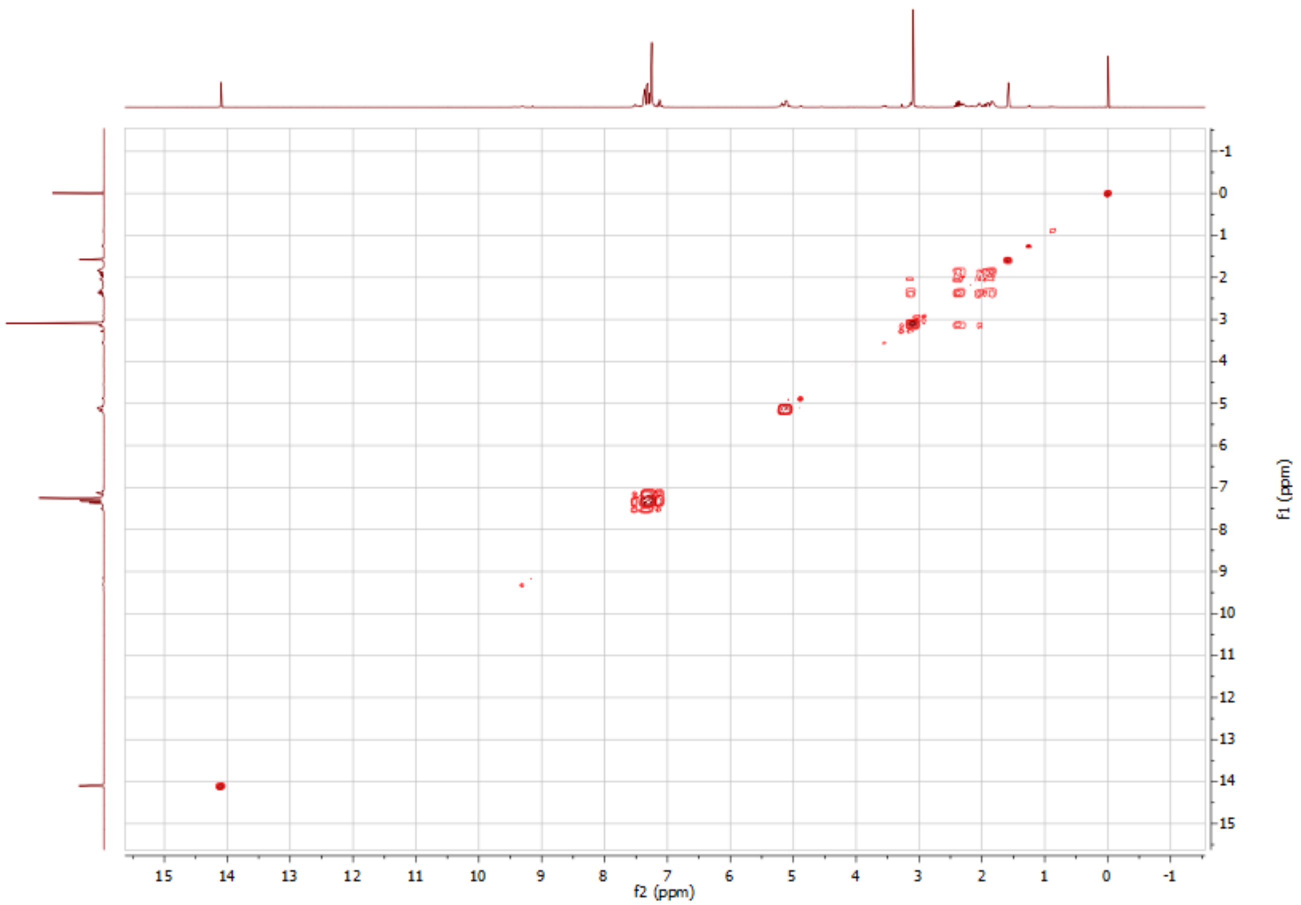

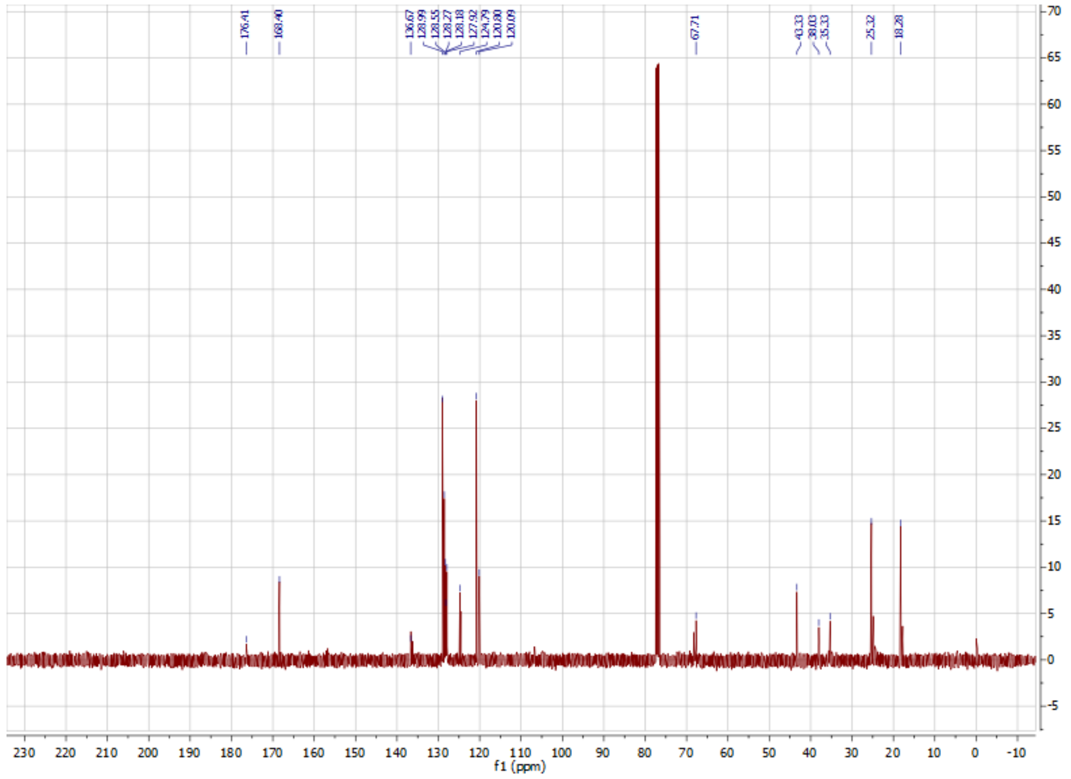
176.41
168.40
136.67
128.99
128.55
128.27
128.18
127.92
124.79
120.80
120.09
67.71
43.33
38.03
35.33
25.32
18.28
f1 (ppm)

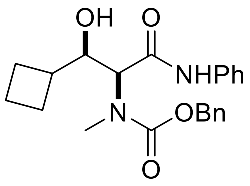

syn-Benzyl(-1-cyclobutyl-1-hydroxy-3-oxo-3-(phenylamino)propan-2-yl)(methyl) carbamate, 30/31

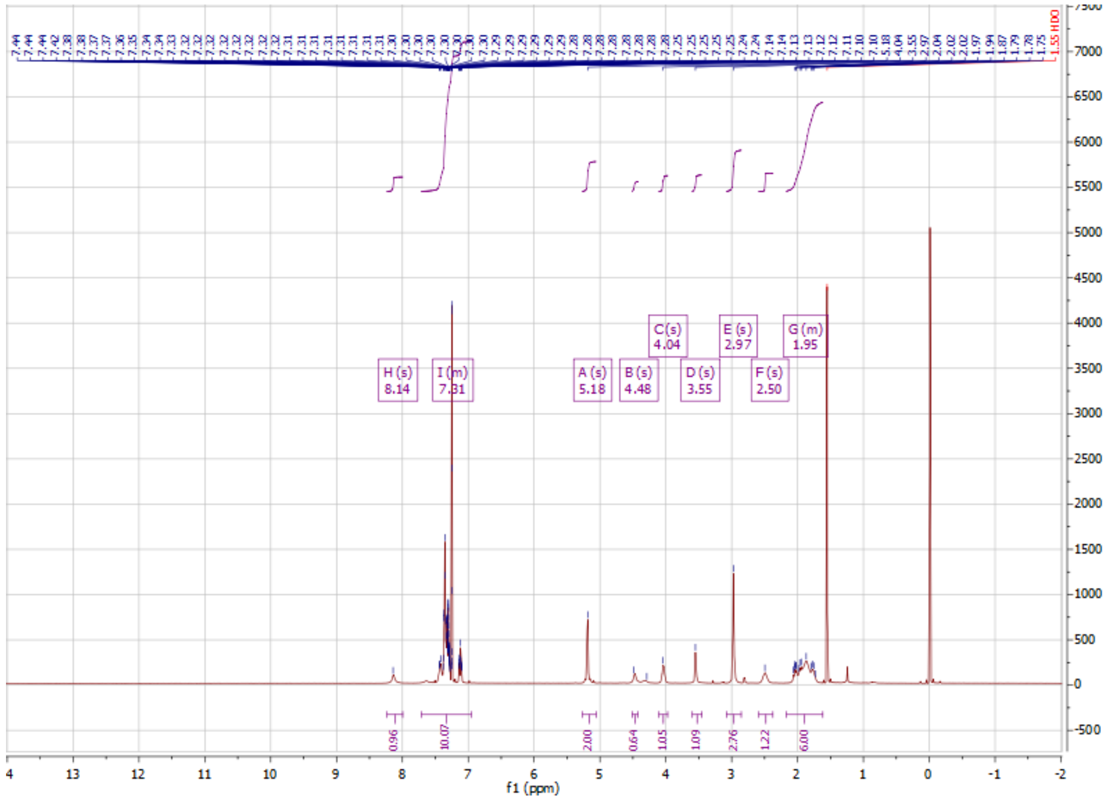

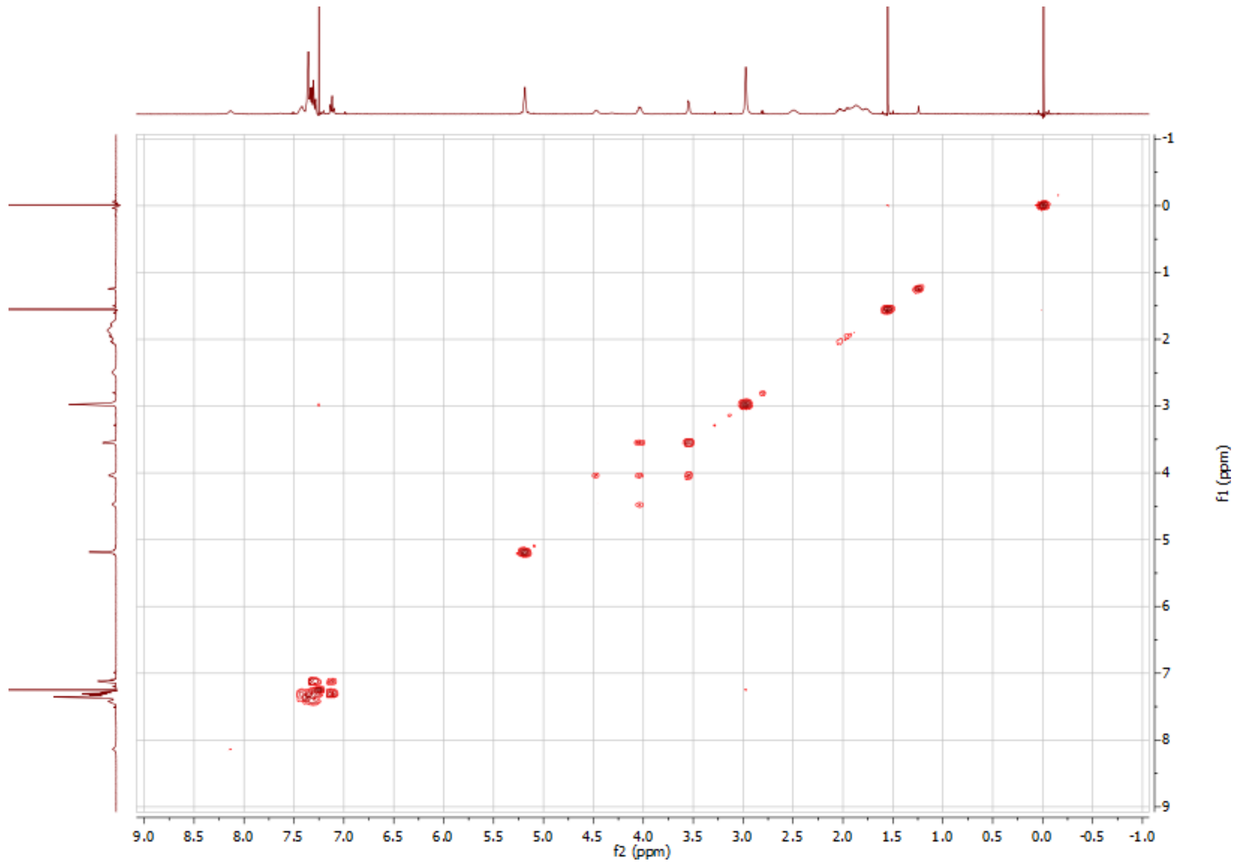

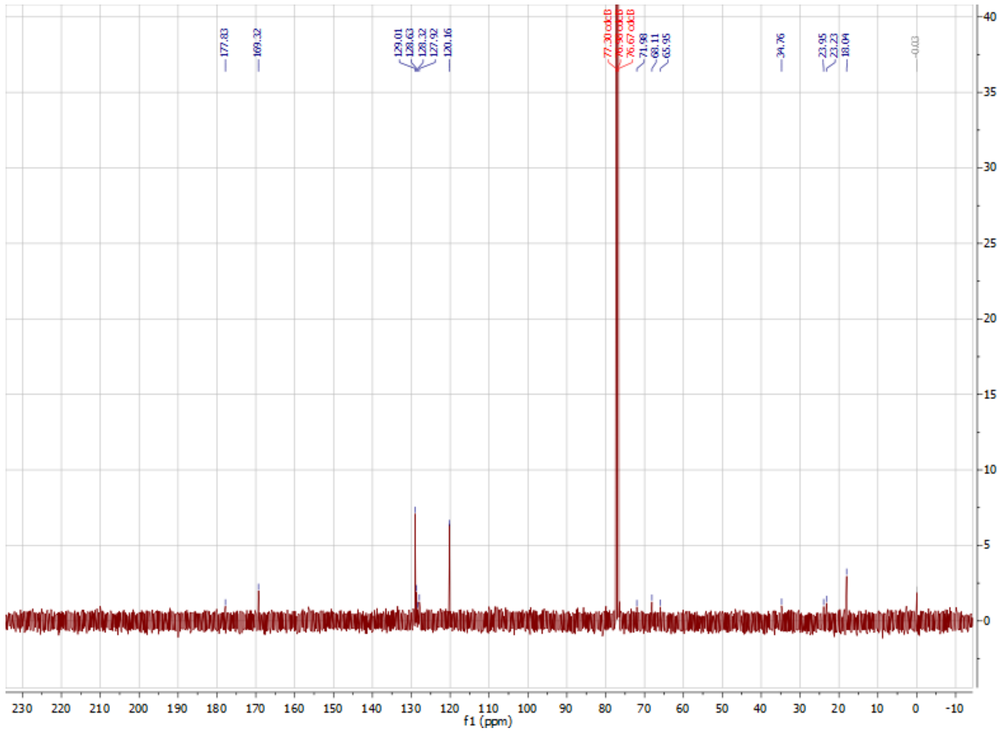

177.83
169.32
129.01
128.63
128.32
127.92
120.16
77.30 cdcl3
77.00 cdcl3
76.67 cdcl3
71.98
68.11
65.95
34.76
23.95
23.23
18.04
0.03
f1 (ppm)

Chiral analysis of *benzyl (3-hydroxy-4-methyl-1-oxo-1-(phenylamino)pentan-2-yl)(methyl)carbamate* **- 17**

Racemic sample was synthesized according to general procedures **C** and **E** utilizing isobutyraldehyde in place of 1-(1H-benzo[d][1,2,3]triazol-1-yl)-2-methylpropan-1-one, yielding a mixture of two diastereomers. Racemic chiral HPLC sample was taken from a chromatography fraction that appeared to have approximate equal quantities of both diastereomers as judged by TLC. A purified sample of diastereomer **A** (later identified as *anti* after comparison to X-ray sample) was able to be obtained by column chromatography. Diastereomer B was identical to samples obtained by ATH DKR of (**17**) by NMR.

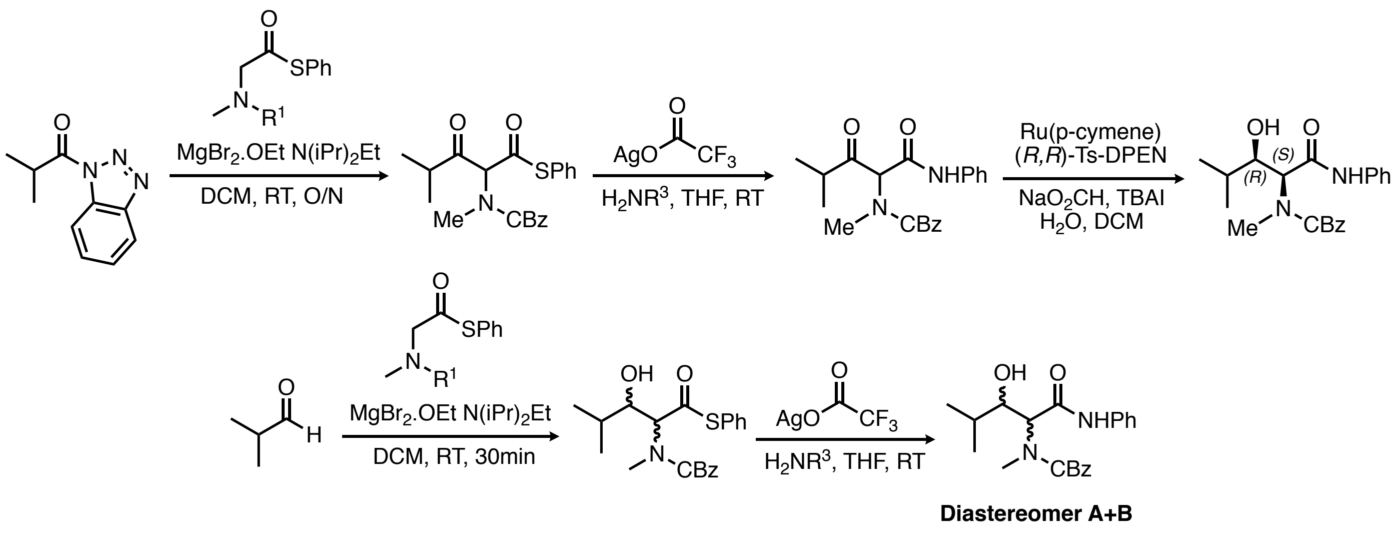

Racemic benzyl (3-hydroxy-4-methyl-1-oxo-1-(phenylamino)pentan-2-yl)(methyl)carbamate - **17**

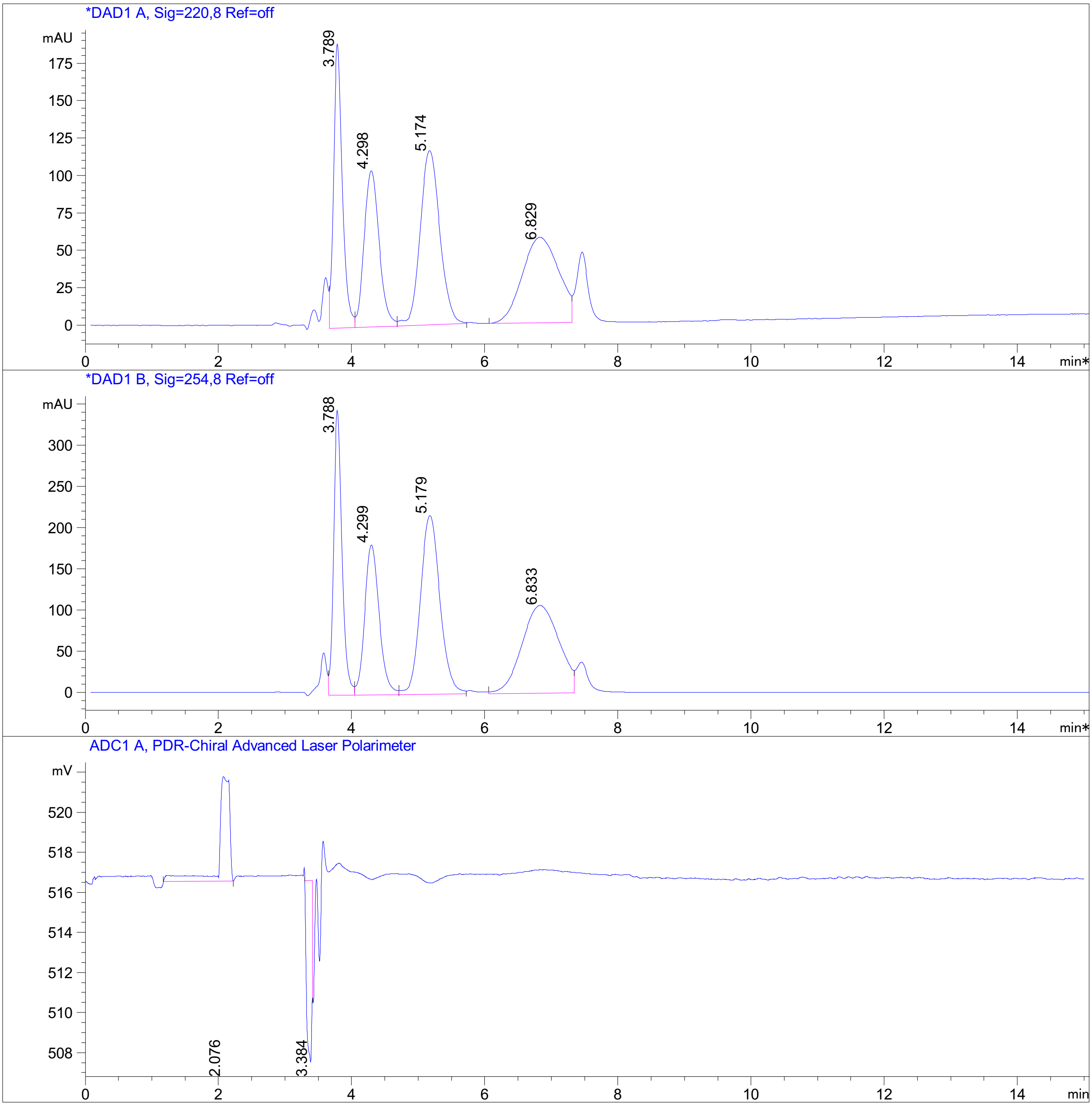

benzyl (3-hydroxy-4-methyl-1-oxo-1-(phenylamino)pentan-2-yl)(methyl)carbamate – **17 Diastereomer A (anti).**

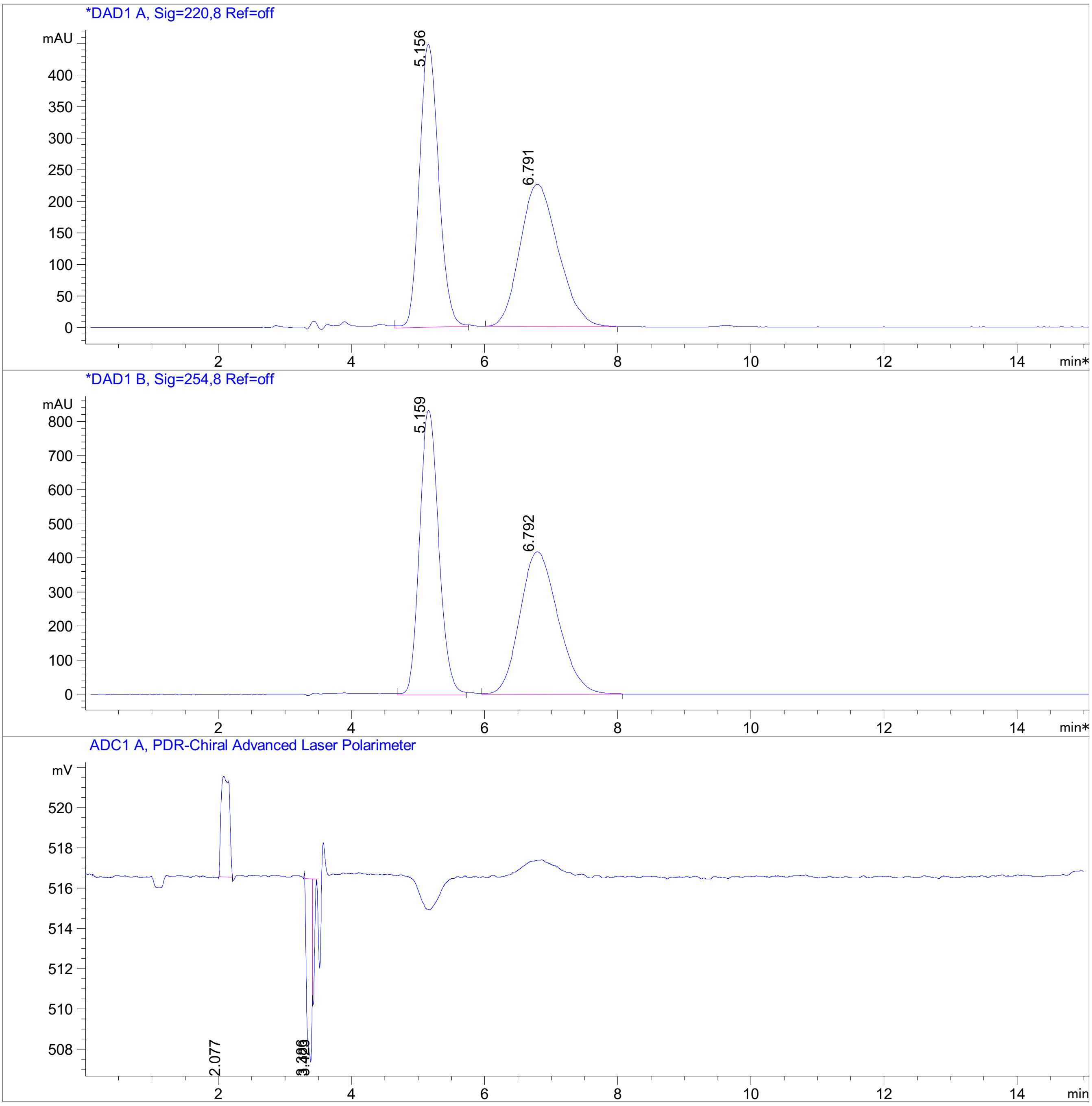

NMR Diastereomer analysis

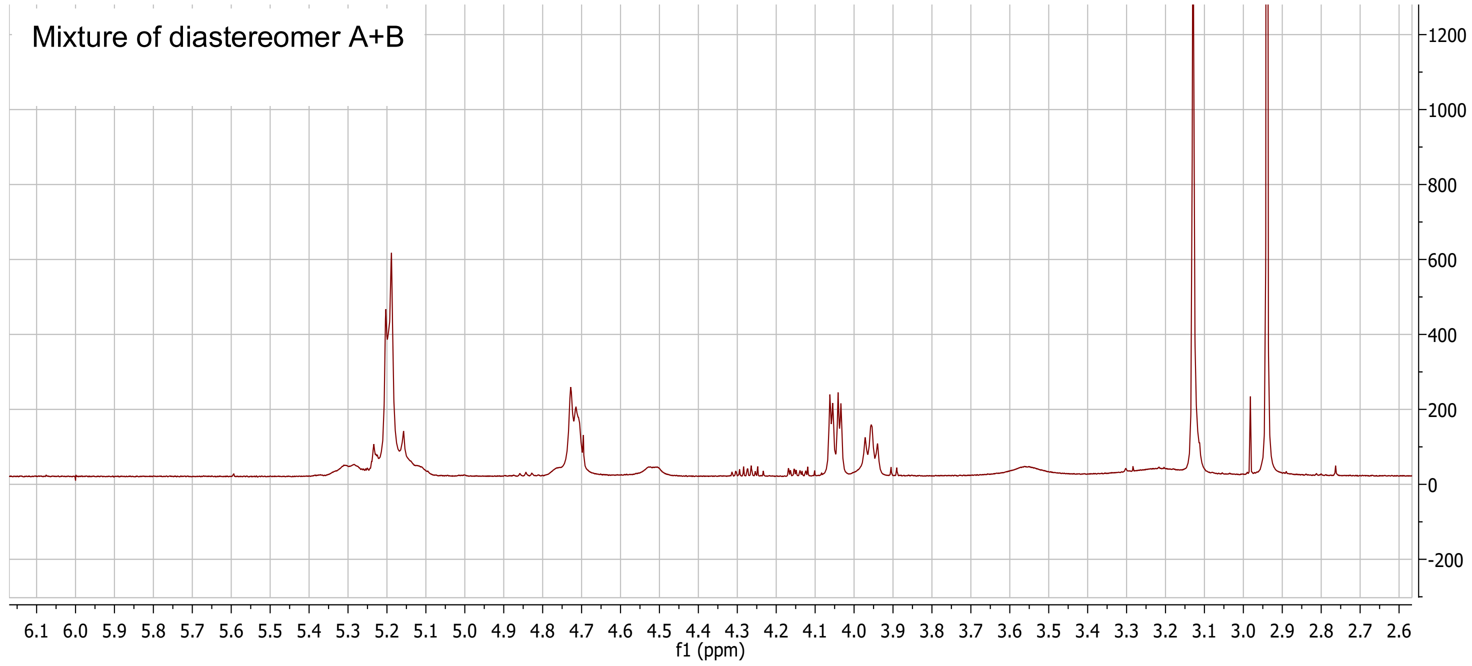

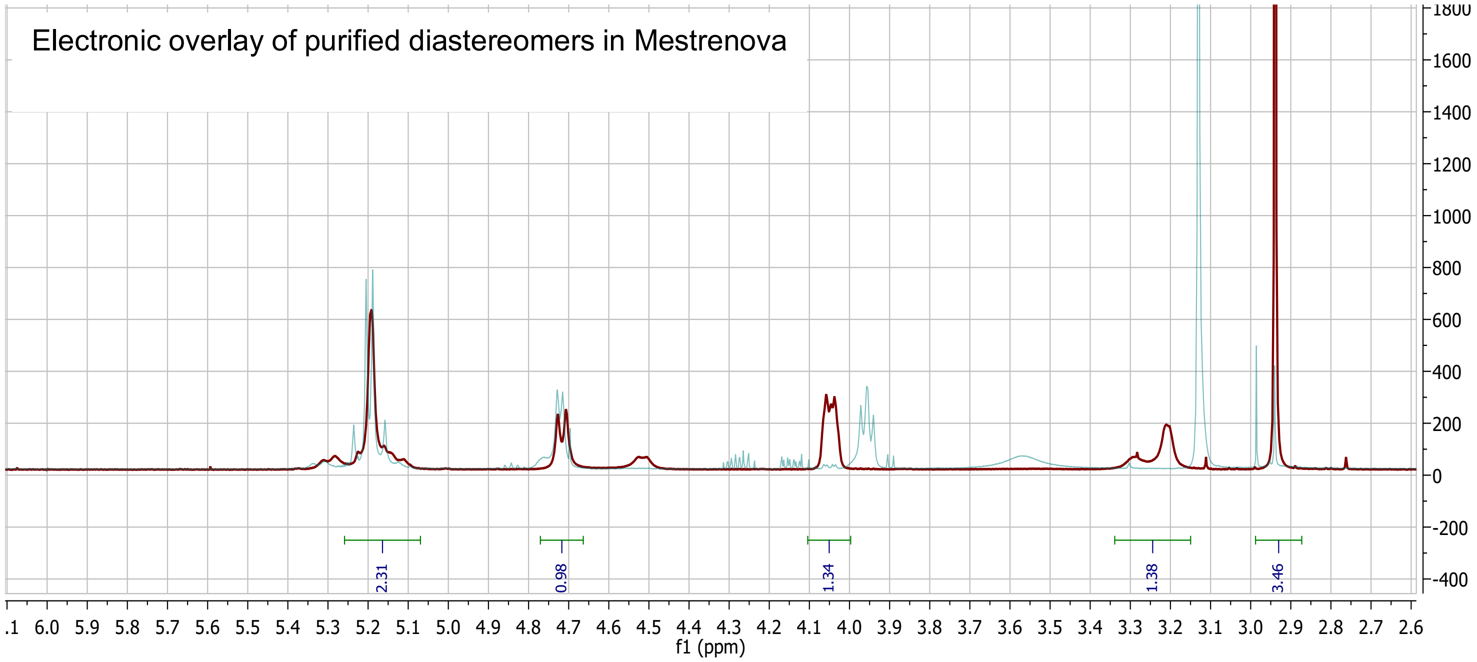

Chiral HPLC chromatograms from Table S1

S1. Entry 1 – Cat. A. Ru(p-cymene)[(R,R)-Ts-DPEN]

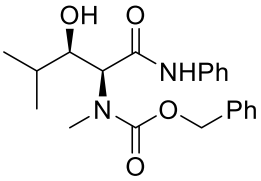

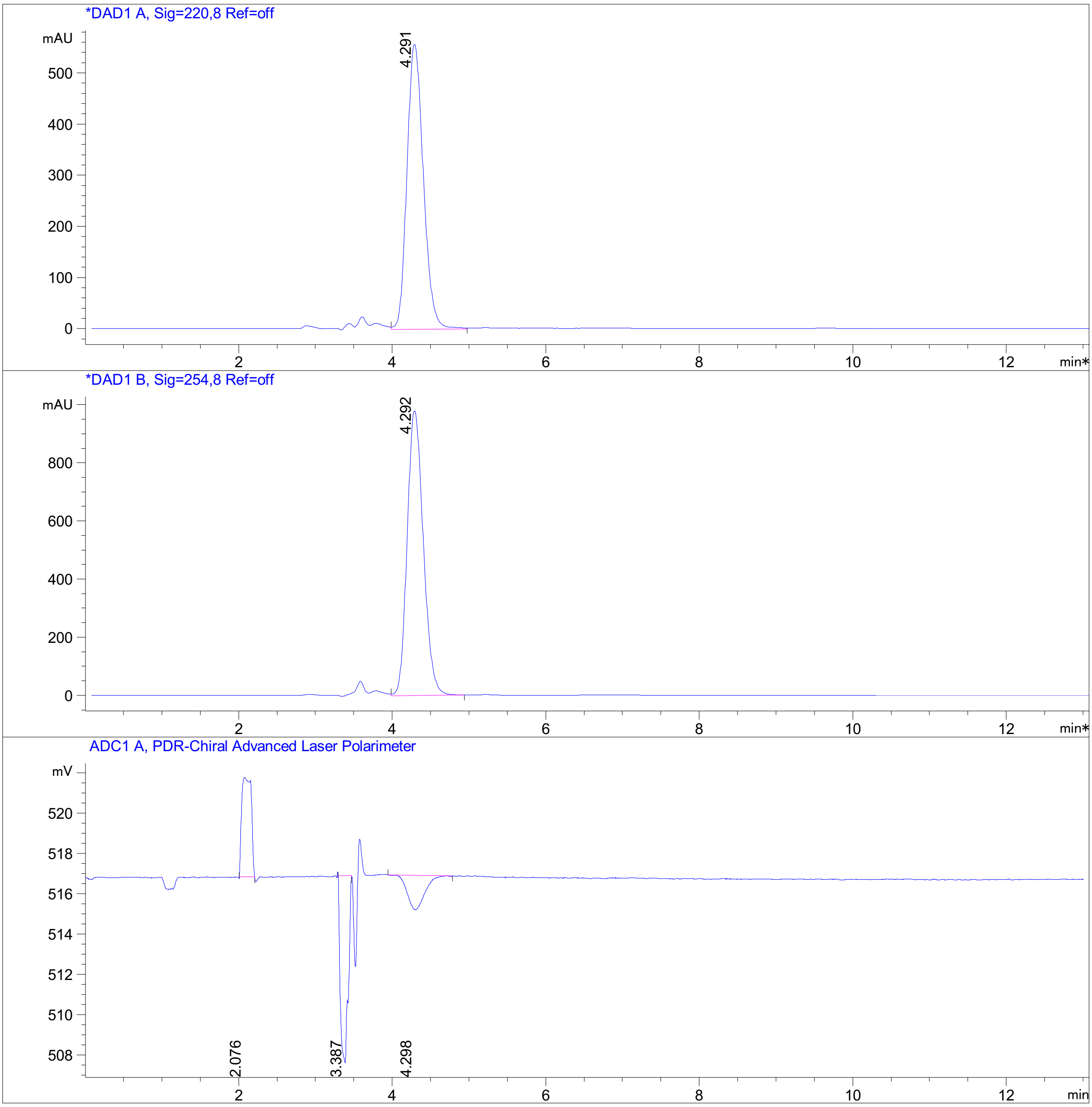

S1. Entry 2 – Cat B. Ru[(R,R)-TsDPEN](mesitylene)

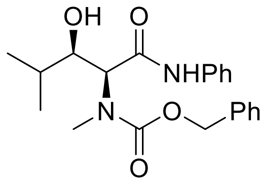

Additional Info : Peak(s) manually integrated

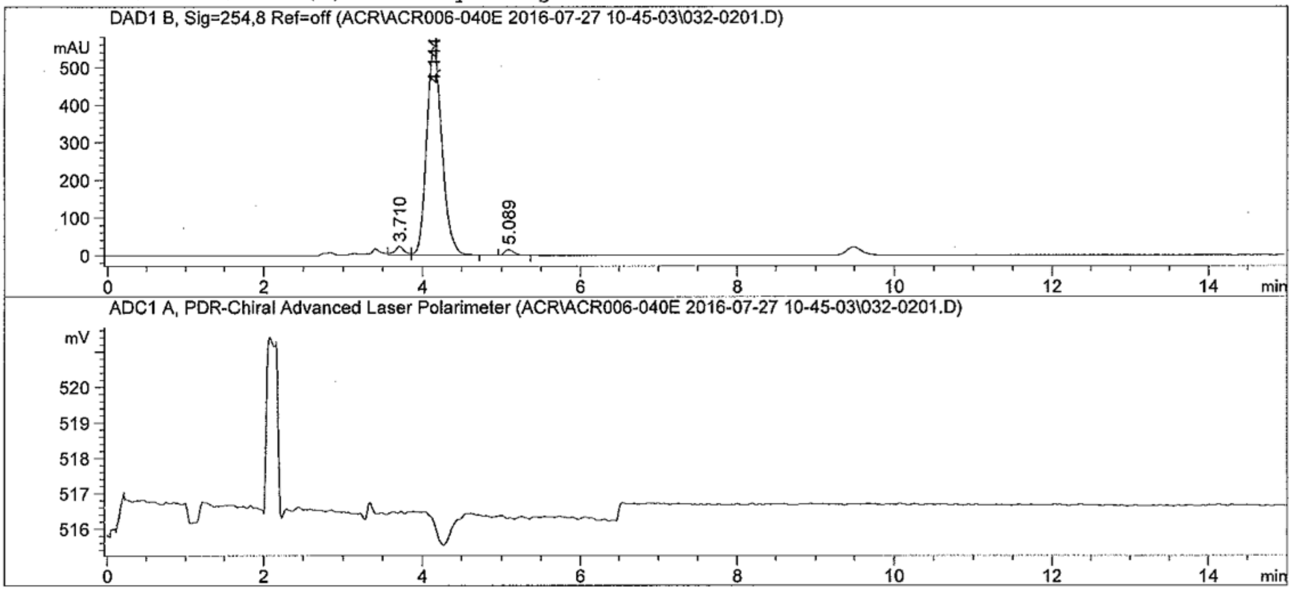

==

Area Percent Report

==

Sorted By : Signal
Multiplier : 1.0000
Dilution : 1.0000
Do not use Multiplier & Dilution Factor with ISTDs

Signal 1: DAD1 B, Sig=254,8 Ref=off

Peak #	RetTime [min]	Type	Width [min]	Area [mAU*s]	Height [mAU]	Area %
1	3.710	VV	0.1229	210.01619	23.81942	2.6470
2	4.144	VB	0.2128	7609.24658	552.01428	95.9070
3	5.089	BB	0.1233	114.71864	14.33808	1.4459

Totals : 7933.98141 590.17178

S1. Entry 3 – Cat C. Ru(p-cymene)[(R,R)-Fs-DPEN]

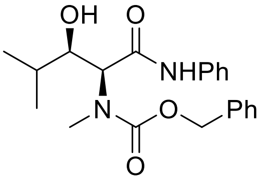

Additional Info : Peak(s) manually integrated

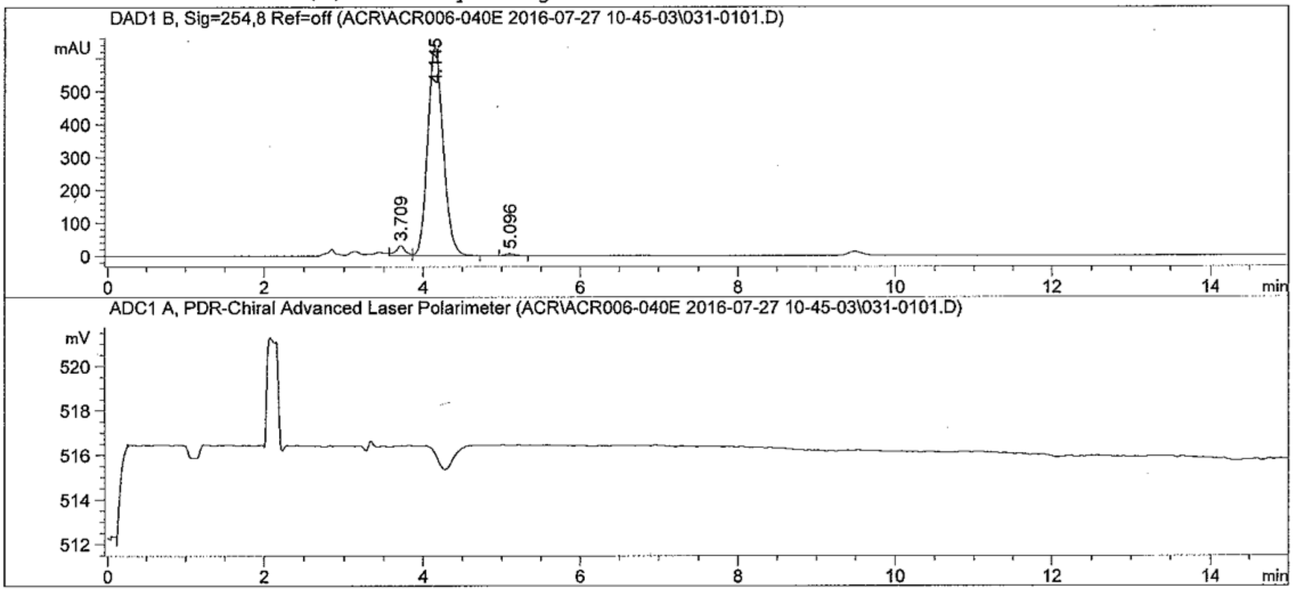

===

Area Percent Report

===

Sorted By : Signal
Multiplier : 1.0000
Dilution : 1.0000
Do not use Multiplier & Dilution Factor with ISTDs

Signal 1: DAD1 B, Sig=254,8 Ref=off

Peak #	RetTime [min]	Type	Width [min]	Area [mAU*s]	Height [mAU]	Area %
1	3.709	VV	0.1241	268.12659	30.65196	2.9646
2	4.145	VB	0.2150	8724.49609	632.09247	96.4654
3	5.096	BB	0.1288	51.54995	6.21606	0.5700

Totals : 9044.17263 668.96049

S1. Entry 4 – Cat D.

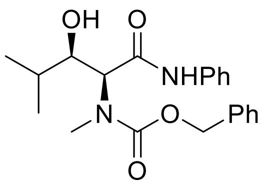

Additional Info : Peak(s) manually integrated

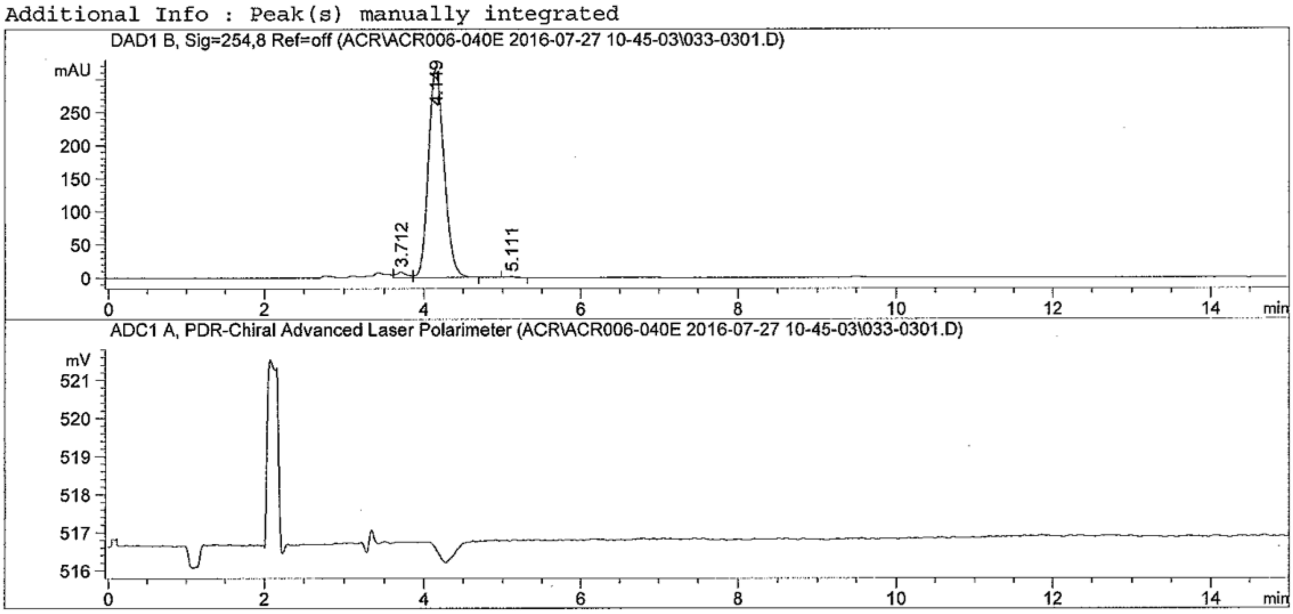

===

Area Percent Report

===

Sorted By : Signal
Multiplier : 1.0000
Dilution : 1.0000
Do not use Multiplier & Dilution Factor with ISTDs

Signal 1: DAD1 B, Sig=254,8 Ref=off

Peak #	RetTime [min]	Type	Width [min]	Area [mAU*s]	Height [mAU]	Area %
1	3.712	VV	0.1346	81.20387	8.41102	1.8248
2	4.149	VB	0.2162	4356.22412	313.41925	97.8943
3	5.111	BB	0.1279	12.49660	1.46000	0.2808

Totals : 4449.92460 323.29027

S1. Entry 5 – Cat E.

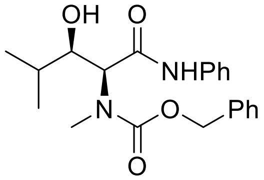

Additional Info : Peak(s) manually integrated

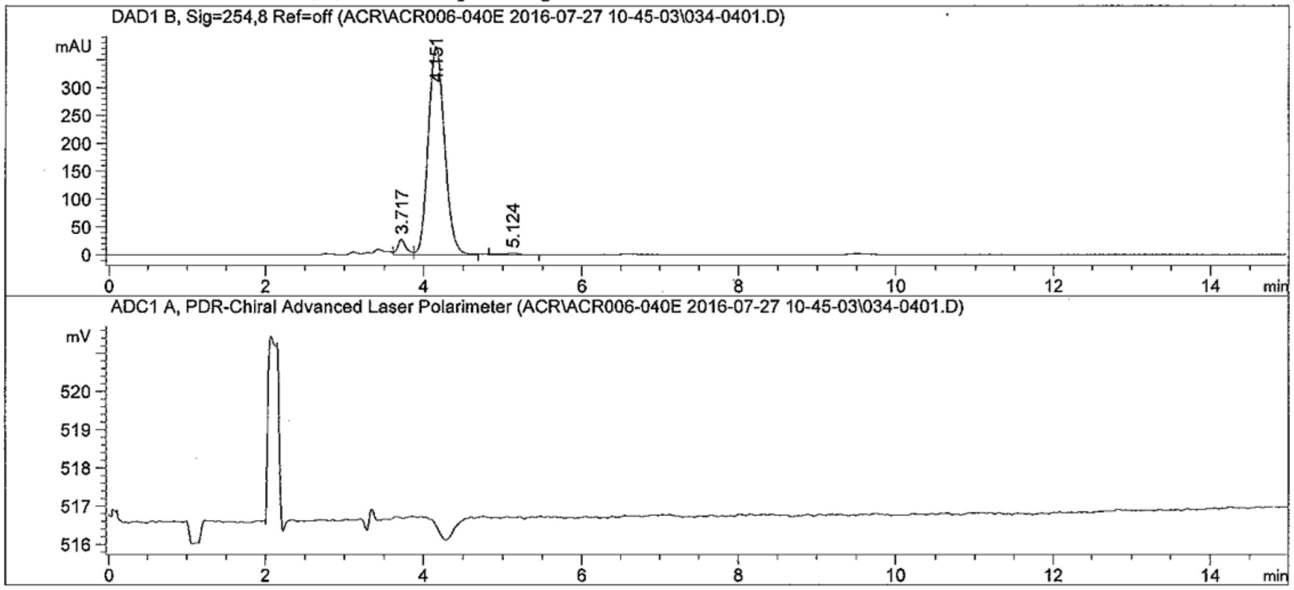

===

Area Percent Report

===

Sorted By : Signal
Multiplier : 1.0000
Dilution : 1.0000
Do not use Multiplier & Dilution Factor with ISTDs

Signal 1: DAD1 B, Sig=254,8 Ref=off

Peak #	RetTime [min]	Type	Width [min]	Area [mAU*s]	Height [mAU]	Area %
1	3.717	VV	0.1101	213.86124	28.33149	3.8773
2	4.151	VB	0.2182	5256.19922	373.53464	95.2949
3	5.124	BB	0.1911	45.66090	3.26519	0.8278

Totals : 5515.72135 405.13132

S1. Entry 6 – Cat F.

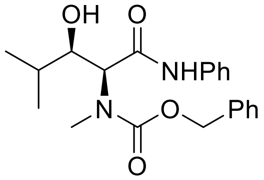

Additional Info : Peak(s) manually integrated

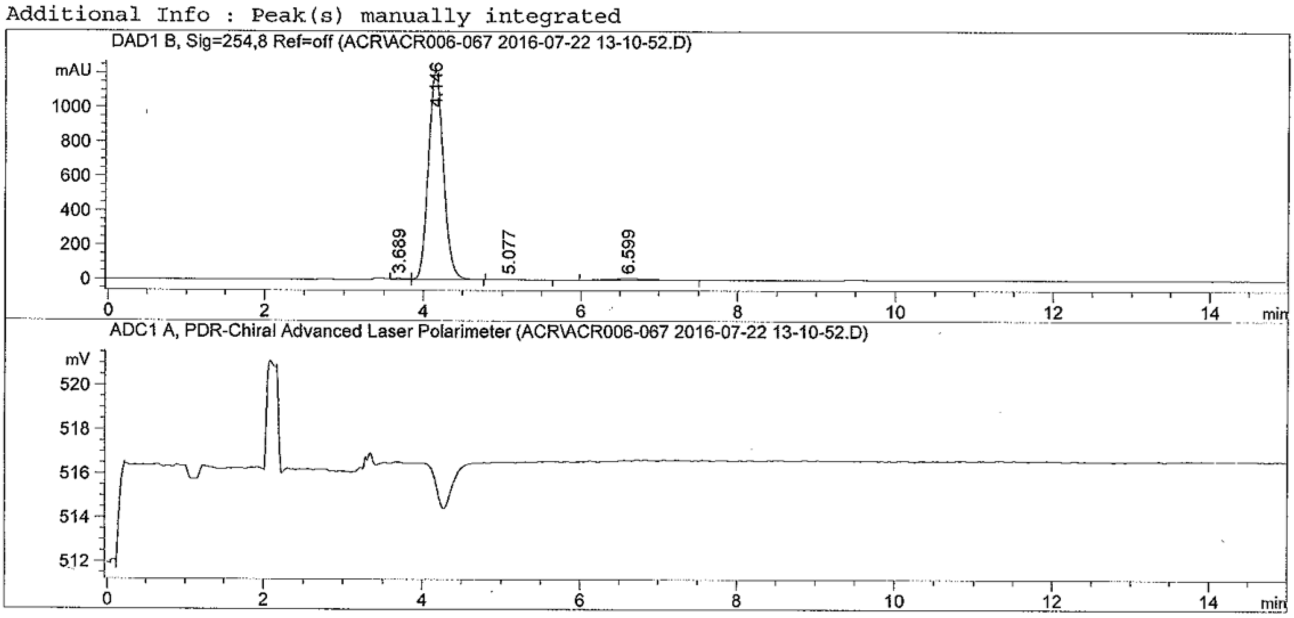

===

Area Percent Report

===

Sorted By : Signal
Multiplier : 1.0000
Dilution : 1.0000
Sample Amount: : 10.00000 [ng/ul] (not used in calc.)
Do not use Multiplier & Dilution Factor with ISTDs

Signal 1: DAD1 B, Sig=254,8 Ref=off

Peak #	RetTime [min]	Type	Width [min]	Area [mAU*s]	Height [mAU]	Area %
1	3.689	VV	0.1759	90.92557	7.17465	0.5394
2	4.146	VB	0.2124	1.64092e4	1208.93835	97.3400
3	5.077	BB	0.2405	68.40959	3.79225	0.4058
4	6.599	BB	0.4382	289.07190	8.10368	1.7148

Benzyl((2S,3R)-3-hydroxy-4-methyl-1-oxo-1-(phenylamino)pentan-2-yl)(methyl)carbamate, 17

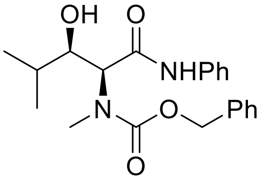

Additional Info : Peak(s) manually integrated

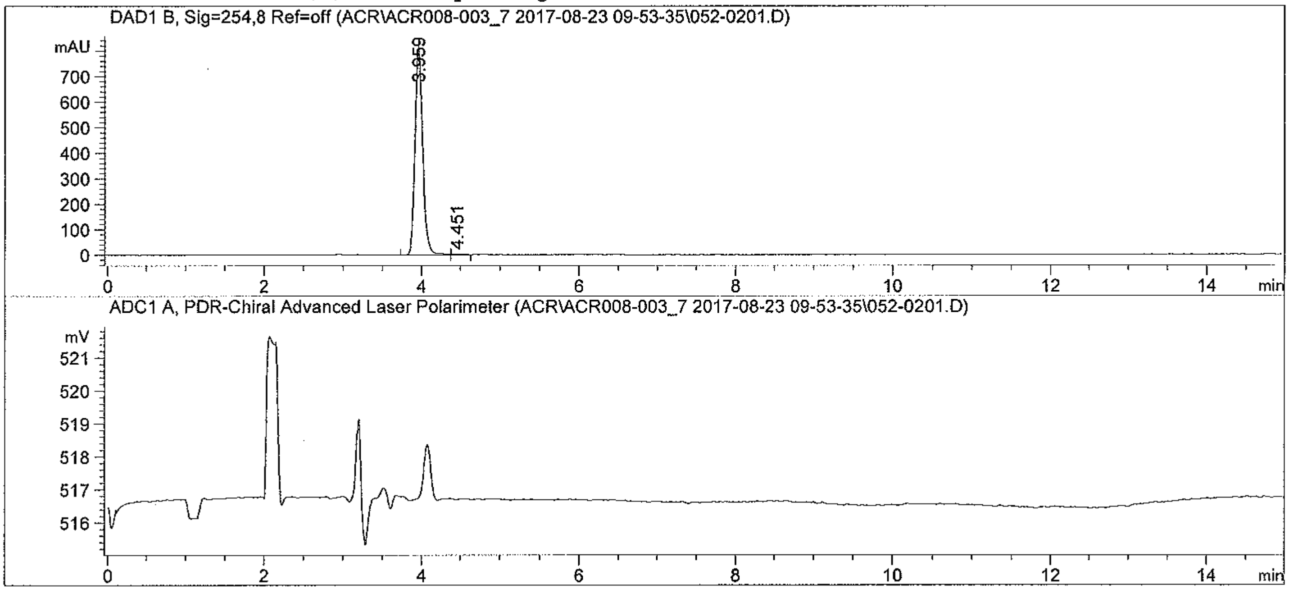

===

Area Percent Report

===

Sorted By : Signal
Multiplier : 1.0000
Dilution : 1.0000
Do not use Multiplier & Dilution Factor with ISTDs

Signal 1: DAD1 B, Sig=254,8 Ref=off

Peak #	RetTime [min]	Type	Width [min]	Area [mAU*s]	Height [mAU]	Area %
1	3.959	VV	0.1052	5546.01025	816.80585	99.2462
2	4.451	VV	0.1567	42.12186	3.35081	0.7538

Totals : 5588.13211 820.15665

Benzyl ((2R,3S)-3-hydroxy-4-methyl-1-oxo-1-(phenylamino)pentan-2-yl)(methyl)carbamate, 26

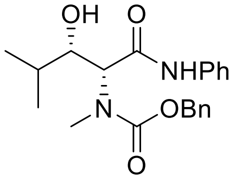

Additional Info : Peak(s) manually integrated

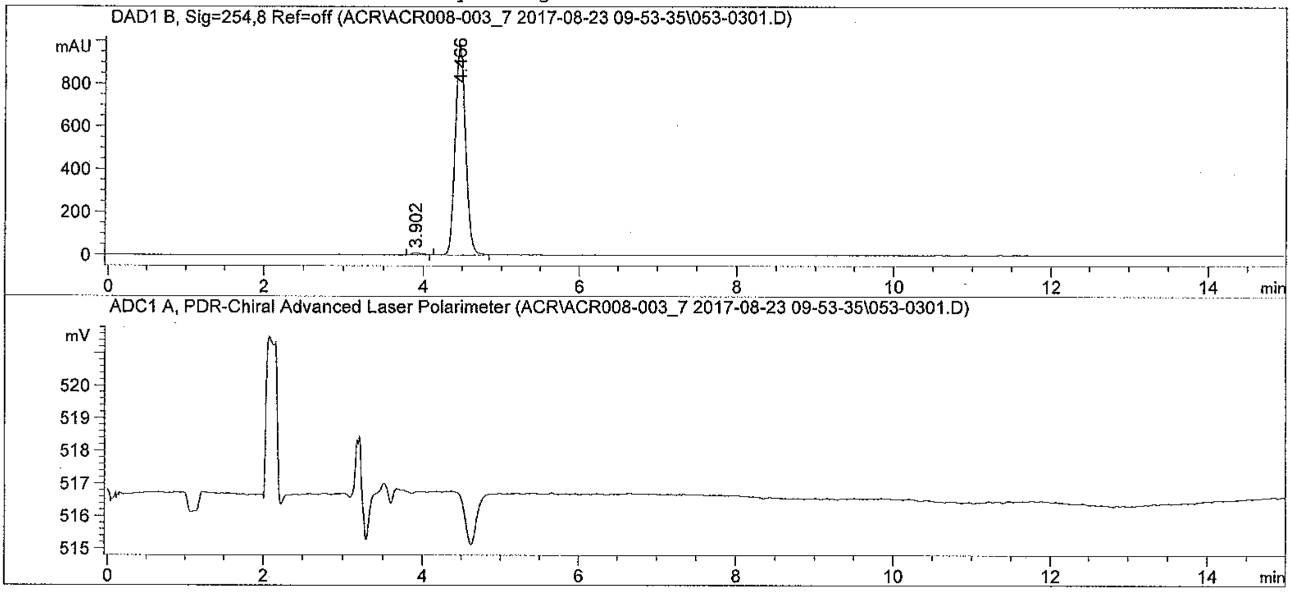

==

Area Percent Report

==

Sorted By : Signal
Multiplier : 1.0000
Dilution : 1.0000
Do not use Multiplier & Dilution Factor with ISTDs

Signal 1: DAD1 B, Sig=254,8 Ref=off

Peak #	RetTime [min]	Type	Width [min]	Area [mAU*s]	Height [mAU]	Area %
1	3.902	VV	0.1294	102.07346	10.68969	1.1223
2	4.466	VB	0.1441	8992.65723	970.45032	98.8777

Totals : 9094.73068 981.14001

Benzyl ((1S,2R)-1-cyclopropyl-1-hydroxy-3-oxo-3-(phenylamino)propan-2-yl)(methyl)carbamate, 29

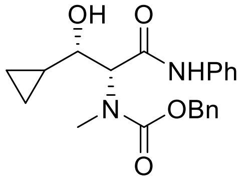

Additional Info : Peak(s) manually integrated

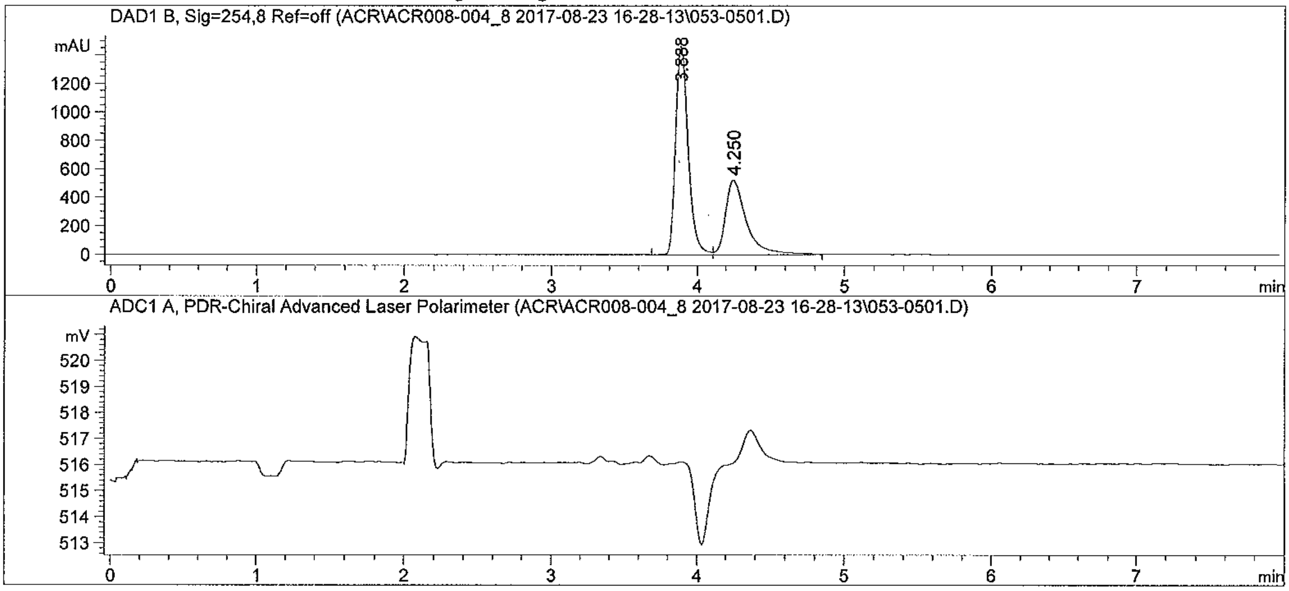

===

Area Percent Report

===

Sorted By : Signal
Multiplier : 1.0000
Dilution : 1.0000
Do not use Multiplier & Dilution Factor with ISTDs

Signal 1: DAD1 B, Sig=254,8 Ref=off

Peak #	RetTime [min]	Type	Width [min]	Area [mAU*s]	Height [mAU]	Area %
1	3.888	VV	0.0935	8717.10742	1463.34253	62.9210
2	4.250	VV	0.1445	5136.93750	523.57275	37.0790

Totals : 1.38540e4 1986.91528

Benzyl ((1R,2S)-1-cyclopropyl-1-hydroxy-3-oxo-3-(phenylamino)propan-2-yl)(methyl)carbamate, 28

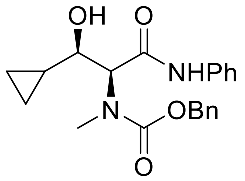

Additional Info : Peak(s) manually integrated

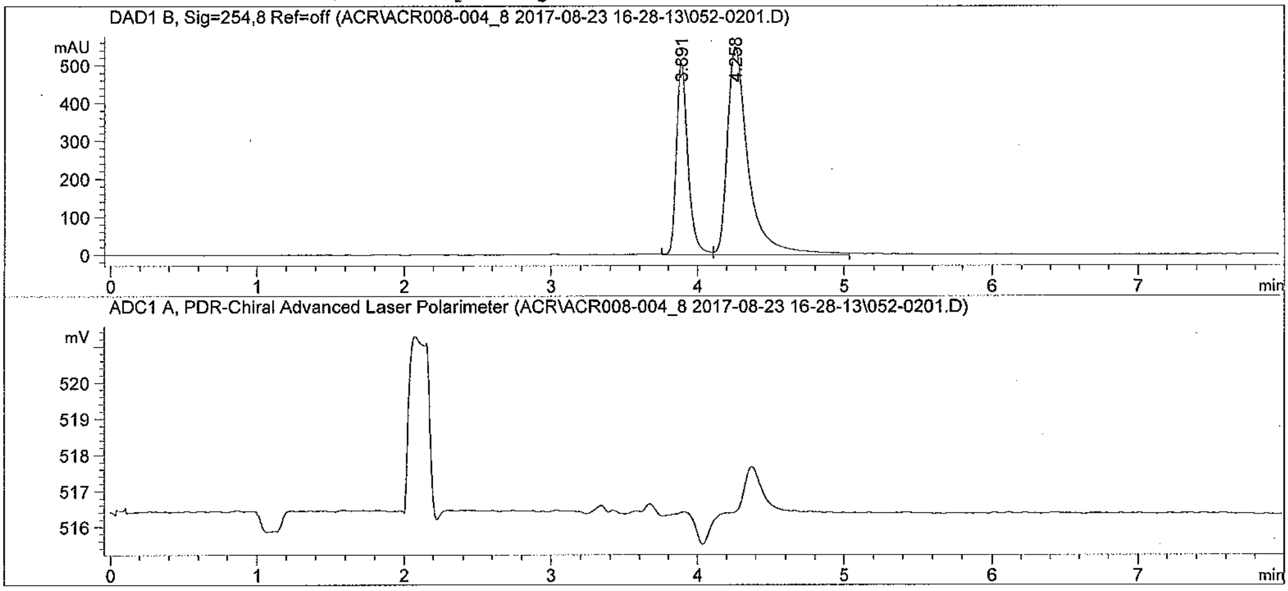

===

Area Percent Report

===

Sorted By : Signal
Multiplier : 1.0000
Dilution : 1.0000
Do not use Multiplier & Dilution Factor with ISTDs

Signal 1: DAD1 B, Sig=254,8 Ref=off

Peak #	RetTime [min]	Type	Width [min]	Area [mAU*s]	Height [mAU]	Area %
1	3.891	VV	0.0827	2811.68677	506.72009	34.1612
2	4.258	VB	0.1449	5418.94971	550.25854	65.8388

Totals : 8230.63647 1056.97864

Benzyl ((1S,2R)-1-cyclobutyl-1-hydroxy-3-oxo-3-(phenylamino)propan-2-yl)(methyl)carbamate, 31

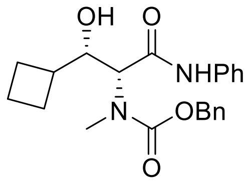

Additional Info : Peak(s) manually integrated

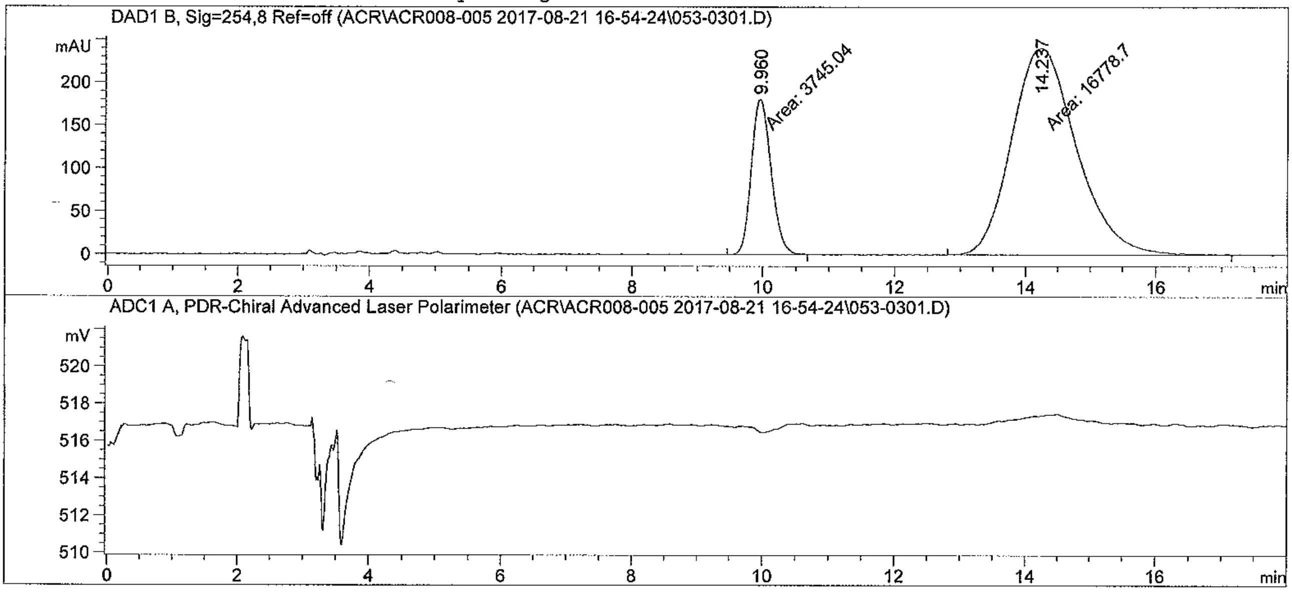

Area Percent Report

Sorted By : Signal
Multiplier : 1.0000
Dilution : 1.0000
Do not use Multiplier & Dilution Factor with ISTDs

Signal 1: DAD1 B, Sig=254,8 Ref=off

Peak #	RetTime [min]	Type	Width [min]	Area [mAU*s]	Height [mAU]	Area %
1	9.960	MM	0.3452	3745.04492	180.81860	18.2473
2	14.237	MM	1.1620	1.67787e4	240.65768	81.7527
Totals :				2.05238e4	421.47629	

Benzyl ((1R,2S)-1-cyclobutyl-1-hydroxy-3-oxo-3-(phenylamino)propan-2-yl)(methyl)carbamate, 30

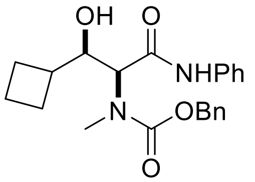

Additional Info : Peak(s) manually integrated

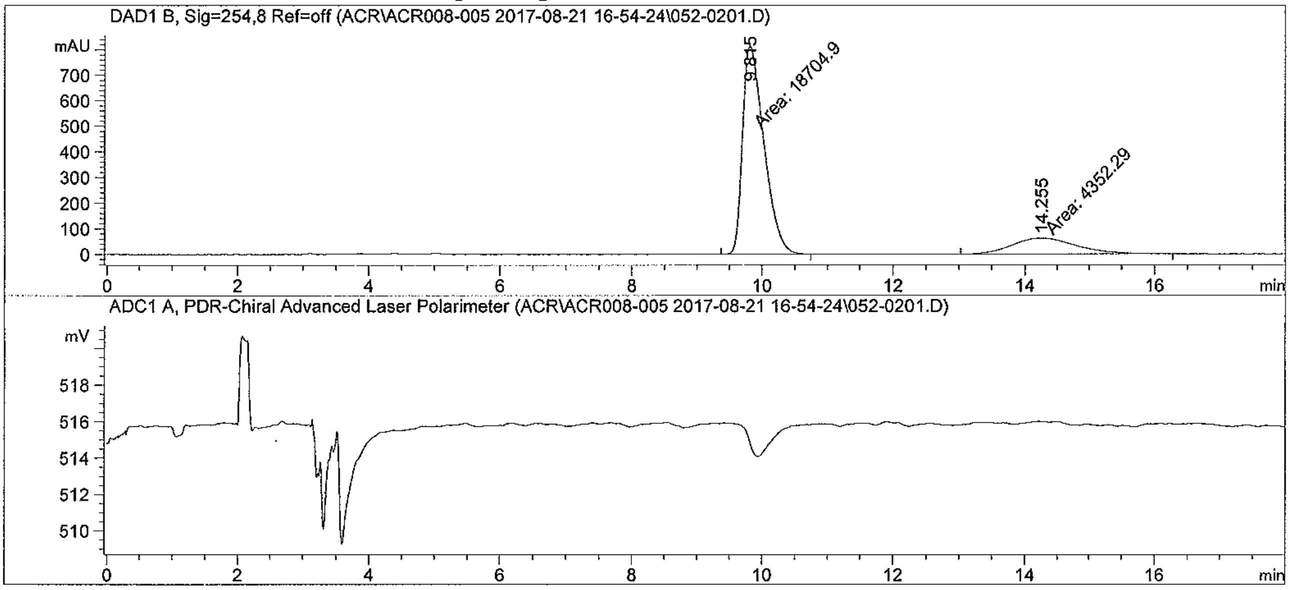

===

Area Percent Report

===

Sorted By : Signal
Multiplier : 1.0000
Dilution : 1.0000
Do not use Multiplier & Dilution Factor with ISTDs

Signal 1: DAD1 B, Sig=254,8 Ref=off

Peak #	RetTime [min]	Type	Width [min]	Area [mAU*s]	Height [mAU]	Area %
1	9.815	MM	0.3827	1.87049e4	814.61737	81.1239
2	14.255	MM	1.1618	4352.29053	62.43803	18.8761

Totals : 2.30571e4 877.05540

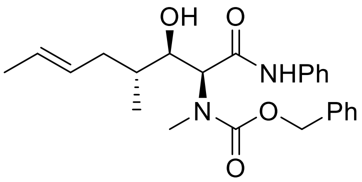

Benzyl ((2S,3R,4R,E)-3-hydroxy-4-methyl-1-oxo-1 (phenylamino)oct-6-en-2-yl)(methyl)carbamate, 25

Additional Info : Peak(s) manually integrated

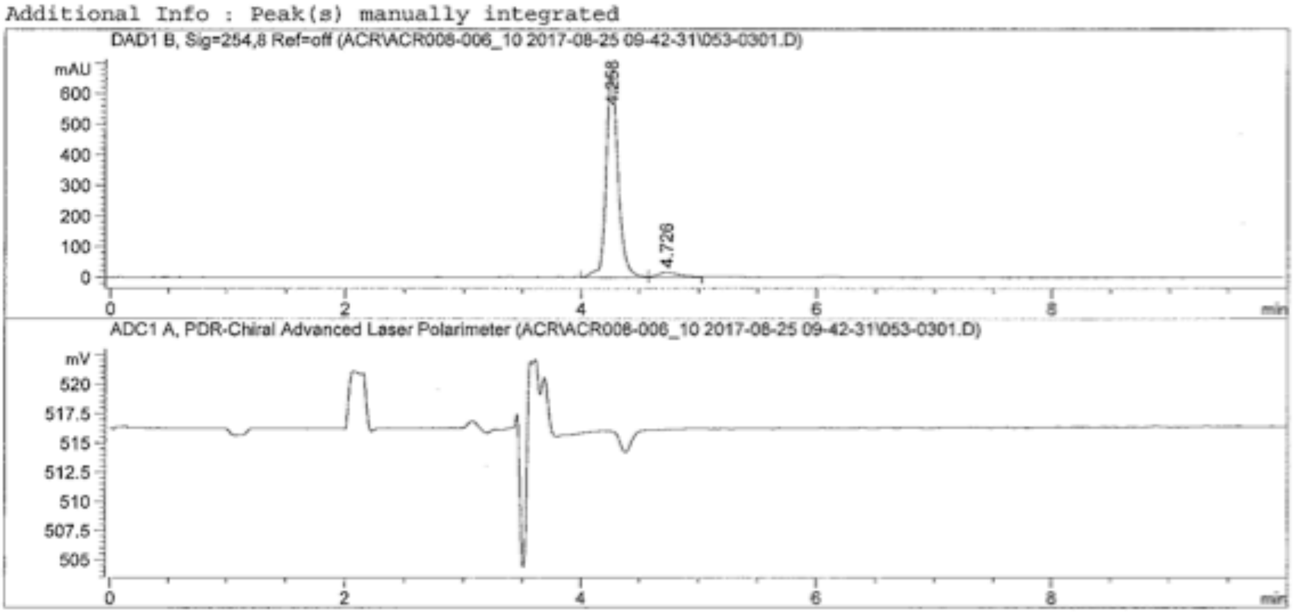

===
Area Percent Report
===

Sorted By : Signal
Multiplier : 1.0000
Dilution : 1.0000
Do not use Multiplier & Dilution Factor with ISTDs

Signal 1: DAD1 B, Sig=254,8 Ref=off

Peak #	RetTime [min]	Type	Width [min]	Area [mAU*s]	Height [mAU]	Area %
1	4.258	VV	0.1108	4936.21484	679.06091	95.0528
2	4.726	VV	0.2097	256.91260	17.45443	4.9472

Totals : 5193.12744 696.51535

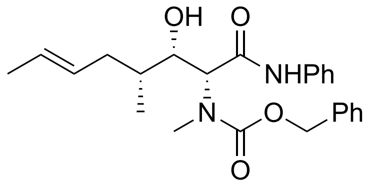

benzyl ((2*R*,3*S*,4*R*,*E*)-3-hydroxy-4-methyl-1-oxo-1-(phenylamino)oct-6-en-2-yl)(methyl)carbamate, 27

Additional Info : Peak(s) manually integrated

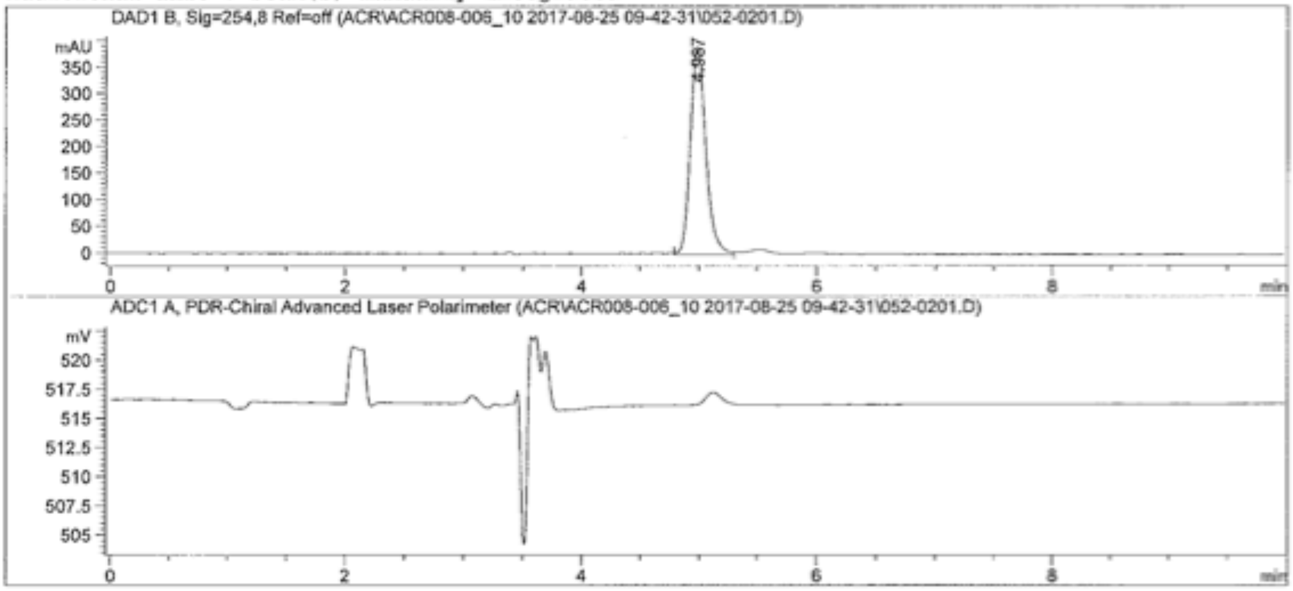

===

Area Percent Report

===

Sorted By : Signal
Multiplier : 1.0000
Dilution : 1.0000
Do not use Multiplier & Dilution Factor with ISTDs

Signal 1: DAD1 B, Sig=254,8 Ref=off

Peak #	RetTime [min]	Type	Width [min]	Area [mAU*s]	Height [mAU]	Area %
1	4.987	VV	0.1405	3568.46777	391.03738	100.0000

Totals : 3568.46777 391.03738

X-Ray crystallography – experimental summary

Compound name: benzyl ((2S,3R)-3-hydroxy-4-methyl-1-oxo-1-(phenylamino)pentan-2-yl)(methyl)carbamate

CCDC Deposition number: 1897285

The single crystal X-ray diffraction studies were carried out on a Bruker D8 Platinum[135] CCD diffractometer equipped with Cu K_α radiation (λ = 1.5478). A 0.217 x 0.053 x 0.051 mm piece of a colorless rod was mounted on a Cryoloop with Paratone oil. Data were collected in a nitrogen gas stream at 125(2) K using ϕ and ϖ scans. Crystal-to-detector distance was 45 mm using variable exposure time (2s-5s) depending on θ with a scan width of 2.0°. Data collection was 98.4% complete to 68.00° in θ. A total of 33864 reflections were collected covering the indices, -11<=h<=10, -12<=k<=14, -19<=l<=19. 3547 reflections were found to be symmetry independent, with a R_{int} of 0.0549. Indexing and unit cell refinement indicated a primitive, orthorhombic lattice. The space group was found to be $P2_12_12_1$. The data were integrated using the Bruker SAINT software program and scaled using the SADABS software program. Solution by direct methods (SHELXT) produced a complete phasing model consistent with the proposed structure.

All nonhydrogen atoms were refined anisotropically by full-matrix least-squares (SHELXL-2014). All carbon bonded hydrogen atoms were placed using a riding model. Their positions were constrained relative to their parent atom using the appropriate HFIX command in SHELXL-2014. All other hydrogen atoms (H-bonding) were located in the difference map. Their relative positions were restrained using DFIX commands and their thermals freely refined. The absolute stereochemistry of the molecule was established by anomalous dispersion using the Parson's method with a Flack parameter of 0.050(34). Crystallographic data are summarized in Table 1.

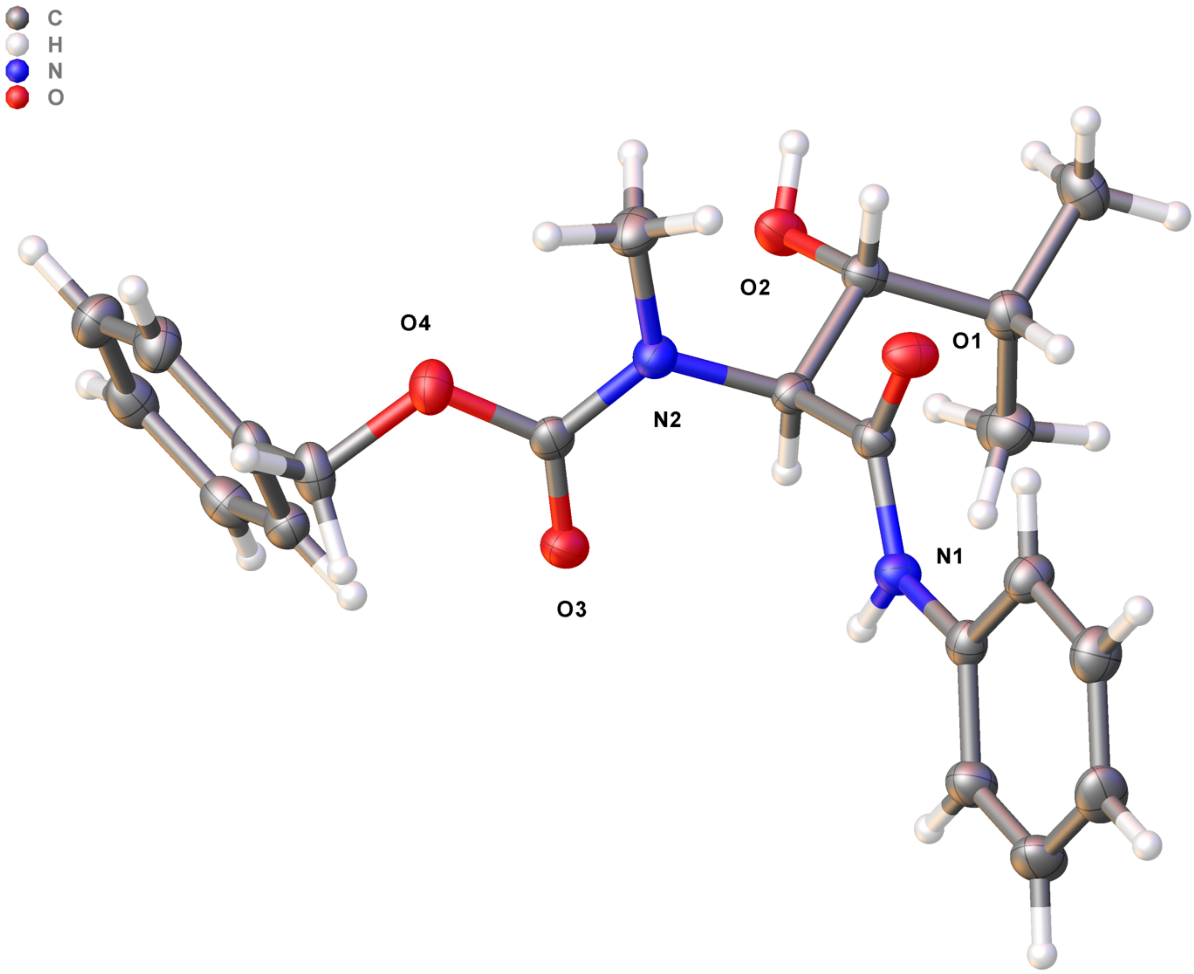
C
H
N
O
O4
O2
O1
N2
N1
O3

Table 1. Crystal data and structure refinement for NIH18.

Identification code	NIH18	
Empirical formula	C21 H26 N2 O4	
Molecular formula	C21 H26 N2 O4	
Formula weight	370.44	
Temperature	125 K	
Wavelength	1.54178 Å	
Crystal system	Orthorhombic	
Space group	$P2_12_12_1$	
Unit cell dimensions	a = 10.2972(4) Å	α= 90°.
	b = 11.7362(4) Å	β= 90°.
	c = 16.2355(6) Å	γ = 90°.
Volume	1962.06(12) $Å^3$	
Z	4	
Density (calculated)	1.254 Mg/m^3	
Absorption coefficient	0.707 mm^{-1}	
F(000)	792	
Crystal size	0.217 x 0.053 x 0.051 mm^3	
Crystal color, habit	Colorless Rod	
Theta range for data collection	4.649 to 68.273°.	
Index ranges	-11<=h<=10, -12<=k<=14, -19<=l<=19	
Reflections collected	33864	
Independent reflections	3547 [R(int) = 0.0549, R(sigma) = 0.0205]	
Completeness to theta = 68.000°	98.4 %	
Absorption correction	Semi-empirical from equivalents	
Max. and min. transmission	0.3201 and 0.2018	
Refinement method	Full-matrix least-squares on F^2	
Data / restraints / parameters	3547 / 2 / 255	
Goodness-of-fit on F^2	1.086	
Final R indices [I>2sigma(I)]	R1 = 0.0252, wR2 = 0.0636	
R indices (all data)	R1 = 0.0256, wR2 = 0.0639	
Absolute structure parameter	0.05(3)	
Extinction coefficient	n/a	
Largest diff. peak and hole	0.113 and -0.179 e.$Å^{-3}$	

References

(1) Misaki, T.; Nagase, R.; Matsumoto, K.; Tanabe, Y. *J. Am. Chem. Soc.* 2005, *127*, 2854.
(2) Zhou, G.; Lim, D.; Coltart, D. M. *Org. Let.* 2008, *10*, 3809.
(3) Seashore-Ludlow, B.; Villo, P.; Somfai, P. *Chem.: Eur. J.* 2012, *18*, 7219.
(4) Katritzky, A. R.; Zhang, Y.; Singh, S. K. *Synthesis* 2003, *2003*, 2795.
(5) Evans, D. A.; Weber, A. E. *J. Am. Chem. Soc* 1986, *108*, 6757.
(6) D. T. Deyo, J. D. Aebi and D. H. Rich, *Synthesis*, 1988, 608-610
